# A Convenient One-Pot Synthesis of a Sterically Demanding Aniline from Aryllithium Using Trimethylsilyl Azide, Conversion to *β*-Diketimines and Synthesis of a *β*-Diketiminate Magnesium Hydride Complex

**DOI:** 10.3390/molecules28227569

**Published:** 2023-11-13

**Authors:** Nikita Demidov, Mateus Grebogi, Connor Bourne, Aidan P. McKay, David B. Cordes, Andreas Stasch

**Affiliations:** EaStCHEM School of Chemistry, University of St Andrews, North Haugh, St Andrews KY16 9ST, UK; nd49@st-andrews.ac.uk (N.D.); mateus0303@icloud.com (M.G.); cb376@st-andrews.ac.uk (C.B.); apm31@st-andrews.ac.uk (A.P.M.); dbc21@st-andrews.ac.uk (D.B.C.)

**Keywords:** aniline synthesis, azides, *β*-diketiminates, magnesium hydride, metal-halogen exchange, organolithium reagents, sterically demanding *N*-ligands, terphenyl ligands, triazenes

## Abstract

This work reports the one-pot synthesis of sterically demanding aniline derivatives from aryllithium species utilising trimethylsilyl azide to introduce amine functionalities and conversions to new examples of a common *N,N*′-chelating ligand system. The reaction of TripLi (Trip = 2,4,6-*i*Pr_3_-C_6_H_2_) with trimethylsilyl azide afforded the silyltriazene TripN_2_N(SiMe_3_)_2_ in situ, which readily reacts with methanol under dinitrogen elimination to the aniline TripNH_2_ in good yield. The reaction pathways and by-products of the system have been studied. The extension of this reaction to a much more sterically demanding terphenyl system suggested that TerLi (Ter = 2,6-Trip_2_-C_6_H_3_) slowly reacted with trimethylsilyl azide to form a silyl(terphenyl)triazenide lithium complex in situ, predominantly underwent nitrogen loss to TerN(SiMe_3_)Li in parallel, which afforded TerN(SiMe_3_)H after workup, and can be deprotected under acidic conditions to form the aniline TerNH_2_. TripNH_2_ was furthermore converted to the sterically demanding *β*-diketimines ^RTrip^nacnacH (=HC{RCN(Trip)}_2_H), with R = Me, Et and *i*Pr, in one-pot procedures from the corresponding 1,3-diketones. The bulkiest proligand was employed to synthesise the magnesium hydride complex [{(^iPrTrip^nacnac)MgH}_2_], which shows a distorted dimeric structure caused by the substituents of the sterically demanding ligand moieties.

## 1. Introduction

Sterically demanding *N*-ligands [1,2,3,4,5,6] have been driving advances in numerous areas of chemical research. In main group chemistry, for example, the introduction of sterically demanding *N*-ligands and related species has led to the discovery of compound classes with low coordination numbers in a variety of oxidation states, which stabilised molecular entities in unusual bonding modes that were found to show unique properties and novel reactivities [7,8,9]. In addition to effects on compound properties and reactivity instilled by the ligand class attached to central elements, steric effects have a strong influence on compound properties, including on coordination numbers and allowing or preventing certain reaction pathways. The interplay of steric demand, including considerations of repulsion from sheer size and ligand shape [10,11], and attractive effects between ligands, substituents, and central elements from London dispersion forces [12,13,14], paints a more complex picture of the effects bulky ligands have on various compound classes. As such, surprising effects on the chemistry of unusual compound classes stabilised by sterically demanding ligands have often been discovered by exploratory investigation of the sterics and electronics of their ligands. For example, in magnesium *β*-diketiminate chemistry, which is of relevance here, relatively small changes in ligand sterics have led to significantly different product outcomes for magnesium hydride [15,16] and low oxidation state magnesium [17,18,19] chemistry.

The introduction of new sterically demanding ligands bearing different substituents to the chemistry of the elements led to novel classes of compounds [7] that, for the bulkiest systems, show coordination numbers down to one. In selected cases, these have displayed unique structures, bonding modes, and reactivities, for example in Al [20], Ge [21], P [22], Sb [23] and Bi [24] species, but the steric bulk can also pose significant new challenges for the syntheses of these new (pro)ligand entities. Due to the increased steric demand in these ligands, common synthetic techniques may prove less applicable to the task, or suitable, convenient starting materials are not readily commercially available. Thus, facile synthetic techniques are required to expand our toolset to access new ligands. Here, we explore the conversion of bulky aryl halides to reactive organolithium compounds, their further transformation to aniline derivatives, the synthesis to a common proligand class, and an example metal complex.

## 2. Results and Discussion

The aim of the first part of the study was to find a convenient one-pot procedure that would allow the synthesis of substituted anilines [25,26,27,28] from aryl lithium compounds with techniques and methods suitable for, and familiar to, the synthetic inorganic and organometallic communities. Many methods for electrophilic amination reactions have been developed [29,30,31,32,33,34], including some to selectively prepare primary amines, but many also show some drawbacks or limitations, and an important consideration for the work described herein was that it could be applied to highly sterically demanding systems. Other synthetic routes to sterically demanding anilines have been successfully developed, but these require either multi-step protocols and/or high pressure set-ups [35,36,37,38] or modify the substituents via palladium-catalysed cross-coupling reactions [39]. For this study we decided on using the 2,4,6-triisopropylphenyl substituent, Trip, due to the commercial availability of starting materials, rarer use of the respective aniline compared with the ubiquitous Dip (2,6-diisopropylphenyl) congener, and the use of isopropyl substituents as suitable groups for ^1^H NMR spectroscopic investigations.

### 2.1. Lithiation of TripBr

**Scheme 1:**
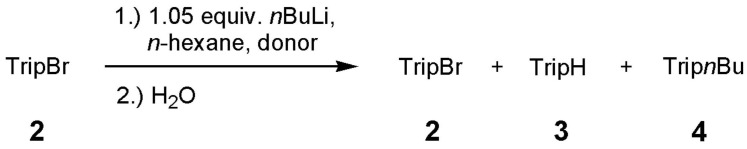
Lithiation of TripBr **2**.

In order to devise a convenient aniline synthesis via an organometallic route, the first consideration was to revisit the generation of TripLi **1** from commercially available TripBr **2**. Some crystallographically characterised TripLi species [40,41] are known, and are prepared using metal-halogen exchange [42,43], with *n*-butyllithium as the lithiating agent. The metal-halogen exchange reaction is an equilibrium system [44] that forms a stabilised mixture, and the thermodynamics and kinetics are influenced by solvent effects [45,46,47], and the steric and electronic nature of the substituents [48,49]. Furthermore, side reactions such as C–C coupling [50] and ether cleavage [51] can lead to consumption of lithium reagent and substrate, potentially hampering efficient conversion to the desired product. In addition, we envisaged that further conversions of in situ generated aryllithium species for the synthesis of sterically demanding systems might require harsher conditions for onward reactivity. As such, we were interested in a quite robust lithiation protocol that can tolerate non-cryogenic conditions, e.g., room temperature, at least for onward reactivity, and would also work for electron-rich aryl substituents. To briefly test and ensure sufficient in situ lithiation for further conversions, TripBr **2** was treated with 1.05 equivalents of *n*BuLi under varying conditions and the mixture was hydrolysed and the product ratio was analysed by ^1^H NMR spectroscopy, see Scheme 1 and Table 1. The relative percentages of the main products TripBr **2**, i.e., unreacted starting material, TripH **3** as a proxy for hydrolysed TripLi **1**, and the coupled product Trip*n*Bu **4** were added to 100% and represent the main products. Traces of TripOH **5**, likely from the reaction of TripLi **1** with trace amounts of air, for example, formed during the quenching process, were also present in some samples. Inspecting the lithiation results summarised in Table 1, diethyl ether (entry 1) as a donor led to insufficient conversion. Using only one or a half equivalent of the more powerful donor solvent THF per Li centre (entries 2 and 3) afforded poor conversion of TripBr **2**. More THF per Li as a donor (2–5 equivalents, entries 4–7) provided good conversion of **2** to **1**, by implication, but also saw increasing quantities of Trip*n*Bu **4** formed from direct C–C coupling [50]. The latter issue could be suppressed by cooling the reaction solution to −20 °C (entry 8), which decreased the coupling to Trip*n*Bu **4** and was beneficial for the lithiation of TripBr **2**, possibly due to forming more reactive lower aggregates for entropic reasons [52] and/or less ether cleavage [51]. To test how competitive ether cleavage is under the conditions, the experiment for entry 7 (Table 1) was repeated, but the *n*BuLi was added to the solvent mixture and left for 30 min at room temperature before TripBr **2** was added and reacted for 30 min before hydrolysis and analysis (entry 9). This experiment provided significant unreacted **2** likely because some *n*BuLi degraded via ether cleavage under these conditions. This highlights that ether cleavage is a significant issue even for relatively low THF concentrations and suggests that these issues are remedied at the lower temperature used in the conditions of entry 8. Going forward, the conditions for entry 8 were used in subsequent sections.

**Scheme 2:**
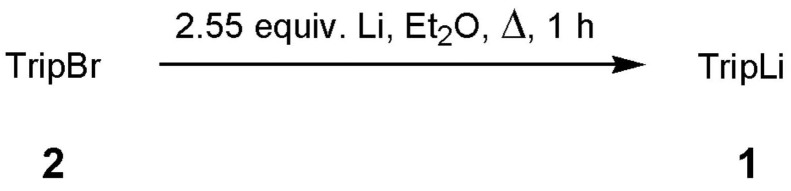
Lithiation of TripBr **2**.

An alternative to using *n*BuLi is the direct reaction of TripBr **2** with lithium metal in diethyl ether for one hour under reflux to TripLi **1**, see Scheme 2, which is straightforward and afforded a good overall yield after in situ conversion to the desired aniline product, vide infra.

### 2.2. Reaction of TripLi 1 with TMSN_3_

Reactions of organometallic compounds and organic azides [29,53,54,55,56] can form triazenes [57], including sterically demanding triazenides [58,59]. In the past, the group of N. Wiberg has studied reactions of s-block organometallics with silylazides and the synthesis and properties of silyltriazenes [60,61,62,63,64,65,66,67]. Subsequently, reactions of specialised organic azides with Grignard or organolithium reagents have been utilised as NH_2_^+^ synthons to successfully afford amines or anilines that show various advantages and limitations. Trimethylsilylmethyl azide [68,69,70] and azidomethyl phenyl sulfide [71,72,73] have been shown to be effective in reactions with Grignard reagents, which can be challenging to form for electron-rich substrates [74], but these azide systems struggle when reacted with organolithium reagents. Diphenylphosphoryl azide affords satisfactory to good yields with either of these classes of organometallic reagents but is more expensive and requires harsh hydrolysis conditions [75]. Vinyl or allyl azides have shown high efficiency [76,77], but are not commercially available and are likely more hazardous to handle [53]. To introduce an alternative that uses a commercially available and relatively stable azide, we proposed that the reaction between an organolithium species, and by implication other electropositive organometallics, and a silylazide would lead to a silyl(organo)triazenide lithium complex, which could be easily worked up to a primary amine in a one-pot procedure. Nucleophilic attack of an organometallic substituent on the (outer) azide nitrogen atom also seemed like a procedure that could be facile even for highly sterically demanding systems, as suggested by the syntheses of bulky triazenides [58,59] and terphenyl azide compounds [78,79,80]. Although syntheses for TripNH_2_ **6** are known [81,82,83,84,85], most involve the synthesis and reduction of TripNO_2_, which has some drawbacks such as expensive and/or hazardous reagents, multi-step protocols, and relies on the accessibility of the nitro derivative.

**Scheme 3:**
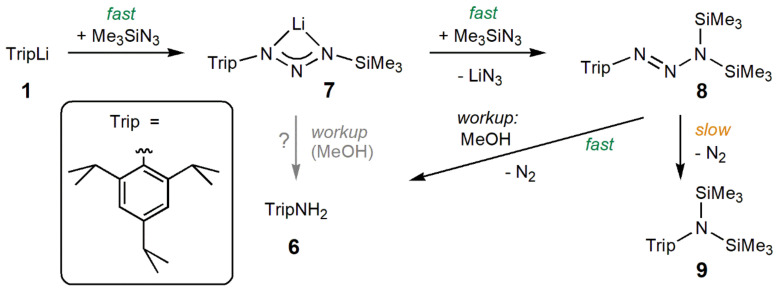
Reactions of TripLi **1** with Me_3_SiN_3_.

**Figure 1:**
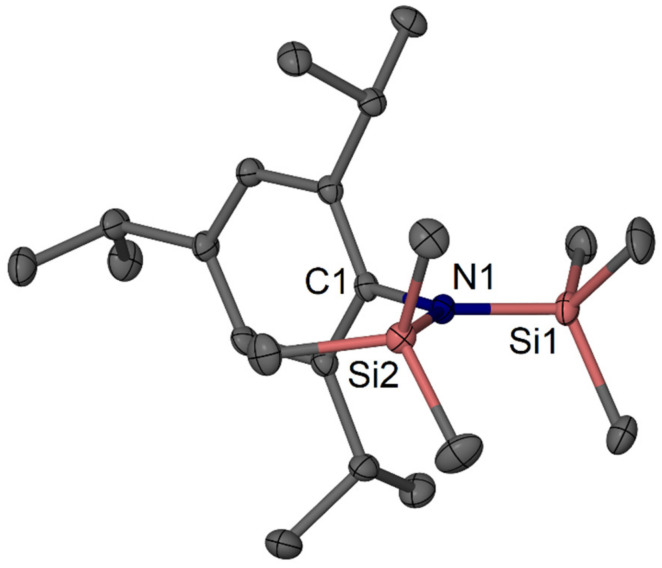
Molecular structure of TripN(SiMe_3_)_2_ **9**, 30% thermal ellipsoids. Hydrogen atoms omitted. Selected bond lengths (Å) and angles (°): Si1–N1 1.7497(19), Si2–N1 1.7554(19), N1–C1 1.450(3); Si1–N1–Si2 123.82(10), C1–N1–Si1 120.42(14), C1–N1–Si2 115.75(14).

Initially, the reaction of TripLi **1**, prepared according to either Scheme 1 or Scheme 2, with one equivalent of trimethylsilyl azide (azidotrimethylsilane, Me_3_SiN_3_) at room temperature proceeded rapidly. We found that simple quenching with (wet) methanol provided rapid gas formation, and crude TripNH_2_ **6** was obtained after workup—an observation that was in line with our expectation of a pathway via an intermediate silyl(organo)triazenide lithium complex, complex **7** in Scheme 3 (grey arrow). The yield of TripNH_2_ **6**, however, did not significantly exceed 40%, and large quantities of TripH **3** were obtained alongside **6**, independent of reaction times. Furthermore, an insoluble precipitate formed during the reaction. Adding further Me_3_SiN_3_, however, increased the yield of TripNH_2_ **6**, and the consistently formed insoluble by-product precipitated from the reaction mixture early on. The latter was identified as LiN_3_ via its properties, NMR, and IR spectroscopic studies, and suggests that further silylation of **7** with Me_3_SiN_3_ occurs, i.e., the azide is acting as a pseudohalide, resulting in the formation of disilyl(organo)triazene TripN_2_N(SiMe_3_)_2_ **8**. Evidence for the formation of **8** comes from an NMR spectroscopic study that shows an intermediate with two chemically identical SiMe_3_ groups by integration and a ^15^N NMR resonance [86,87] detected via a 2D ^1^H-^15^N HMBC NMR experiment of a silyl-bound nitrogen atom at *δ* 187 ppm in deuterated benzene. Derivatives of **8**, such as PhN_2_N(SiMe_3_)_2_, have been obtained by Wiberg previously, and some of these products decomposed with dinitrogen elimination [64]. Treating **8** with methanol gave rapid gas evolution and afforded TripNH_2_ **6**. Reactions performed on a larger scale also afforded small and varying quantities of TripN(SiMe_3_)_2_ **9**, which could be structurally characterised (Figure 1), alongside the main product TripNH_2_ **6**. Compound **9** is typically present in low percentages as a by-product from these reactions, and it is likely that during the synthesis, some TripN_2_N(SiMe_3_)_2_ **8** decomposes and loses dinitrogen to form TripN(SiMe_3_)_2_ **9** (Scheme 3). In contrast to **8**, a ^15^N NMR resonance of *δ* 43 ppm (in CDCl_3_, via a 2D ^1^H/^15^N HMBC NMR experiment) was found for TripN(SiMe_3_)_2_ **9**. As expected, the nitrogen centre in **9** is planar (sum of angles: ca. 360°) in its molecular structure (Figure 1), c.f. the structure of N(SiMe_3_)_3_ [88], and the metrical features are as expected.

A simple aqueous workup of the reaction mixtures afforded crude TripNH_2_ **6** in good, isolated yields. The crude product can be dried under vacuum, removing some volatile by-products, which would include small molecules from ether cleavage and could include smaller amines (e.g., *n*BuNH_2_, b.p. ca. 78 °C) from side reactions. This leaves predominantly TripNH_2_ **6** as the main product, but also the possible high-boiling by-products TripBr **2** from insufficient lithiation, TripH **3** from protonolysis, Trip*n*Bu **4** from C–C coupling, TripOH **5** from oxidation and TripN(SiMe_3_)_2_ **9** from loss of dinitrogen of the intermediate **8**. Gratifyingly, none of the Trip-containing by-products readily reacted with acids and the crude product could be treated with aqueous HCl and petroleum ether or hexane as a two-phase system to afford solid TripNH_3_^+^Cl^−^ **10** (as a hydrate) for purification that could be further washed with petroleum ether or hexane. Treatment with bases regenerated purer TripNH_2_ **6**. Isolated yields of TripNH_2_ **6** after this purification from TripBr were 86% (typically ca. 75–86%) via the *n*BuLi route, and 81% via the Li metal route. In addition, the compound can also be purified by column chromatography on alumina with petroleum ether/dichloromethane.

### 2.3. Extension to a Sterically Demanding Terphenyl System

**Scheme 4:**
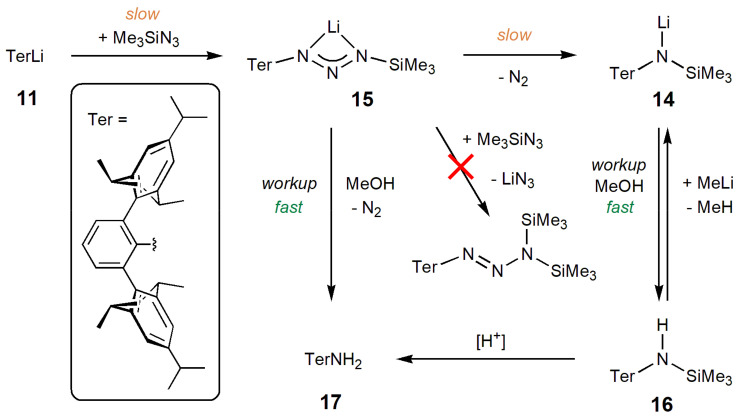
Reactions of TerLi **11** with Me_3_SiN_3_ at room temperature.

**Figure 2:**
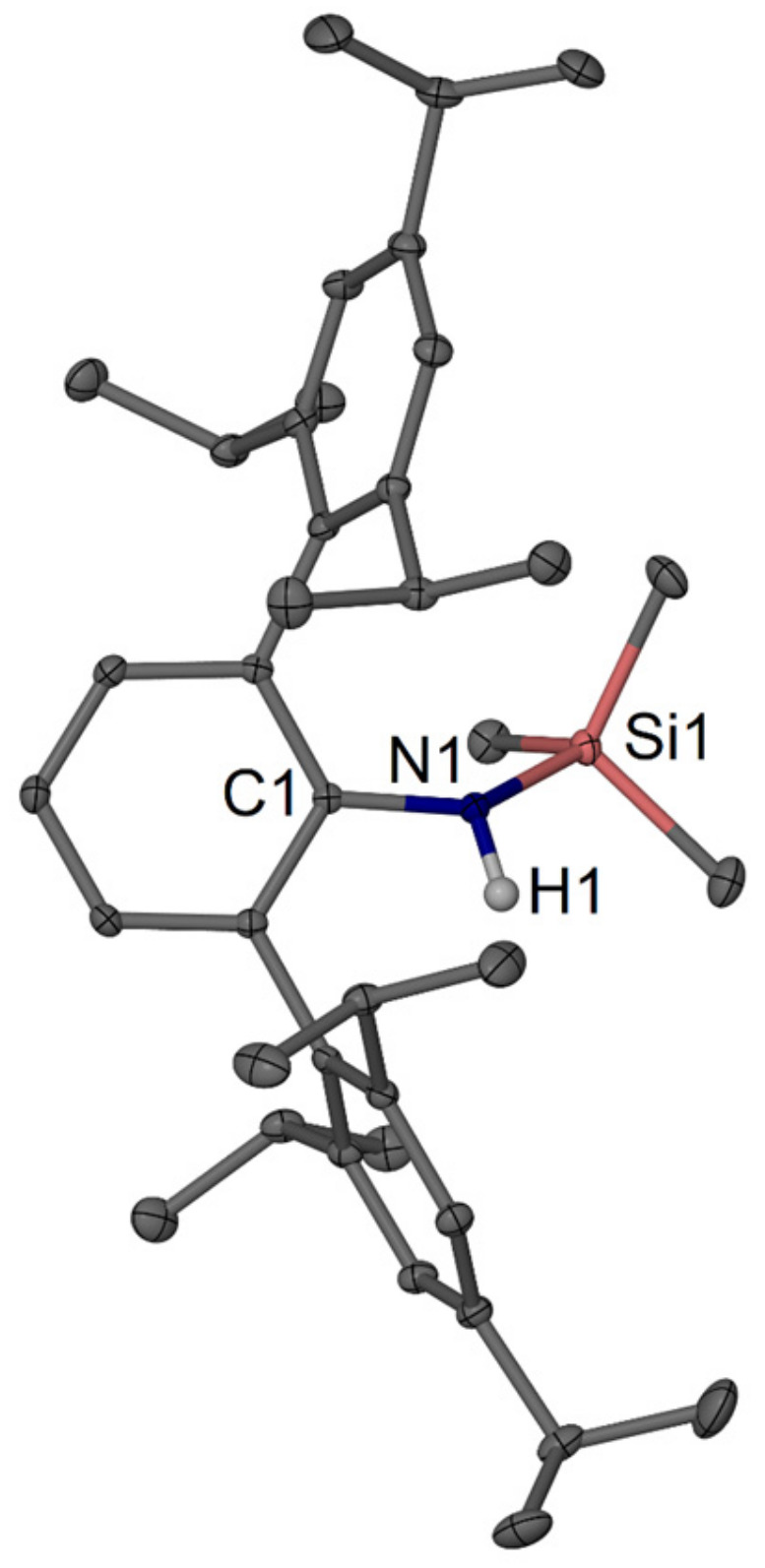
Molecular structure of TerN(SiMe_3_)H **16**, 30% thermal ellipsoids. Only the NH hydrogen atom is shown. Selected bond lengths (Å) and angles (°) for **16**: Si1–N1 1.7439(10), N1–C1 1.4080(14); C1–N1–Si1 129.12(7); metrical data for TerN(SiMe_3_)H∙C_6_H_6_, **16**∙C_6_H_6_: Si1–N1 1.7423(12), N1–C1 1.4061(17); C1–N1–Si1 133.11(10).

To study if the above method can be conveniently transferred to another, more sterically demanding ligand system, we investigated the terphenyl substituent 2,6-bis(2,4,6-triisopropylphenyl)phenyl, Ter, in this reaction [78,80,89]. Initially, TerLi(OEt_2_) **11**(OEt_2_) was prepared from TerI **12** and *n*BuLi [89] to study its reaction with Me_3_SiN_3_ on a small scale by NMR spectroscopy. These experiments showed that the reaction is very slow and that significant quantities of TerH **13** are formed alongside some *N*-containing main product, later identified as a TerN(SiMe_3_)Li **14** derivative. A significant change in the reaction kinetics is not surprising when the steric demand of the system is changed dramatically. It is proposed that the hydrogen on TerH **13** originated from Me_3_SiN_3_ during the reaction and a brief study was undertaken to test the influence of donor solvent addition. When TerLi(OEt_2_) **11**(OEt_2_) was reacted with Me_3_SiN_3_ in deuterated benzene, after four days at room temperature a ratio of TerH **13** to TerN(SiMe_3_)Li **14** of approximately 2:1 was obtained. A reaction with additional five equivalents of the donor solvent diethyl ether under the same conditions was slower (18 days) and the main product ratio changed to approximately 2:3 (**13**:**14**). Changing the donor solvent to THF (5 equivalents) provided an approximate ratio of 1:4 after 7 days at room temperature and 8 h at 60 °C, showing that TerN(SiMe_3_)Li **14** can be afforded as the dominant product (also see Table S1). Other observations that were made showed that, in contrast to the reaction with TripLi **1**, only one equivalent of Me_3_SiN_3_ was required in this bulkier system, and that before **14** forms, the main intermediate, presumed to be TerN_2_N(SiMe_3_)Li **15**, the Ter-equivalent of compound **7** (Scheme 3), was produced, see Scheme 4. Due to the very slow formation of TerN_2_N(SiMe_3_)Li **15** from the starting materials, nitrogen loss from **15**, yielding TerN(SiMe_3_)Li **14**, becomes competitive in parallel. Workup with (wet) methanol afforded TerN(SiMe_3_)H **16** from TerN(SiMe_3_)Li **14**, whereas reaction mixtures with incomplete conversion and larger quantities of the intermediate TerN_2_N(SiMe_3_)Li **15** afforded TerNH_2_ **17** after workup, see Scheme 4 for a summary of these pathways. Some evidence for this was obtained by 2D ^1^H-^15^N HMBC NMR experiments in deuterated benzene where the ^15^N NMR signal of the Me_3_Si-bound nitrogen centre could be measured and compared. These were found at *δ* 317 ppm for the intermediate TerN_2_N(SiMe_3_)Li **15**, *δ* 115 ppm for TerN(SiMe_3_)Li **14** and *δ* 67 ppm for TerN(SiMe_3_)H **16** [76,77]. Furthermore, the reaction of TerN(SiMe_3_)Li **14** to TerN(SiMe_3_)H **16** was found to be reversible; treatment of **14** with an excess of methanol in an NMR tube afforded **16**, and this mixture could then treated be with an excess of solid MeLi to afford **14** again. So far, we found no evidence for further reaction of TerN_2_N(SiMe_3_)Li **15**, with Me_3_SiN_3_ to TerN_2_N(SiMe_3_)_2_ and LiN_3_ (Scheme 4), as was analogously observed for the Trip system (Scheme 3). The molecular structures of TerN(SiMe_3_)H **16**, shown in Figure 2, and the solvate TerN(SiMe_3_)H∙C_6_H_6_ **16**∙C_6_H_6_, were determined and highlight the steric demand around the N atom with a C–N–Si angle of ca. 130°. The molecular structure infers that in solution, the silyl methyl groups of **16** can reside above the flanking Trip-aryls which is likely the reason for the upfield-shifted resonance for the protons of the SiMe_3_ group (*δ* −0.34 ppm).

On a preparative scale, TerI **12** was converted with *n*BuLi in an *n*-hexane and diethyl ether mixture to crude TerLi(OEt_2_) **11**(OEt_2_) and then reacted in situ with Me_3_SiN_3_ in toluene and 10 equivalents of THF at 60 °C for 20 h, followed by workup with (wet) methanol at 0 °C to afford TerN(SiMe_3_)H **16** in 60% isolated yield after recrystallisation from diethyl ether. TerN(SiMe_3_)H **16** has been found to desilylate to TerNH_2_ **17** when treated with aqueous HCl, or slowly in wet chloroform or with silica gel.

### 2.4. Synthesis of ^RTrip^nacnacH Compounds

**Scheme 5:**
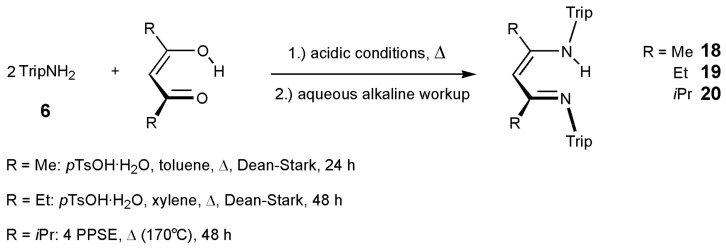
Synthesis of ^RTrip^nacnacH compounds **18**–**20**.

With the aniline TripNH_2_ **6** in hand, we studied its conversion to β-diketimine proligands [2] to progress towards β-diketiminate complexes. The three β-diketimines ^RTrip^nacnacH, =HC{RCN(Trip)}_2_H, with R = Me (**18**), Et (**19**), and *i*Pr (**20**), were prepared by one-pot condensation reactions between TripNH_2_ **6** and appropriate 1,3-diketones under acidic conditions, followed by aqueous workup steps under basic conditions, see Scheme 5. Previously, the ^tBuTrip^nacnacH ligand [83] was prepared via a multi-step route, and a selection of other sterically demanding ^MeAr^nacnacH proligands, where Ar represents a robust substituent larger than Dip, have been reported in recent years [90,91,92,93,94,95,96,97].

The synthesis of ^MeTrip^nacnacH **18**, generally followed an established protocol [98] and afforded an isolated yield of 76%. *β*-Diketimines with an ethyl backbone, ^EtTrip^nacnacH **19** (66% yield), are not common, and we have modified an established procedure [98] used for related ligands. The route to the isopropyl backbone-substituted ^iPrTrip^nacnacH **20** (67% yield) uses a protocol we have recently introduced preparing the related ^iPrDip^nacnacH [99], employing the powerful acidic dehydrating agent polyphosphoric acid trimethylsilylester, PPSE [100,101].

**Figure 3:**
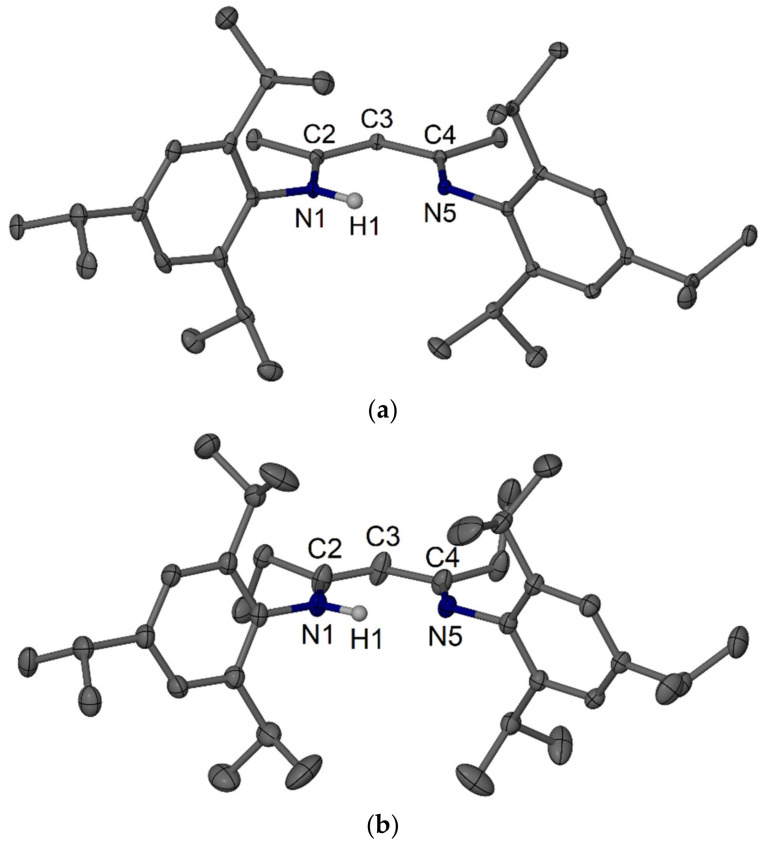

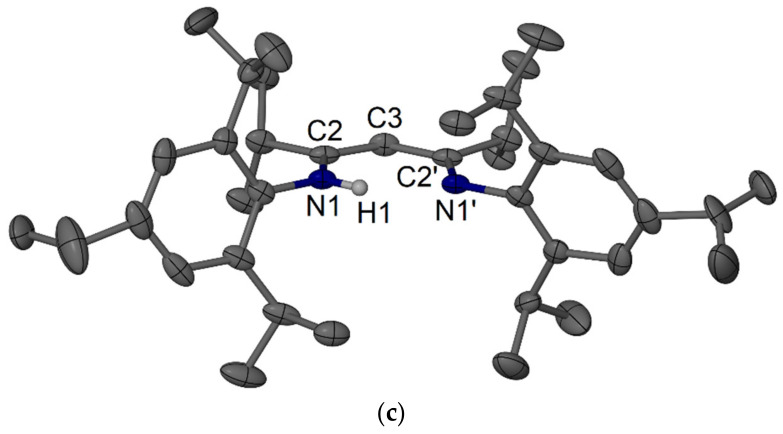
Molecular structures of ^MeTrip^nacnacH∙C_7_H_8_ **18**∙C_7_H_8_ (**a**), ^EtTrip^nacnacH **19** (**b**) and ^iPrTrip^nacnacH **20** (**c**), 30% thermal ellipsoids. The toluene molecule in **18**∙C_7_H_8_ is omitted. Only NH nitrogen atoms are shown including only one NH position for **20**. Selected bond lengths (Å) and angles (°): **18**∙C_7_H_8_: N1–C2 1.341(3), C2–C3 1.383(3), C3–C4 1.424(3), N5–C4 1.315(3); C2–N1–C6 123.8(2), C4–N5–C23 121.0(2), N1–C2–C3 121.3(2), C2–C3–C4 125.4(2), N5–C4–C3 121.0(2); **19**: N1–C2 1.346(3), C2–C3 1.372(3), C4–C3 1.428(3), N5–C4 1.305(3); C2–N1–C6 124.87(18), C4–N5–C25 121.83(17), N1–C2–C3 121.5(2), C2–C3–C4 126.8(2), N5–C4–C3 120.06(19); **20**: N1–C2–1.338(3), C2–C3–1.392(3); N1–C2–C3 121.0(2), C2–N1–C4 122.7(2), C2–C3–C2′ 127.2(3).

The three proligands **18**–**20** were structurally characterised, see Figure 3, and show the expected overall structure for this ligand class. The molecular structures of **18** and **19** each crystallised with a full molecule in the asymmetric unit and show some preference for alternating long and short N–C/C–C bonds in the ligand backbone, and, accordingly, some localisation of the NH hydrogen atom.

### 2.5. Synthesis and Characterization of [{(^iPrTrip^nacnac)MgH}_2_] ***21***

**Scheme 6:**
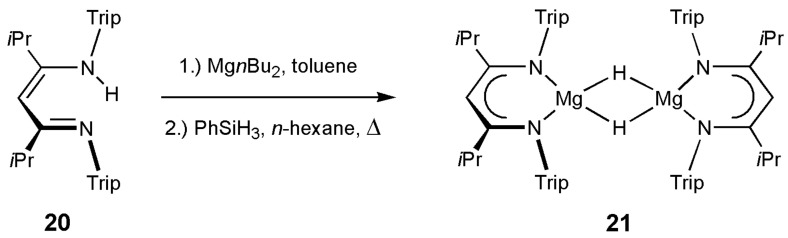
Synthesis of [{(^iPrTrip^nacnac)MgH}_2_] **21**.

**Figure 4:**
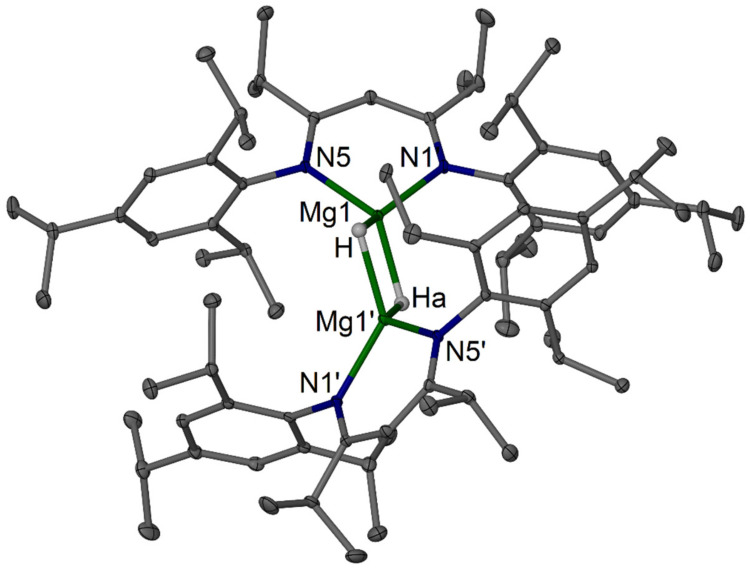
Molecular structures of [{(^iPrTrip^nacnac)MgH}_2_] **21**, 30% thermal ellipsoids. Only MgH hydrogen atom are shown. Selected bond lengths (Å) and angles (°): Mg1–N1 2.0796(12), Mg1–N5 2.0829(12), Mg1–H 2.029(13), Mg1′–H 2.029(13), Mg1–HA 1.979(14), Mg1∙∙∙Mg1 2.9502(9), N1–C2 1.3341(18), C2–C3 1.3981(19), C3–C4 1.3987(19), N5–C4 1.3338(18); N1–Mg1–N5 94.40(5), C2–N1–C6 116.64, C4–N5–C27 116.73(11), N1–C2–C3 124.64(13), N5–C4–C3 124.40(13), C2–C3–C4 132.32(14).

**Figure 5:**
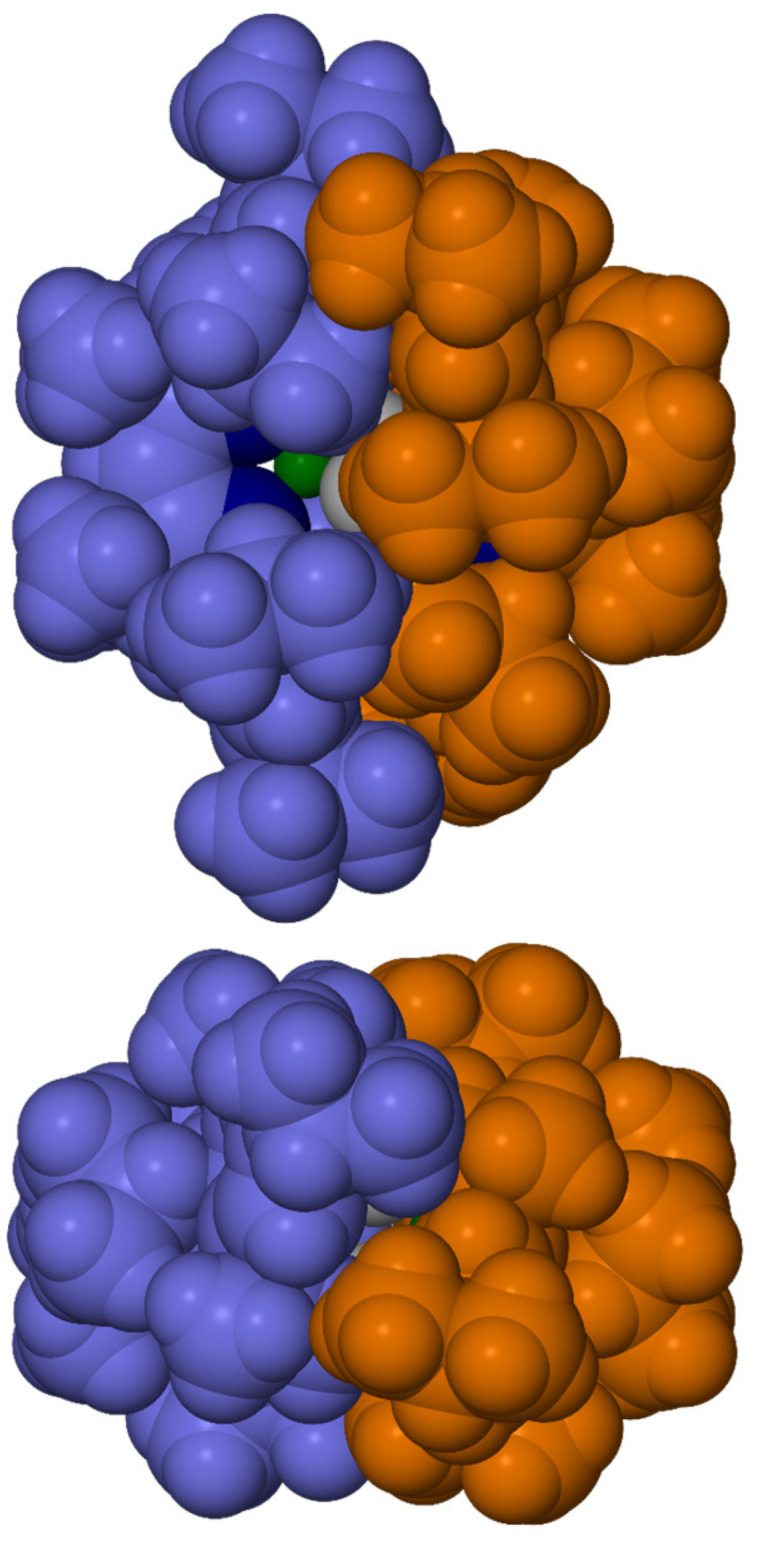
Space-filling model (two views) showing some central atoms (Mg green, N blue, H grey) and the hydrocarbyl groups from the two ligand units in two colours (lavender-purple, rusty orange) showing the “interlocking” isopropyl substituents.

To study the impact of the introduction of the Trip substituent to a *β*-diketiminate system, we used the bulkiest proligand reported herein, ^iPrTrip^nacnacH **20**, to prepare a magnesium hydride complex [16,93,102] for comparison to other related molecules of the type ^RAr^nacnacH [{(^RAr^nacnac)MgH}_2_], where ^RAr^nacnac = {HC{RCN(Ar)} and R is typically an alkyl group and Ar is a sterically demanding aryl substituent. Using an established protocol [103], ^iPrTrip^nacnacH **20** was treated with Mg*n*Bu_2_ in toluene to form the expected intermediate complex [(^iPrTrip^nacnac)Mg*n*Bu]. After removal of all volatiles, the oily residue was taken up in *n*-hexane and reacted for two days at 60 °C with phenyl silane which precipitated clean [{(^iPrTrip^nacnac)MgH}_2_] **21** in 41% isolated yield (Scheme 6). Complex **21** could be recrystallised from hot benzene to form large colourless crystals that were structurally characterised, see Figure 4. The complex crystallised as a dimeric system, as is found for most other related complexes of the type [{(nacnac)MgH}_2_], with a comparable steric bulk [15,93,103,104], although a few examples of the type [(nacnac)MgH] are known with a monomeric solid state structure with a terminal hydride species and three-coordinate Mg centre [18,91]. The least-square-planes of the two essentially planar *β*-diketiminate magnesium chelate rings are rotated by approximately 47.6° relative to each other which must be due to the to the alternating “interlocking” contact of the isopropyl groups of the two ligand units which is visualised in the space-filling model in Figure 5, showing the hydrocarbyl units of both *β*-diketiminates in different colours. For comparison, *β*-diketiminate magnesium chelate rings are approximately co-planar in [{(^MeDip^nacnac)MgH}_2_] [103], approximately orthogonal to each other in [{(^tBuDip^nacnac)MgH}_2_] [104] but show a similar rotation in [{(^MeDIPeP^nacnac)MgH}_2_] (DIPeP = 2,6-di(3-pentyl)phenyl) with ca. 42° [93] between metal-ligand planes to accommodate the various bulky substituents on the ligands. An analysis of the buried volume [11] for the Mg centre in **21** (*V*_buried_ = 53.5%) provided a similar value compared to those for known structurally characterised [{(^RDip^nacnac)MgH}_2_] complexes (*V*_buried_ = 51.1–56.3%), but hints at a more even distribution of the bulk around the metal centre when compared to those of [{(^RDip^nacnac)MgH}_2_] as judged from inspection the distribution in the four quadrants (see Table S2 in the Supporting Information).

In solution, [{(^iPrTrip^nacnac)MgH}_2_] **21** shows ^1^H NMR resonances for a symmetric compound with a sharp singlet at *δ* 3.96 ppm for the magnesium hydride resonance in the expected region. The room temperature ^1^H NMR spectrum shows one broad and two sharp septets, and one broad and three sharp doublets for the isopropyl hydrogen atoms. The broad septet and one broad doublet resonance are associated with one ortho isopropyl group including one methyl group that likely experiences the steric influence from the dimeric interlocked “geometry.” The broad plus one sharp doublet merge above 60 °C and at 80 °C, three resolved septets and three doublets are observed.

## 3. Conclusions

Sterically demanding aryllithium compounds can be converted with trimethylsilyl azide to triazene-class intermediates in a one-pot reaction, installing a nitrogen atom at the aryl group and forming aniline derivatives. For two different systems, one with a bulky Trip (Scheme 3) substituent, and one with an extremely bulky terphenyl substituent (Scheme 4) slightly different (long-lived) intermediates were observed, but the general reactivity is comparable but also influenced by the reaction kinetics resulting from the steric demand of the substituents. An aryllithium can react with trimethylsilyl azide to form silyl(aryl)triazenide lithium, which, for R = Trip, reacted with further trimethylsilyl azide and converted to TripN_2_N(SiMe_3_)_2_ **8**. Upon workup with methanol and resulting in dinitrogen formation, this afforded the aniline TripNH_2_ **6** in good yield. For the bulkier terphenyl system, the in situ generated silyl(aryl)triazenide lithium species did not react with further trimethylsilyl azide. Instead, this lithiated species formed only slowly enough that it (slowly) lost dinitrogen in parallel and predominantly formed TerN(SiMe_3_)H **16** in a one-pot synthesis after treatment with methanol. This silylated aniline derivative is a promising sterically demanding amide proligand in its own right but can also be readily desilylated with acids to afford TerNH_2_ **17**. This work shows the main types of intermediates for two systems with highly different steric demands, and gives practical considerations around their synthesis, optimisation, and workup, with the view that the protocol can be easily extended to other substituents and organometallic species for the synthesis of anilines, primary amines, and silylated derivatives. This includes optimised conditions for metal-halogen exchange with *n*-butyllithium that supress side reactions but allow conversions at relatively high temperatures. Furthermore, TripNH_2_ **6** has been further converted in acid-promoted one-pot condensation reactions to the sterically demanding proligands ^MeTrip^nacnacH **18**, ^EtTrip^nacnacH **19**, and ^iPrTrip^nacnacH **20**, the latter of which was converted to the magnesium hydride complex [{(^iPrTrip^nacnac)MgH}_2_] **21**. The significant effects of the steric influence and dispersion forces of the 16 isopropyl groups in **21** will likely impact compound properties and future reactivity.

## 4. Materials and Methods

### 4.1. Experimental Details

All manipulations were carried out using standard Schlenk and glove box techniques under an atmosphere of high purity argon or dinitrogen. Tetrahydrofuran, diethyl ether, toluene and *n*-hexane were either dried and distilled under inert gas over LiAlH_4_ or taken from an MBraun solvent purification system and degassed prior to use. ^1^H, ^7^Li, ^13^C{^1^H} and ^1^H-^15^N HMBC NMR spectra were recorded on a Bruker AVII 400, Bruker AVIII 500, Bruker AVIII-HD 500 or Bruker AVIII-HD 700 spectrometer (Bruker, Billerica, MA, USA) in deuterated benzene or chloroform and were referenced to the residual ^1^H or ^13^C{^1^H} resonances of the solvent used, external LiCl in D_2_O, or external liquid NH_3_, respectively. Abbreviations: s = singlet, d = doublet, t = triplet, q = quartet, quint = quintet, sext = sextet, sept = septet, br = broad, m = multiplet, e.g., brs, broad singlet; ad denotes an apparent doublet. The IR spectrum was recorded on a neat solid using a Shimadzu IRAfinity 1S IR spectrometer. Melting points were determined in air and are uncorrected. 1-bromo-2,4,6-triisopropylbenzene (TripBr **2**) was degassed and stored over molecular sieves under inert atmosphere. Trimethylsilyl azide (azidotrimethylsilane) and phenylsilane were degassed and stored under inert atmosphere. TerI **12** and TerLi(OEt_2_) **11**(OEt_2_) were prepared according to the literature [89]. All other compounds were used as received from chemical suppliers.

**CAUTION!** Azides are potentially explosive and highly toxic substances, and all manipulations must be carried out by trained workers. Trimethylsilyl azide is considered relatively stable but must not be mixed with acids or water and certain other substances. The aqueous layer during the workup steps below will, or can, contain lithium azide, should be treated accordingly (e.g., collect as a dilute alkaline aqueous solution), and should not be mixed with other waste. A reaction between ionic azides and halogenated reagents and solvents, such as dichloromethane, must be avoided to prevent the formation of explosive azides. The hazards and risks of procedures are dependent on scale. Use suitable gloves when working with azides. Even though no issues were encountered during the work, the use of a blast shield is strongly suggested [53,105,106].

### 4.2. Syntheses and Formation of Trip Compounds (***1**–**6**, **9**, **10***)

#### 4.2.1. General Procedure for the Optimisation of the Lithium-Bromide Exchange

A Schlenk flask was charged with TripBr **2** (1-bromo-2,4,6-triisopropylbenzene; 0.50 mL, 1.97 mmol), *n*-hexane and the amount of THF or diethyl ether to afford the desired solvent ratio as shown in Table 1. The amount of solvent used for each reaction was determined by calculating the total amount of donor solvent and *n*-hexane (accounting for hexanes from *n*BuLi solution) needed to make a 0.395 m solution of TripBr **2** with the desired donor solvent to Li centre ratio. For an example involving 5 eq. of THF per Li see Section 4.2.2. The reaction mixture was brought to the required temperature and *n*BuLi (1.30 mL, 1.6 m solution in hexanes, 1.05 eq.) was added dropwise. The reaction mixture was stirred for 30 min and quenched with H_2_O (ca. 5 mL). All volatiles were removed in vacuo, dichloromethane (ca. 10 mL) and water (ca. 10 mL) were added, and the organic layer was separated. All volatiles were removed in vacuo affording a crude oil that was analysed with ^1^H NMR spectroscopy in CDCl_3_. Since TripBr **2**, TripH **3** and Trip*n*Bu **4** (plus an occasional minor quantity of TripOH **5** from O_2_ capture) are the only Trip-containing products observed, the conversion was calculated directly from the ratio of their aromatic protons, and their sum was assumed as 100% for a qualitative analysis of the lithiation (Table 1).

#### 4.2.2. Synthesis of TripNH_2_ **6**

*Method 1:* A Schlenk flask was charged with TripBr **2** (4.00 mL, 15.79 mmol), *n*-hexane (22.2 mL) and THF (7 mL, ca. 5 THF per Li). The reaction mixture was cooled to −20 °C using a cold bath and *n*BuLi (10.8 mL, 1.6 m solution in hexanes, 1.09 eq.) was added dropwise over approximately two minutes. The reaction mixture was stirred for 30 min at −20 °C and was then placed into a room temperature water bath. After a period of 1–2 min, Me_3_SiN_3_ (4.80 mL, 36.5 mmol, 2.31 eq.) was added dropwise and the reaction mixture was stirred for a further 30 min. A white precipitate of LiN_3_ was formed during that period. The reaction mixture was placed in an ice-water bath (0 °C), the stopper of the flask was removed under a gentle flow of inert gas, and subsequently, methanol (10 mL) was added slowly added over a period of 5 min under vigorous gas evolution. Stirring of the mixture was continued until all gas evolution ceased, after which all volatiles were removed in vacuo and the crude product was redissolved in methanol (10 mL) to ensure complete conversion to aniline **6**. After standing for 10 min, all volatiles were removed in vacuo, and DCM (ca. 30 mL) and water (ca. 30 mL) were added, and organic layer was separated. [*Note:* Be aware that the aqueous layer contains dissolved LiN_3_. Ensure that Me_3_SiN_3_ has reacted and is largely consumed in the procedure; if unsure, use an alkaline solution for carefully quenching of the reaction mixture to ensure no significant quantities of HN_3_, are produced.] Removal of all volatiles in vacuo afforded crude TripNH_2_ **6** as a yellow-orange oil which may be sufficiently pure for some applications.

*Purification:* Various methods of purification could be used (e.g., column chromatography using alumina (90), eluent: petroleum ether (40–60 °C) followed by dichloromethane), however, a batch scale purification method via conversion to an anilinium chloride, TripNH_3_^+^Cl^−^ **10** was found to work best. Conc. aq. HCl (ca. 37%, 12 mL) and petroleum ether (40–60 °C fraction, 10 mL) were added, which produced an off-white precipitate of TripNH_3_^+^Cl^−^ **10** as a hydrate. The resulting suspension was stirred for 10 min, after which the solid (powder) was isolated by filtration and washed with petroleum ether (10 mL). [*Note:* A small and varying quantity of TripN(SiMe_3_)_2_ **9** was obtained in crystalline form by evaporation of the petroleum ether filtrate.] The TripNH_3_^+^Cl^−^ **10** hydrate solid was redissolved in dichloromethane (40 mL) and (saturated) aqueous Na_2_CO_3_ (50 mL) was added, and the resulting mixture was stirred for 30 min. The organic layer was separated, the solvent removed in vacuo, and the product was dried under vacuum and afforded TripNH_2_ **6** of sufficient purity. Yield: 2.98 g (86%).

*Method 2:* A Schlenk flask was charged with lithium granules (280 mg, 0.5% sodium, 4–10 mesh, 40.3 mmol, 2.55 eq.), CBr_4_ for activation (ca. 2–3 mg), diethyl ether (ca. 30 mL) and equipped with a reflux condenser and a nitrogen inlet. A solution of TripBr **2** (4.00 mL, 15.8 mmol) in diethyl ether (ca. 8 mL) was added in portions over 30 min to a stirring suspension of lithium granules which initiated an exothermic reaction. After the addition has been completed, the resulting mixture was refluxed for 60 min, cooled to room temperature, allowed to settle, and filtered. To the filtrate, Me_3_SiN_3_ (4.80 mL, 36.5 mmol, 2.31 eq.) was added dropwise at room temperature and the reaction mixture was stirred for a further 30 min. Workup with methanol and purification were carried out as described above in *Method 1*. Yield: 2.82 g (81%). ^1^H NMR (499.9 MHz, CDCl_3_, 298 K) *δ* = 1.29 (d, *J*_HH_ = 7.0 Hz, 6H, Ar-*p*-CH(C*H*_3_)_2_), 1.33 (d, *J*_HH_ = 6.9 Hz, 12H, Ar-*o*-CH(C*H*_3_)_2_), 2.88 (sept, *J*_HH_ = 6.9 Hz, 1H, Ar-*p*-C*H*(CH_3_)_2_), 2.99 (sept, *J*_HH_ = 6.8 Hz, 2H, Ar-*o*-C*H*(CH_3_)_2_), 3.66 (brs, 2H, N*H*_2_), 6.96 (s, 2H, Ar-*H*).

#### 4.2.3. Data for LiN_3_

The compound is formed during the synthesis of TripNH_2_ **6** and could be isolated directly from the reaction mixture by filtration. The solid also showed a positive flame test for Br suggesting minor LiBr contamination from the reaction mixture. ^7^Li NMR (155.5 MHz, D_2_O, 294 K) *δ* = −0.10 (s, *Li*N_3_). IR (ATR), v~/cm^−1^: 2127 (s).

#### 4.2.4. Data for TripN_2_N(SiMe_3_)_2_ **8**

Solid TripLi **1** was obtained by performing a lithiation of TripBr as descried for the synthesis of **6** above, via *Method 2*, and storing the concentrated diethyl ether solution at −40 °C. After isolation and briefly drying under vacuum, the obtained crystalline material showed approximately 45% TripLi by weight (with the rest assumed to be ^1^H NMR-silent LiBr as judged by integration against an internal standard). TripLi (ca. 3.3 mg, 15.7 μmol) was dissolved in C_6_D_6_ (0.5 mL) in a J. Young’s NMR tube and Me_3_SiN_3_ (4.6 μL, 35 μmol, ca. 2.23 eq.) was added at 20 °C. Analysis by ^1^H NMR spectroscopy showed immediate consumption of TripLi **1** and formation of **8**. ^1^H NMR (700.1 MHz, C_6_D_6_, 295 K) *δ* = 0.34 (s, 18H, Si(C*H*_3_)_3_), 1.26 (d, *J*_HH_ = 6.9 Hz, 6H, Ar-*p*-CH(C*H*_3_)_2_), 1.28 (d, *J*_HH_ = 7.0 Hz, 12H, Ar-*o*-CH(C*H*_3_)_2_), 2.83 (sept, *J*_HH_ = 7.0 Hz, 1H, Ar-*p*-C*H*(CH_3_)_2_), 3.21 (sept, *J*_HH_ = 6.9 Hz, 2H, Ar-*o*-C*H*(CH_3_)_2_), 7.15 (s, 2H, Ar-*H*). ^13^C{^1^H} NMR (176.0 MHz, C_6_D_6_, 295 K) *δ* = 2.0 (Si(*C*H_3_)_3_), 24.2 (Ar-*o*-CH(*C*H_3_)_2_), 24.5 (Ar-*p*-CH(*C*H_3_)_2_), 28.3 (Ar-*o*-*C*H(CH_3_)_2_), 34.8 (Ar-*p*-CH(*C*H_3_)_2_), 121.4 (Ar-*C*), 140.9 (Ar-*C*), 145.7 (Ar-*C*), 146.6 (Ar-*C*). ^1^H-^15^N HMBS NMR (700.1/70.9 MHz, C_6_D_6_, 295 K) *δ* ≈ 187 (TripN_2_*N*(SiMe_3_)_2_).

#### 4.2.5. Data for TripN(SiMe_3_)_2_ **9**

The compound was occasionally isolated during the purification of TripNH_2_ **6** as described above, especially when the reaction was carried out on a multigram scale (>30 mmol). It remains unclear what factors favour its formation, as preliminary experiments with altered stoichiometry or prolonged reaction time have shown no clear pattern, although heat appears to favour dinitrogen elimination. The compound sublimes at ca. 50 °C (ca. 0.05 mbar) affording colourless crystals that were suitable for X-ray crystallographic analysis. ^1^H NMR (700.1 MHz, CDCl_3_, 295 K) *δ* = 0.06 (s, 18H, Si(C*H*_3_)_3_), 1.17 (d, *J*_HH_ = 6.9 Hz, 12H, Ar-*o*-CH(C*H*_3_)_2_), 1.21 (d, *J*_HH_ = 6.9 Hz, 6H, Ar-*p*-CH(C*H*_3_)_2_), 2.82 (sept, *J*_HH_ = 6.9 Hz, 1H, Ar-*p*-C*H*(CH_3_)_2_), 3.41 (sept, *J*_HH_ = 6.9 Hz, 2H, Ar-*o*-C*H*(CH_3_)_2_), 6.84 (s, 2H, Ar-*H*). ^13^C{^1^H} NMR (176.0 MHz, CDCl_3_, 295 K) *δ* = 2.7 (Si(*C*H_3_)_3_), 24.3 (Ar-*p*-CH(*C*H_3_)_2_), 25.3 (Ar-*o*-CH(*C*H_3_)_2_), 27.6 (Ar-*o*-*C*H(CH_3_)_2_), 33.8 (Ar-*p*-CH(*C*H_3_)_2_), 121.4 (Ar-*C*), 140.5 (Ar-*C*), 144.1 (Ar-*C*), 146.2 (Ar-*C*). ^1^H NMR (700.1 MHz, C_6_D_6_, 295 K) *δ* = 0.16 (s, 18H, Si(C*H*_3_)_3_), 1.22 (d, *J*_HH_ = 6.9 Hz, 6H, Ar-*p*-CH(C*H*_3_)_2_), 1.29 (d, *J*_HH_ = 6.9 Hz, 12H, Ar-*o*-CH(C*H*_3_)_2_), 2.79 (sept, *J*_HH_ = 6.9 Hz, 1H, Ar-*p*-C*H*(CH_3_)_2_), 3.58 (sept, *J*_HH_ = 6.9 Hz, 2H, Ar-*o*-C*H*(CH_3_)_2_), 7.05 (s, 2H, Ar-*H*). ^1^H-^15^N HMBS NMR (700.1/70.9 MHz, C_6_D_6_, 295 K) *δ* ≈ 43 (Trip*N*(SiMe_3_)_2_).

#### 4.2.6. Data for TripNH_3_^+^Cl^−^∙1.75 H_2_O, **10**∙1.75 H_2_O

The compound was formed during the purification of TripNH_2_ **6** as described above, and the water content was estimated by integration of the ^1^H NMR spectrum. ^1^H NMR (499.9 MHz, CDCl_3_, 298 K) *δ* = 1.24 (d, *J*_HH_ = 6.9 Hz, 6H, Ar-*p*-CH(C*H*_3_)_2_), 1.30 (d, *J*_HH_ = 6.5 Hz, 12H, Ar-*o*-CH(C*H*_3_)_2_), 1.68 (brs, ca. 3.5H, *H*_2_O), 2.89 (sept, *J*_HH_ = 6.9 Hz, 1H, Ar-*p*-C*H*(CH_3_)_2_), 3.70 (sept, *J*_HH_ = 5.9 Hz, 2H, Ar-*o*-C*H*(CH_3_)_2_), 7.06 (s, 2H, Ar-*H*), 10.30 (brs, 3H, N*H*_3_). ^13^C{^1^H} NMR (125.7 MHz, CDCl_3_, 298 K) *δ* = 24.1 (Ar-*p*-CH(*C*H_3_)_2_), 24.4 (Ar-*o*-CH(*C*H_3_)_2_), 28.6 (Ar-*o*-*C*H(CH_3_)_2_), 34.3 (Ar-*p*-CH(*C*H_3_)_2_), 122.4 (Ar-*C*), 123.5 (Ar-*C*), 142.7 (Ar-*C*), 149.5 (Ar-*C*).

### 4.3. Syntheses and Formation of Ter Compounds (***14**–**17***)

#### 4.3.1. TerN(SiMe_3_)Li **14**

TerLi(OEt_2_) **11**(OEt_2_) (9.5 mg, 16.9 μmol) was dissolved in C_6_D_6_ (0.5 mL) in a J. Young’s NMR tube, after which THF (7.0 μL, 5.1 eq.) and Me_3_SiN_3_ (2.4 μL, 18 μmol, 1.07 eq.) were added. The sample was heated for 24 h at 60 °C, after which analysis by ^1^H NMR spectroscopy showed complete consumption of TerLi∙Et_2_O **11**(OEt_2_) and formation of TerN(SiMe_3_)Li **14**. Further context is described in the main text. ^1^H NMR (700.1 MHz, C_6_D_6_, 295 K) *δ* = −0.18 (s, 9H, Si(C*H*_3_)_3_), 1.24 (d, *J*_HH_ = 6.8 Hz, 12H, Trip-*o*-CH(C*H*_3_)_2_), 1.28 (d, *J*_HH_ = 7.0 Hz, 12H, Trip-*p*-CH(C*H*_3_)_2_), 1.40 (d, *J*_HH_ = 6.9 Hz, 12H, Trip-*o*-CH(C*H*_3_)_2_), 2.84 (sept, 2H, Trip-*p*-C*H*(CH_3_)_2_), 3.47 (m, 4H, Trip-*o*-C*H*(CH_3_)_2_), 6.87 (t, *J*_HH_ = 7.3 Hz, 1H, *p*-C_6_*H*_3_), 7.23 (d, *J*_HH_ = 7.3 Hz, 2H, *m*-C_6_*H*_3_), 7.24 (s, 4H, *m*-Trip). ^7^Li NMR (155.5 MHz, C_6_D_6_) *δ* = −0.81 (N(SiMe_3_)*Li*). ^13^C{^1^H} NMR (176.0 MHz, C_6_D_6_, 295 K) *δ* = 3.3 (Si(*C*H_3_)_3_), 23.8 (Trip-*o*-CH(*C*H_3_)_2_), 24.4 (Trip-*p*-CH(*C*H_3_)_2_), 26.4 (Trip-*o*-CH(*C*H_3_)_2_), 30.5 (Trip-*o*-*C*H(CH_3_)_2_), 34.7 (Trip-*p*-CH(*C*H_3_)_2_), 112.9 (Ar-*C*), 121.7 (Ar-*C*), 131.4 (Ar-*C*), 132.9 (Ar-*C*), 140.9 (Ar-*C*), 147.6 (Ar-*C*), 148.4 (Ar-*C*), 159.0 (Ar-*C*). ^1^H-^15^N HMBS NMR (700.1/70.9 MHz, C_6_D_6_, 295 K) *δ* ≈ 115 (Ter*N*(SiMe_3_)Li).

#### 4.3.2. Partial NMR Data for TerN_2_N(SiMe_3_)Li **15**

Compound TerN_2_N(SiMe_3_)Li **15** is formed as an intermediate during the reaction between TerLi(OEt_2_) **11**(OEt_2_) and Me_3_SiN_3_ in C_6_D_6_ with or without additional donor solvents. It was observed using ^1^H NMR spectroscopy by tracking the apparent triplet at 6.98 ppm (*p*-C_6_*H*_3_), doublet at 7.12 ppm (*m*-C_6_*H*_3_) and a singlet at 0.03 ppm (Si(C*H*_3_)_3_). ^1^H-^15^N HMBS NMR (700.1/70.9 MHz, C_6_D_6_, 295 K) *δ* ≈ 317 (TerN_2_*N*(SiMe_3_)Li).

#### 4.3.3. Partial NMR Data for TerH

TerH is formed as a by-product during the reaction between TerLi(OEt_2_) **11**(OEt_2_) and Me_3_SiN_3_ in C_6_D_6_ with or without the addition of donor solvents. It was observed using ^1^H NMR spectroscopy by tracking the singlet at 7.21 ppm (*m*-Trip).

#### 4.3.4. Synthesis of TerN(SiMe_3_)H **16**

A Schlenk flask was charged with TerI **12** (1.00 g, 1.64 mmol), *n*-hexane (ca. 25 mL) and Et_2_O (ca. 10 mL). The reaction mixture was cooled to −50 °C using a cold bath and *n*BuLi (1.03 mL, 1.6 M solution in hexanes, ca. 1 eq.) was added dropwise. The reaction mixture was allowed to slowly warm to room temperature and stirred for an additional 2 h. All volatiles were removed in vacuo affording a white powder which was extensively dried for 1h, after which toluene (ca. 20 mL), THF (1.34 mL, 10 eq.) and Me_3_SiN_3_ (0.30 mL, 2.28 mmol, 1.4 eq.) were added. The resulting mixture was heated for 20 h to 60 °C, after which it was placed in an ice bath and methanol (10 mL) was added slowly over a period of 5 min, after which all volatiles were removed in vacuo. Dichloromethane (ca. 20 mL) and water (ca. 20 mL) were added, and organic layer was separated. Removal of volatiles in vacuo afforded crude product as a white solid, which was purified by recrystallisation from cold (room temperature to −40 °C) diethyl ether and afforded as two crops. Crystals suitable for X-ray crystallographic analysis were obtained upon storage of concentrated diethyl ether solution at 6 °C. Yield = 0.56 g (60%). ^1^H NMR (700.1 MHz, C_6_D_6_, 295 K) *δ* = −0.34 (s, 9H, Si(C*H*_3_)_3_), 1.16 (d, *J*_HH_ = 6.8 Hz, 12H, Trip-*o*-CH(C*H*_3_)_2_), 1.29 (d, *J*_HH_ = 7.0 Hz, 12H, Trip-*p*-CH(C*H*_3_)_2_), 1.38 (d, *J*_HH_ = 6.9 Hz, 12H, Trip-*o*-CH(C*H*_3_)_2_), 2.87 (sept, *J*_HH_ = 6.9 Hz, 2H, Trip-*p*-C*H*(CH_3_)_2_), 3.06 (sept, *J*_HH_ = 6.8 Hz, 4H, Trip-*o*-C*H*(CH_3_)_2_), 3.24 (s, 1H, N(SiMe_3_)*H*), 6.92 (t, *J*_HH_ = 7.5 Hz, 1H, *p*-C_6_*H*_3_), 7.19 (d, *J*_HH_ = 7.5 Hz, 2H, *m*-C_6_*H*_3_), 7.23 (s, 4H, *m*-Trip). ^13^C{^1^H} NMR (176.0 MHz, C_6_D_6_, 295 K) *δ* = 0.4 (Si(*C*H_3_)_3_), 23.8 (Trip-*o*-CH(*C*H_3_)_2_), 24.4 (Trip-*p*-CH(*C*H_3_)_2_), 26.2 (Trip-*o*-CH(*C*H_3_)_2_), 30.9 (Trip-*o*-*C*H(CH_3_)_2_), 34.9 (Trip-*p*-CH(*C*H_3_)_2_), 119.3 (Ar-*C*), 121.6 (Ar-*C*), 131.3 (Ar-*C*), 131.4 (Ar-*C*), 135.7 (Ar-*C*), 144.9 (Ar-*C*), 148.0 (Ar-*C*), 149.0 (Ar-*C*). ^1^H-^15^N HMBS NMR (700.1/70.9 MHz, C_6_D_6_, 295 K) *δ* ≈ 67 (Ter*N*(SiMe_3_)H).

#### 4.3.5. Deprotection of TerN(SiMe_3_)H ***16***: Synthesis of TerNH_2_ **17**

NMR scale experiments have shown that TerN(SiMe_3_)H **16** could be deprotected with silica or by prolonged standing in wet CDCl_3_ to obtain the corresponding aniline TerNH_2_ **17**, a known compound [78]. On a large scale it is more convenient to deprotect **16** using aqueous HCl. TerN(SiMe_3_)H **16** (120 mg, 0.20 mmol) was dissolved in diethyl ether (12 mL), and conc. aq. HCl (37%, 0.5 mL) was added dropwise, and the resulting solution was stirred at room temperature for 1 h, after which aqueous (saturated) Na_2_CO_3_ solution (20 mL) was added, and the resulting mixture was stirred for 30 min. The organic layer was separated, and the aqueous layer was extracted with dichloromethane (ca. 15 mL). The organic fractions were combined, and the solvent was removed in vacuo, affording solid TerNH_2_ **17** which was isolated and dried under vacuum. (*Note:* an additional extraction step with dichloromethane may be required if insoluble material is present, e.g., NaHCO_3_). Yield = 90 mg (86%). ^1^H NMR (500.1 MHz, CDCl_3_, 295 K) *δ* = 1.10 (d, *J*_HH_ = 6.9 Hz, 12H, Trip-*o*-CH(C*H*_3_)_2_), 1.12 (d, *J*_HH_ = 6.8 Hz, 12H, Trip-*o*-CH(C*H*_3_)_2_), 1.30 (d, *J*_HH_ = 6.9 Hz, 12H, Trip-*p*-CH(C*H*_3_)_2_), 2.75 (sept, *J*_HH_ = 6.8 Hz, 4H, Trip-*o*-C*H*(CH_3_)_2_), 2.94 (sept, *J*_HH_ = 6.9 Hz, 2H, Trip-*p*-C*H*(CH_3_)_2_), 3.14 (brs, 2H, N*H*_2_), 6.81 (t, *J*_HH_ = 7.4 Hz, 1H, *p*-C_6_*H*_3_), 6.96 (d, *J*_HH_ = 7.4 Hz, 2H, *m*-C_6_*H*_3_), 7.08 (s, 4H, *m*-Trip).

### 4.4. Syntheses of ^RTrip^nacnacH Compounds ***18**–**20***

#### 4.4.1. ^MeTrip^nacnacH **18**

A round bottom flask was charged with pentane-2,4-dione (1.07 mL, 10.42 mmol, 1.0 equiv), *para*-toluenesulfonic acid monohydrate, *p*TsOH∙H_2_O (2.18 g, 11.46 mmol, 1.10 eq.), TripNH_2_ **6** (4.81 g, 21.9 mmol, 2.10 eq.) and toluene (90 mL), and equipped with a Dean-Stark trap and a reflux condenser. The flask was placed in an oil bath and the reaction mixture was heated under reflux for 24 h to remove the water. Subsequently, most of the solvent (ca. 85 mL) was distilled off via the Dean-Stark trap leaving a dark brown oily residue. The oil was taken up in saturated aqueous Na_2_CO_3_ solution (ca. 100 mL) and dichloromethane (ca. 100 mL) and stirred until two clear phases formed. The organic layer was separated, and all volatiles were reduced in vacuo giving a dark viscous oil. Addition of methanol (20 mL) results in almost immediate precipitation of ^MeTrip^nacnacH **18** as an off-white solid which is subsequently isolated by filtration and washed with cold methanol (ca. 20 mL). Concentrating the supernatant solution to ca. 10 mL and storing at −40 °C afforded additional colourless crystals of **6** that were suitable for X-ray crystallographic analysis. Yield = 4.00 g (76%). M.p. 158–161 °C. ^1^H NMR (500.1 MHz, CDCl_3_, 295 K) *δ* = 1.12 (d, *J*_HH_ = 6.9 Hz, 12H, Ar-*o*-CH(C*H*_3_)_2_), 1.21 (d, *J*_HH_ = 6.9 Hz, 12H, Ar-*o*-CH(C*H*_3_)_2_), 1.25 (d, *J*_HH_ = 6.9 Hz, 12H, Ar-*p*-CH(C*H*_3_)_2_), 1.73 (s, 6H, NCC*H*_3_), 2.87 (sept, *J*_HH_ = 6.9 Hz, 2H, Ar-*p*-*CH*(CH_3_)_2_), 3.09 (sept, *J*_HH_ = 6.9 Hz, 4H, Ar-*o*-C*H*(CH_3_)_2_), 4.85 (s, 1H, NC(CH_3_)C*H*), 6.95 (s, 4H, Ar-*H*), 12.15 (s, 1H, N*H*). ^1^H NMR (400.1 MHz, C_6_D_6_, 294 K) *δ* = 1.21 (d, *J*_HH_ = 6.9 Hz, 12H, Ar-*o*-CH(C*H*_3_)_2_), 1.29 (d, *J*_HH_ = 7.0 Hz, 12H, Ar-*o*-CH(C*H*_3_)_2_), 1.30 (d, *J*_HH_ = 6.9 Hz, 12H, Ar-*p*-CH(C*H*_3_)_2_), 1.71 (s, 6H, NCC*H*_3_), 2.88 (sept, *J*_HH_ = 6.9 Hz, 2H, Ar-*p*-*CH*(CH_3_)_2_), 3.35 (sept, *J*_HH_ = 6.9 Hz, 4H, Ar-*o*-C*H*(CH_3_)_2_), 4.90 (s, 1H, NC(CH_3_)C*H*), 7.17 (s, 4H, Ar-*H*), 12.61 (s, 1H, N*H*). ^13^C{^1^H} NMR (100.6 MHz, C_6_D_6_, 295 K): *δ* = 20.8 (NC*C*H_3_), 23.7 (Ar-*o*-CH(*C*H_3_)_2_), 24.6 (Ar-*o*-CH(*C*H_3_)_2_ or Ar-*p*-CH(*C*H_3_)_2_), 24.6 (Ar-*o*-CH(*C*H_3_)_2_ or Ar-*p*-CH(*C*H_3_)_2_), 28.8 (Ar-*o*-*C*H(CH_3_)_2_), 34.8 (Ar-*p*-*C*H(CH_3_)_2_), 94.1 (NC(CH_3_)*C*H), 121.4 (Ar-*C*), 139.2 (Ar-*C*), 142.7 (Ar-*C*), 145.9 (Ar-*C*), 161.7 (N*C*CH_3_).

#### 4.4.2. ^EtTrip^nacnacH **19**

A round bottom flask was charged with heptane-3,5-dione (1.45 mL, 1.37 g, 10.42 mmol), *para*-toluenesulfonic acid monohydrate, *p*TsOH∙H_2_O (2.24 g, 11.46 mmol, 1.10 eq.), TripNH_2_ **6** (4.94 g, 21.93 mmol, 2.10 eq.) and xylene (mixture of isomers, 90 mL), and equipped with a Dean-Stark trap, a reflux condenser, and a nitrogen inlet with oil bubbler. The flask was placed in an oil bath and nitrogen gas was blown through the apparatus for a few minutes to displace most of the air. The reaction mixture was refluxed under a very slow nitrogen flow for 48 h to remove the water. Subsequently, the majority of the solvent (ca. 80 mL) was distilled off via the Dean-Stark trap leaving a dark brown oily residue. The oil was taken up in a saturated aqueous Na_2_CO_3_ solution (ca. 100 mL) and dichloromethane (ca. 100 mL) and stirred until two clear phases formed. The organic layer was separated, and all volatiles were reduced in vacuo giving a dark viscous oil that was dried under vacuum. Addition of methanol (30 mL) with prolonged sonication resulted in the precipitation of ^EtTrip^nacnacH **19** as an off-white solid which was subsequently isolated by filtration and washed with cold methanol (ca. 20 mL). Colourless crystals suitable for X-ray crystallographic analysis were obtained by redissolving **19** in boiling methanol and subsequent cooling to room temperature. Yield = 3.77 g (66%). M.p. 142–145 °C. ^1^H NMR (500.1 MHz, CDCl_3_, 295 K) *δ* = 1.08 (t, *J*_HH_ = 7.6 Hz, 6H, NCCH_2_C*H*_3_)_,_ 1.10 (d, *J*_HH_ = 6.8 Hz, 12H, Ar-*o*-CH(C*H*_3_)_2_), 1.19 (d, *J*_HH_ = 6.9 Hz, 12H, Ar-*o*-CH(C*H*_3_)_2_), 1.25 (d, *J*_HH_ = 6.9 Hz, 12H, Ar-*p*-CH(C*H*_3_)_2_), 2.05 (q, *J*_HH_ = 7.6 Hz, 4H, NCC*H*_2_CH_3_), 2.87 (sept, *J*_HH_ = 6.9 Hz, 2H, Ar-*p*-*CH*(CH_3_)_2_), 3.07 (sept, *J*_HH_ = 6.9 Hz, 4H, Ar-*o*-C*H*(CH_3_)_2_), 4.91 (s, 1H, NC(CH_2_CH_3_)C*H*), 6.94 (s, 4H, Ar-*H*), 12.14 (s, 1H, N*H*). ^1^H NMR (499.9 MHz, C_6_D_6_, 298 K) 0.98 (t, *J*_HH_ = 7.6 Hz, 6H, NCCH_2_C*H*_3_)_,_ 1.22 (d, *J*_HH_ = 6.8 Hz, 12H, Ar-*o*-CH(C*H*_3_)_2_), 1.30 (d, *J*_HH_ = 6.9 Hz, 12H, Ar-*p*-CH(C*H*_3_)_2_), 1.30 (d, *J*_HH_ = 6.9 Hz, 12H, Ar-*o*-CH(C*H*_3_)_2_), 2.13 (q, *J*_HH_ = 7.6 Hz, 4H, NCC*H*_2_CH_3_), 2.88 (sept, *J*_HH_ = 6.9 Hz, 2H, Ar-*p*-*CH*(CH_3_)_2_), 3.38 (sept, *J*_HH_ = 6.9 Hz, 4H, Ar-*o*-C*H*(CH_3_)_2_), 5.09 (s, 1H, NC(CH_2_CH_3_)C*H*), 7.18 (s, 4H, Ar-*H*), 12.64 (s, 1H, N*H*). ^13^C{^1^H} NMR (125.7 MHz, C_6_D_6_, 298 K): *δ* = 12.1 (NCCH_2_*C*H_3_), 23.6 (Ar-*o*-CH(*C*H_3_)_2_), 24.6 (Ar-*p*-CH(*C*H_3_)_2_), 25.0 (Ar-*o*-CH(*C*H_3_)_2_), 26.8 (NC*C*H_2_CH_3_), 28.7 (Ar-*o*-*C*H(CH_3_)_2_), 34.8 (Ar-*p*-*C*H(CH_3_)_2_), 89.1 (NC(CH_2_CH_3_)*C*H), 121.4 (Ar-*C*), 138.8 (Ar-*C*), 142.8 (Ar-*C*), 145.8 (Ar-*C*), 166.9 (N*C*CH_3_).

#### 4.4.3. ^iPrTrip^nacnacH **20**

A Schlenk flask with reflux condenser and nitrogen inlet was charged with P_4_O_10_ (10.1 g, 35.6 mmol) and hexamethyldisiloxane (25.0 mL, 117.6 mmol), and the mixture was dissolved in dry dichloromethane (25 mL). The reaction mixture was heated to reflux for 2 h under a gentle flow of nitrogen before cooling to 20 °C. All volatiles were removed in vacuo, affording a colourless, viscous syrup of PPSE. 2,6-dimethylheptane-3,5-dione (1.80 mL, 1.64 g, 10.5 mmol) and TripNH_2_ **6** (4.70 g, 21.42 mmol, 2.04 eq.) were added to the flask under a gentle flow of nitrogen. The reaction mixture was then slowly heated to 170 °C and stirred for 48 h at this temperature. The reaction mixture was then cooled to ca. 95 °C and an aqueous NaOH solution (8.0 g in 100 mL) was carefully (exothermic reaction!) and slowly added via the top of the reflux condenser with vigorous stirring. After cooling, the formed solid residue was extracted with dichloromethane (ca. 80 mL), the organic layer was separated off, and all volatiles were removed in vacuo. Addition of methanol (25 mL) resulted in almost immediate precipitation of ^iPrTrip^nacnacH **20** as an off-white solid which was subsequently isolated by filtration and washed with cold methanol (ca. 20 mL). Colourless crystals suitable for X-ray crystallographic analysis were obtained by storing an *n*-hexane solution of **20** at 6 °C for two months. Yield = 3.90 g (67%). M.p. 204–207 °C. ^1^H NMR (500.1 MHz, CDCl_3_, 295 K) *δ* = 1.08 (ad, 24H, Ar-*o*-CH(C*H*_3_)_2_ and NC(CH(C*H*_3_)_2_), 1.22 (d, *J*_HH_ = 6.9 Hz, 12H, Ar-*o*-CH(C*H*_3_)_2_), 1.25 (d, *J*_HH_ = 6.9 Hz, 12H, Ar-*p*-CH(C*H*_3_)_2_), 2.43 (sept, *J*_HH_ = 6.8 Hz, 2H, NC(C*H*(CH_3_)_2_)), 2.87 (sept, *J*_HH_ = 6.9 Hz, 2H, Ar-*p*-CH(CH_3_)_2_), 3.07 (sept, *J*_HH_ = 6.8 Hz, 4H, Ar-*o*-CH(CH_3_)_2_), 4.89 (s, 1H, NC(CH(CH_3_)_2_)C*H*), 6.93 (s, 4H, Ar-*H*), 11.76 (s, 1H, N*H*). ^1^H NMR (499.9 MHz, C_6_D_6_, 298 K) *δ* = 1.05 (d, *J*_HH_ = 6.8 Hz, 12H, NC(CH(C*H*_3_)_2_)), 1.23 (d, *J*_HH_ = 6.8 Hz, 12H, Ar-*o*-CH(C*H*_3_)_2_), 1.29 (d, *J*_HH_ = 6.9 Hz, 12H, Ar-*p*-CH(C*H*_3_)_2_), 1.34 (d, *J*_HH_ = 6.9 Hz, 12H, Ar-*o*-CH(C*H*_3_)_2_), 2.60 (sept, *J*_HH_ = 6.8 Hz, 2H, NC(C*H*(CH_3_)_2_)), 2.86 (sept, *J*_HH_ = 7.0 Hz, 2H, Ar-*p*-C*H*(CH_3_)_2_), 3.39 (sept, *J*_HH_ = 6.9 Hz, 4H, Ar-*o*-C*H*(CH_3_)_2_), 5.12 (s, 1H, NC(CH(CH_3_)_2_)C*H*), 7.18 (s, 4H, Ar-*H*), 12.36 (s, 1H, N*H*). ^13^C{^1^H} NMR (125.7 MHz, C_6_D_6_, 298 K): *δ* = 22.2 (NC(CH(*C*H_3_)_2_)), 23.6 (Ar-*o*-CH(*C*H_3_)_2_), 24.6 (Ar-*p*-CH(*C*H_3_)_2_), 25.7 (Ar-*o*-CH(*C*H_3_)_2_), 28.4 (Ar-*o*-*C*H(CH_3_)_2_), 30.4 (NC(*C*H(CH_3_)_2_)), 34.7 (Ar-*p*-*C*H(CH_3_)_2_), 84.0 (NC(CH(CH_3_)_2_)*C*H), 121.5 (Ar-*C*), 138.4 (Ar-*C*), 142.9 (Ar-*C*), 145.6 (Ar-*C*), 171.8 (N*C*(CH(CH_3_)_2_)).

### 4.5. Synthesis of [{(^iPrTrip^nacnac)MgH}_2_] ***21***

A J Youngs flask was charged with ^iPrTrip^nacnacH **20** (1.00 g, 1.79 mmol) and toluene (ca. 20 mL). The resulting solution was cooled using an ice bath and Mg*n*Bu_2_ (2.33 mL, 1.0 M solution in heptane, 1.30 eq.) was added. The solution was then briefly heated to 50 °C for ca. 30 min and then stirred at room temperature for 16 h. The resulting reaction mixture was reduced in vacuo, redissolved in *n*-hexane (ca. 20 mL), a very small quantity of an insoluble precipitate was filtered off, and phenylsilane (0.29 mL, 2.35 mmol, 1.32 eq.) was added. The resulting mixture was heated to 60 °C for 48 h producing a fine white precipitate of **21**, that was isolated by hot filtration, washed with *n*-hexane (ca. 10 mL) and dried under vacuum. Colourless crystals of **21** suitable for X-ray crystallographic analysis were obtained by cooling a saturated solution in benzene from 60 °C to room temperature. Yield = 0.43 g (41%). ^1^H NMR (499.9 MHz, C_6_D_6_, 298 K) *δ* = 0.94 (d, *J*_HH_ = 6.7 Hz, 24H, NCCH(C*H*_3_)_2_), 1.00 (brs, 24H, Ar-*o*-CH(C*H*_3_)_2_), 1.34 (d, *J*_HH_ = 7.1 Hz, 24H, Ar-*o*-CH(C*H*_3_)_2_), 1.34 (d, *J*_HH_ = 7.0 Hz, 24H, Ar-*p*-CH(C*H*_3_)_2_), 2.53 (sept, *J*_HH_ = 6.8 Hz, 4H, NCC*H*(CH_3_)_2_), 2.89 (sept, *J*_HH_ = 7.1 Hz, 4H, Ar-*p*-C*H*(CH_3_)_2_), 3.19 (brsept, 8H, Ar-*o*-C*H*(CH_3_)_2_), 3.96 (s, 2H, Mg-*H*), 4.88 (s, 2H, NC(CH(CH_3_)_2_)C*H*), 7.08 (s, 8H, Ar-*H*). ^1^H NMR (499.9 MHz, C_6_D_6_, 353 K) *δ* = 0.99 (ad, 48H, Ar-*o*-CH(C*H*_3_)_2_ and NCCH(C*H*_3_)_2_), 1.30 (d, *J*_HH_ = 6.8 Hz, 24H, Ar-*o*-CH(C*H*_3_)_2_), 1.33 (d, *J*_HH_ = 6.9 Hz, 24H, Ar-*p*-CH(C*H*_3_)_2_), 2.54 (sept, *J*_HH_ = 6.6 Hz, 4H, NCC*H*(CH_3_)_2_), 2.88 (sept, *J*_HH_ = 7.0 Hz, 4H, Ar-*p*-C*H*(CH_3_)_2_), 3.17 (sept, *J*_HH_ = 6.9 Hz, 8H, Ar-*o*-C*H*(CH_3_)_2_), 3.93 (s, 2H, Mg-*H*), 4.91 (s, 2H, NC(CH(CH_3_)_2_)C*H*), 7.05 (s, 8H, Ar-*H*). ^13^C{^1^H} NMR (125.7 MHz, C_6_D_6_, 353 K): *δ* = 23.3 (NC(CH(*C*H_3_)_2_) or Ar-*o*-CH(*C*H_3_)_2_), 24.2 (Ar-*o*-CH(*C*H_3_)_2_ or Ar-*p*-CH(*C*H_3_)_2_), 24.4 (Ar-*o*-CH(*C*H_3_)_2_ or Ar-*p*-CH(*C*H_3_)_2_), 26.3 (NC(CH(*C*H_3_)_2_) or Ar-*o*-CH(*C*H_3_)_2_), 28.1 (Ar-*o*-*C*H(CH_3_)_2_), 32.0 (NC(*C*H(CH_3_)_2_)), 34.5 (Ar-*p*-*C*H(CH_3_)_2_), 85.5 (NC(CH(CH_3_)_2_)*C*H), 121.9 (Ar-*C*), 142.5 (Ar-*C*), 143.2 (Ar-*C*), 144.8 (Ar-*C*), 179.9 (N*C*(CH(CH_3_)_2_)).

### 4.6. X-Ray Crystallographic Details

X-ray diffraction data for compounds **16**, **16**∙C_6_H_6_, **18**∙C_7_H_8_, **20**, **21** were collected using a Rigaku FR-X Ultrahigh Brilliance Microfocus RA generator/confocal optics with XtaLAB P200 diffractometer [Mo Kα radiation (λ = 0.71073 Å)]. Diffraction data for compounds **9** and **19** were collected using a Rigaku MM-007HF High Brilliance RA generator/confocal optics with XtaLAB P100 or P200 diffractometers [Cu Kα radiation (λ = 1.54187 Å)]. Data for all compounds analysed were collected and processed (including correction for Lorentz, polarization, and absorption) using CrysAlisPro. [107] Structures were solved by dual space (SHELXT) [108] or direct (SIR2011) [109] methods. All structures were refined by full-matrix least-squares against *F*^2^ (SHELXL-2019/3) [110]. Non-hydrogen atoms were refined anisotropically, and hydrogen atoms were refined using a riding model except for those on nitrogen atoms in **16**, **16**∙C_6_H_6_, **18**∙C_7_H_8_, and **19**, which were located from the difference Fourier map and refined isotropically subject to a distance restraint. The hydride hydrogen atoms in **21** were also located from the difference Fourier map and refined isotropically without distance restraints. All calculations were performed using the Olex2 [111] interface. Selected crystallographic data are presented below. CCDC 2301678–2301684 contains the supplementary crystallographic data for this paper. These data can be obtained free of charge from The Cambridge Crystallographic Data Centre via www.ccdc.cam.ac.uk/structures.

Crystal data for TripN(SiMe_3_)_2_ **9**: CCDC 2301680, C_21_H_41_NSi, *M* = 363.73, colourless prism, 0.06 × 0.05 × 0.04 mm^3^, orthorhombic, space group *Aea*2 (No. 41), *a* = 37.6202(5), *b* = 12.11603(15), *c* = 15.74019(18) Å, *V* = 7174.49(16) Å^3^, *Z* = 12, *D*_c_ = 1.010 g cm^−3^, *F*_000_ = 2424, *μ* = 1.343 mm^−1^, *T* = 173 K, 2*θ*_max_ = 151.4°, 66,014 reflections collected, 7404 unique (*R*_int_ = 0.0494). Final *GoF* = 1.020, *R*_1_ = 0.0339, *wR*_2_ = 0.0863, *R* indices based on 7141 reflections with *I* > 2*σ*(*I*) (refinement on *F*^2^), 359 parameters, 22 restraints. The molecule crystallised with 1.5 molecules in the asymmetric unit. The 4-isopropyl group in the half molecule is disordered and was refined with two positions for each atom using geometry restraints.

Crystal data for TerN(SiMe_3_)H **16**: CCDC 2301681, C_39_H_59_NSi, *M* = 569.96, colourless prism, 0.16 × 0.11 × 0.04 mm^3^, monoclinic, space group *P*2_1_/*c* (No. 14), *a* = 19.3572(3), *b* = 9.47187(15), *c* = 19.6361(3) Å, *b* = 95.6547(14)°, *V* = 3582.73(10) Å^3^, *Z* = 4, *D*_c_ = 1.057 g cm^−3^, *F*_000_ = 1256, *μ* = 0.091 mm^−1^, *T* = 173 K, 2*θ*_max_ = 58.8°, 75,801 reflections collected, 8820 unique (*R*_int_ = 0.0310). Final *GoF* = 1.035, *R*_1_ = 0.0413, *wR*_2_ = 0.1035, *R* indices based on 7244 reflections with *I* > 2*σ*(*I*) (refinement on *F*^2^), 410 parameters, 4 restraints. The molecule crystallised with a full molecule in the asymmetric unit. One 4-isopropyl group is disordered and was refined with two positions for the atoms of the outer methyl groups using geometry restraints.

Crystal data for TerN(SiMe_3_)H∙C_6_H_6_ **16**∙C_6_H_6_: CCDC 2301683, C_45_H_65_NSi, *M* = 648.07, colourless plate, 0.07 × 0.06 × 0.02 mm^3^, monoclinic, space group *P*2_1_/*n* (No. 14), *a* = 9.1204(2), *b* = 19.8896(4), *c* = 22.6572(5) Å, *b* = 92.2103(19)°, *V* = 4106.97(15) Å^3^, *Z* = 4, *D*_c_ = 1.048 g cm^−3^, *F*_000_ = 1424, *μ* = 0.086 mm^−1^, *T* = 125 K, 2*θ*_max_ = 58.3°, 178,448 reflections collected, 10365 unique (*R*_int_ = 0.0671). Final *GoF* = 1.017, *R*_1_ = 0.0490, *wR*_2_ = 0.1076, *R* indices based on 7382 reflections with *I* > 2*σ*(*I*) (refinement on *F*^2^), 454 parameters, 7 restraints. The molecule crystallised with a full molecule and one benzene molecule in the asymmetric unit. One 2-isopropyl group is disordered and was refined with two positions for the atoms of one methyl group and the methine-H using geometry restraints.

Crystal data for ^MeTrip^nacnacH∙C_7_H_8_ **18**∙C_7_H_8_: CCDC 2301684, C_42_H_62_N_2_, *M* = 594.93, colourless prism, 0.12 × 0.04 × 0.03 mm^3^, orthorhombic, space group *Pbca* (No. 61), *a* = 17.3978(6), *b* = 17.1598(6), *c* = 24.9732(8) Å, *V* = 7455.6(4) Å^3^, *Z* = 8, *D*_c_ = 1.060 g cm^−3^, *F*_000_ = 2624, *μ* = 0.060 mm^−1^, *T* = 120 K, 2*θ*_max_ = 58.6°, 160,281 reflections collected, 9438 unique (*R*_int_ = 0.1475). Final *GoF* = 1.022, *R*_1_ = 0.0952, *wR*_2_ = 0.1760, *R* indices based on 5281 reflections with *I* > 2*σ*(*I*) (refinement on *F*^2^), 416 parameters, 1 restraint. The compound crystallised with one full molecule plus one toluene molecule in the asymmetric unit.

Crystal data for ^EtTrip^nacnacH **19**: CCDC 2301678, C_37_H_58_N_2_, *M* = 530.85, colourless plate, 0.12 × 0.11 × 0.01 mm^3^, monoclinic, space group *P*2_1_/*c* (No. 14), *a* = 9.2583(6), *b* = 25.5248(15), *c* = 15.1348(8) Å, *b* = 99.624(6)°, *V* = 3526.3(4) Å^3^, *Z* = 4, *D*_c_ = 1.000 g cm^−3^, *F*_000_ = 1176, *μ* = 0.421 mm^−1^, *T* = 173 K, 2*θ*_max_ = 141.3°, 30,594 reflections collected, 6182 unique (*R*_int_ = 0.0831). Final *GoF* = 1.060, *R*_1_ = 0.0547, *wR*_2_ = 0.1336, *R* indices based on 3814 reflections with *I* > 2*σ*(*I*) (refinement on *F*^2^), 390 parameters, 9 restraints. The compound crystallised with a full molecule in the asymmetric unit. One backbone ethyl group is disordered and was modelled and refined with two positions for each atom.

Crystal data for ^iPrTrip^nacnacH **20**: CCDC 2301679, C_39_H_62_N_2_, *M* = 558.90, colourless prism, 0.12 × 0.06 × 0.04 mm^3^, orthorhombic, space group *Ibca* (No. 73), *a* = 16.1089(8), *b* = 16.8884(10), *c* = 26.3413(18) Å, *V* = 7166.2(7) Å^3^, *Z* = 8, *D*_c_ = 1.036 g cm^−3^, *F*_000_ = 2480, *μ* = 0.059 mm^−1^, *λ* = 0.71073 Å, *T* = 125 K, 2*θ*_max_ = 58.3°, 38,250 reflections collected, 4425 unique (*R*_int_ = 0.0410). Final *GoF* = 1.032, *R*_1_ = 0.0850, *wR*_2_ = 0.1897, *R* indices based on 2675 reflections with *I* > 2*σ*(*I*) (refinement on *F*^2^), 224 parameters, 34 restraints. The compound crystallised with half a molecule in the asymmetric unit. The 4-isopropyl group is disordered and was modelled with two positions for each atom using geometry restraints.

Crystal data for [{(^iPrTrip^nacnac)MgH}_2_] **21**: CCDC 2301682, C_39_H_62_N_2_Mg, *M* = 1166.42, colourless block, 0.09 × 0.09 × 0.03 mm^3^, orthorhombic, space group *Pbcn* (No. 60), *a* = 16.0213(5), *b* = 17.4646(5), *c* = 25.6440(7) Å, *V* = 7175.3(4) Å^3^, *Z* = 4, *D*_c_ = 1.080 g cm^−3^, *F*_000_ = 2576, *μ* = 0.077 mm^−1^, *T* = 125 K, 2*θ*_max_ = 58.2°, 77,501 reflections collected, 8645 unique (*R*_int_ = 0.0521). Final *GoF* = 1.031, *R*_1_ = 0.0484, *wR*_2_ = 0.1125, *R* indices based on 6107 reflections with *I* > 2*σ*(*I*) (refinement on *F*^2^), 399 parameters, 0 restraints. This compound crystallised with half a molecule in the asymmetric unit.

## Figures and Tables

**Table 1:** Lithiation study of TripBr **2**.

Entry	Donor, Equiv. per Li ^a^	Temperature, *T*	TripBr 2	TripH 3 (c.f. TripLi 1)	Trip*n*Bu 4
1	Et_2_O, 17:1	r.t.	41.5	58.5	~0
2	THF, 0.5:1	r.t.	92.2	7.8	~0
3	THF, 1:1	r.t.	68.0	32.0	~0
4	THF, 2:1	r.t.	23.2	74.8	2.0
5	THF, 3:1	r.t.	12.9	83.3	3.8
6	THF, 4:1	r.t.	9.8	84.7	5.4
7	THF, 5:1	r.t.	6.6	85.7	7.7
8	THF, 5:1	−20 °C	trace	>97	trace
9	THF, 5:1 (+*n*BuLi first) ^b^	r.t.	36.1	61.8	2.1

^a^ 1.05 equivalents of *n*BuLi solution were added dropwise to TripBr in *n*-hexane plus donor solvent as given to afford a 0.395 m reaction solution, stirred for 30 min, then hydrolysed with water and the organic residues were analysed by ^1^H NMR spectroscopy (CDCl_3_), reporting the percentages of the products **2**, **3** and **4** from their relative ratios. The experiments were conducted with the same reagent concentrations as single experiments only to allow for a brief study to find favourable conditions. Estimated error +/− 1–2%. In addition, trace amount of TripOH, presumably from traces of oxygen were present in some samples. ^b^ *n*BuLi was first reacted with the solvent mixture for 30 min before TripBr was added and the experiment continued.

## Data Availability

X-ray crystallographic data are available via the CCDC; please see the X-ray Section 4.5. The research data (NMR spectroscopy) supporting this publication can be accessed at https://doi.org/10.17630/419a7764-7072-412a-b008-115c696fd9cd.

## Supplementary Materials

The following supporting information can be downloaded at: https://www.mdpi.com/article/10.3390/molecules28227569/s1; Table S1: In-situ NMR scale study of reaction between TerLi(OEt2) 11(OEt2) and Me3SiN3; IR spectrum (Figure S1), NMR spectroscopy (Figure S2–S38), Buried volume information, Table S2: Buried volume for [{(RArnacnac)MgH}2] complexes for the four quadrants and in total and Figures S39–S40.
