# Supplementary material for: A Convenient One-Pot Synthesis of a Sterically Demanding Aniline from Aryllithium Using Trimethylsilyl Azide, Conversion to β-Diketimines and Synthesis of a β-Diketiminate Magnesium Hydride Complex
Source: Molecules. 2023 Nov 13;28(22):7569. doi: 10.3390/molecules28227569 (PMC10673297; doi:10.3390/molecules28227569)
Supplement: Supplementary file 1 [file molecules-28-07569-s001.zip › molecules-2699386-supplementary.pdf]

Supporting Information

for

A convenient one-pot synthesis of a sterically demanding aniline from aryllithium using trimethylsilyl azide, conversion to *β*-diketimines and synthesis of a *β*-diketiminate magnesium hydride complex

Nikita Demidov[1], Mateus Grebogi[1], Connor Bourne[1], Aidan P. McKay[1], David B. Cordes[1], Andreas Stasch[1,*]

1 EaStCHEM School of Chemistry, University of St Andrews, North Haugh, St Andrews, KY16 9ST, United Kingdom. E-mail: as411@st-andrews.ac.uk.

1 Table S1 page 2

2 IR spectrum page 2

3 NMR spectroscopy page 3

4 Buried volume information page 40

1 Table S1

Table S1. In-situ NMR scale study of reaction between TerLi(OEt_2) **11**(OEt_2) and Me_3SiN_3.

entry	Extra donor added, (equiv.)[a]	Conditions	TerH **13**	TerN($SiMe_3$)Li **14**
1	None	4 days at r.t.	67.1	32.9
2	Et_2O, (5)	18 days at r.t.	38.3	61.7
3	THF, (5)	7 days at r.t, followed by 8 h at 60°C	19.3	80.7

[a]TerLi(OEt_2) **11**(OEt_2) was dissolved in C_6D_6 (0.5 mL) in a J. Young's NMR tube, after which a donor solvent, as given, and Me_3SiN_3 were added. The sample was treated under conditions specified and monitored by ^{1}H NMR spectroscopy, reporting the percentages of products **13** and **14** from their relative ratios. The experiments were conducted using similar reagent concentrations (*ca.* 0.034M for **11**(OEt_2)) as single experiments only. Estimated error +/- 1-2 %. In addition, trace amount of TerH were already present in the starting material TerLi(OEt_2) **11**(OEt_2).

2 IR spectrum

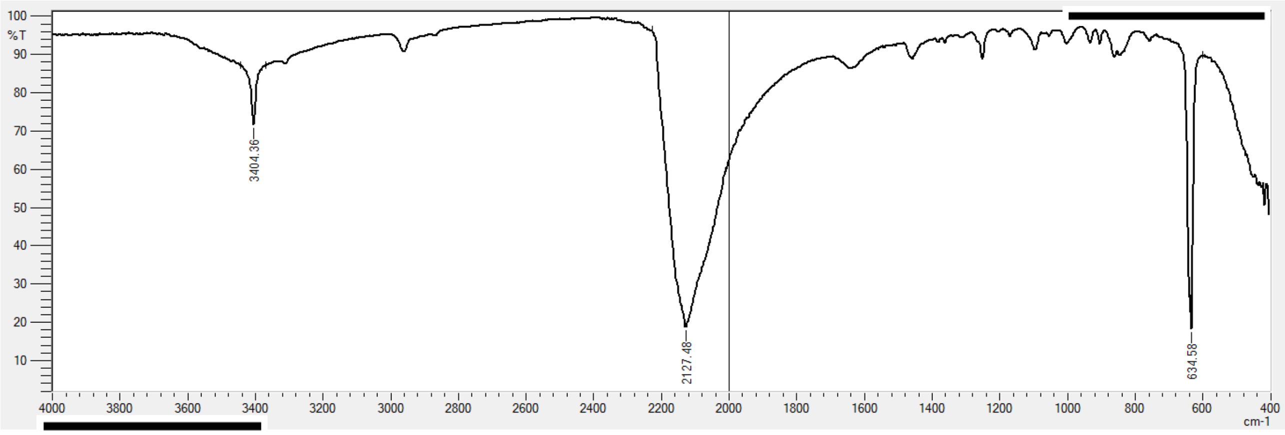

Figure S1: IR spectrum (ATR) of the LiN_3 by-product.

3 NMR spectroscopy

NMR spectra were recorded in deuterated benzene or deuterated chloroform and further details are given in the figure captions and the main manuscript. In some samples, small resonances of residual solvent and silicone grease (as an impurity, e.g., at δ0.29 ppm in ^{1}H NMR spectra in C_6D_6, at δ0.07 ppm in $CDCl_3$) may be present.

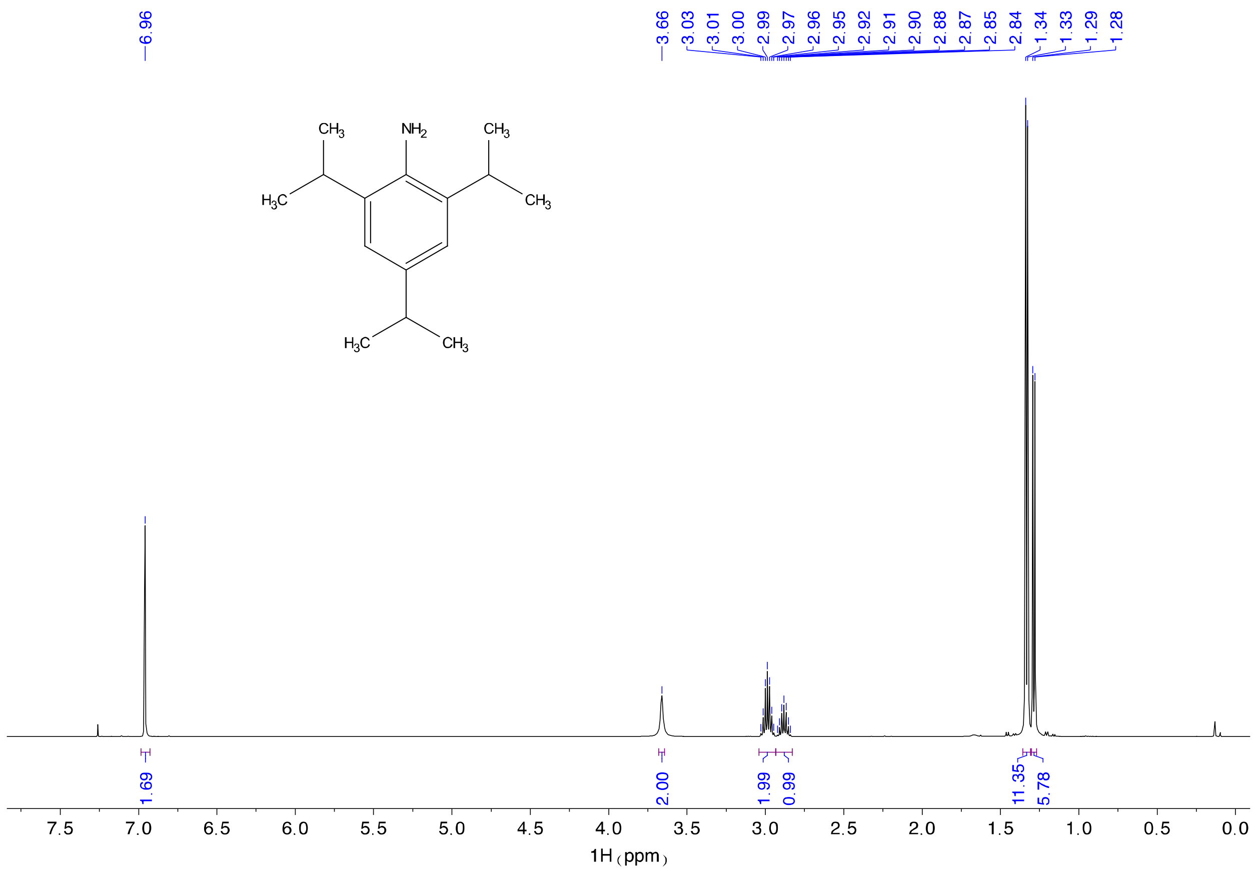

Figure S2. ^{1}H NMR spectrum (499.9 MHz, $CDCl_3$, 298 K) of TripNH$_2$ **6** (purified by column chromatography on alumina, eluent: petroleum ether (40-60°C) followed by dichloromethane).

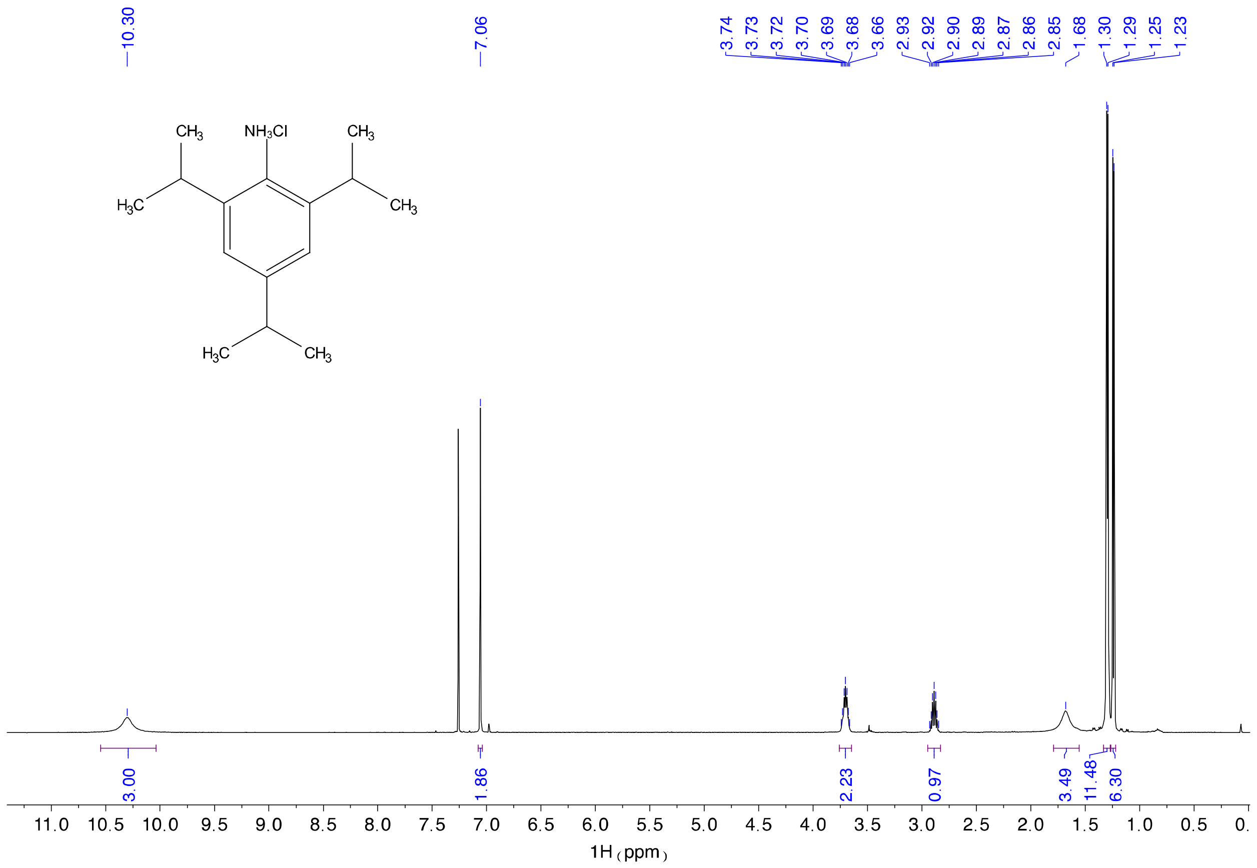

Figure S3. ^{1}H NMR spectrum (499.9 MHz, $CDCl_3$, 298 K) of $TripNH_3^{+}Cl^{-}\cdot 1.75\ H_2O$, **10**$\cdot 1.75\ H_2O$ (as prepared).

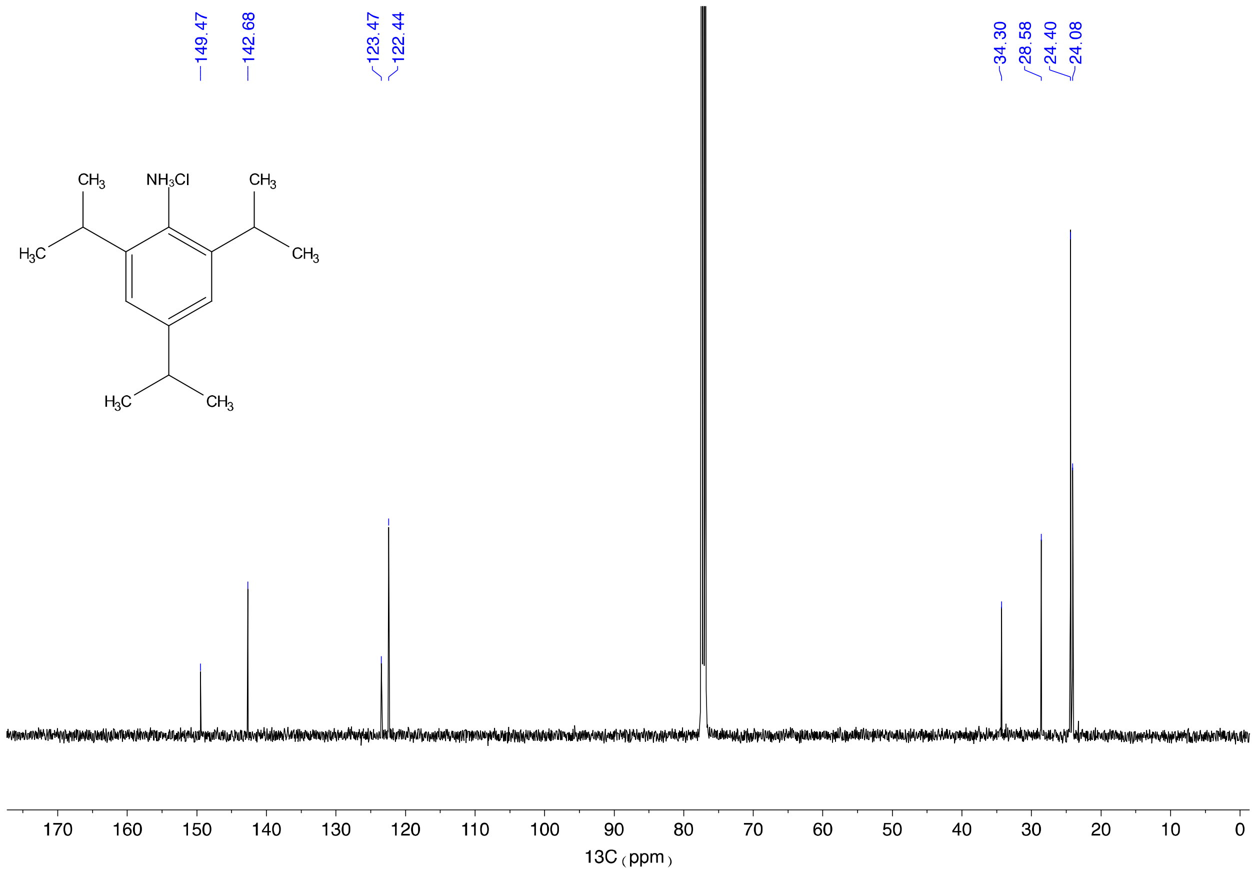

Figure S4. $^{13}C\{^{1}H\}$ NMR spectrum (125.7 MHz, $CDCl_3$, 298 K) of $TripNH_3^{+}Cl^{-}\cdot 1.75\ H_2O$, **10**$\cdot$1.75 H_2O (as prepared).

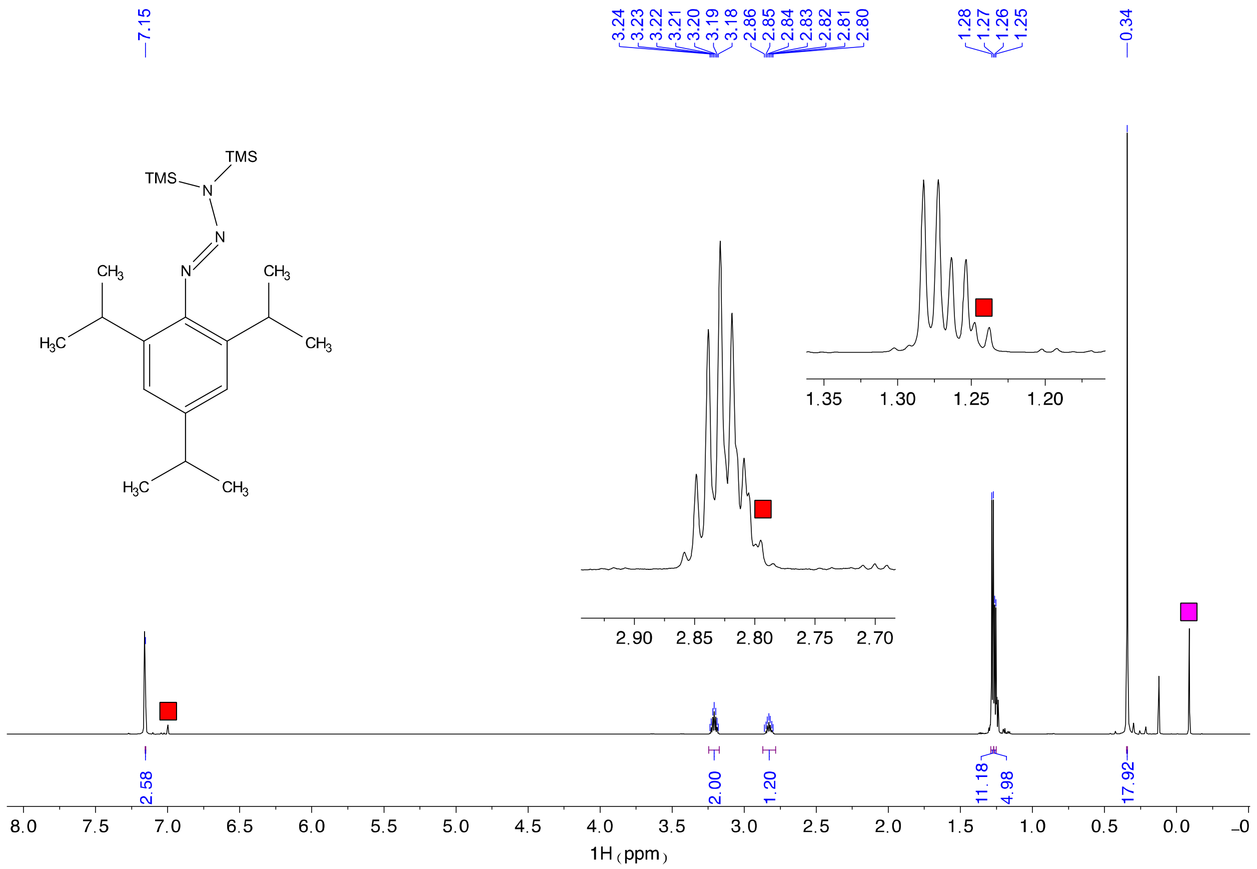

Figure S5. ^{1}H NMR spectrum (700.1 MHz, C_6D_6, 295 K) of *in-situ* prepared $TripN_2N(SiMe_3)_2$ **8**. Red squares denote traces of TripH **3** that were present in the starting material (TripLi **1**). The magenta square denotes a slight excess of unreacted Me_3SiN_3.

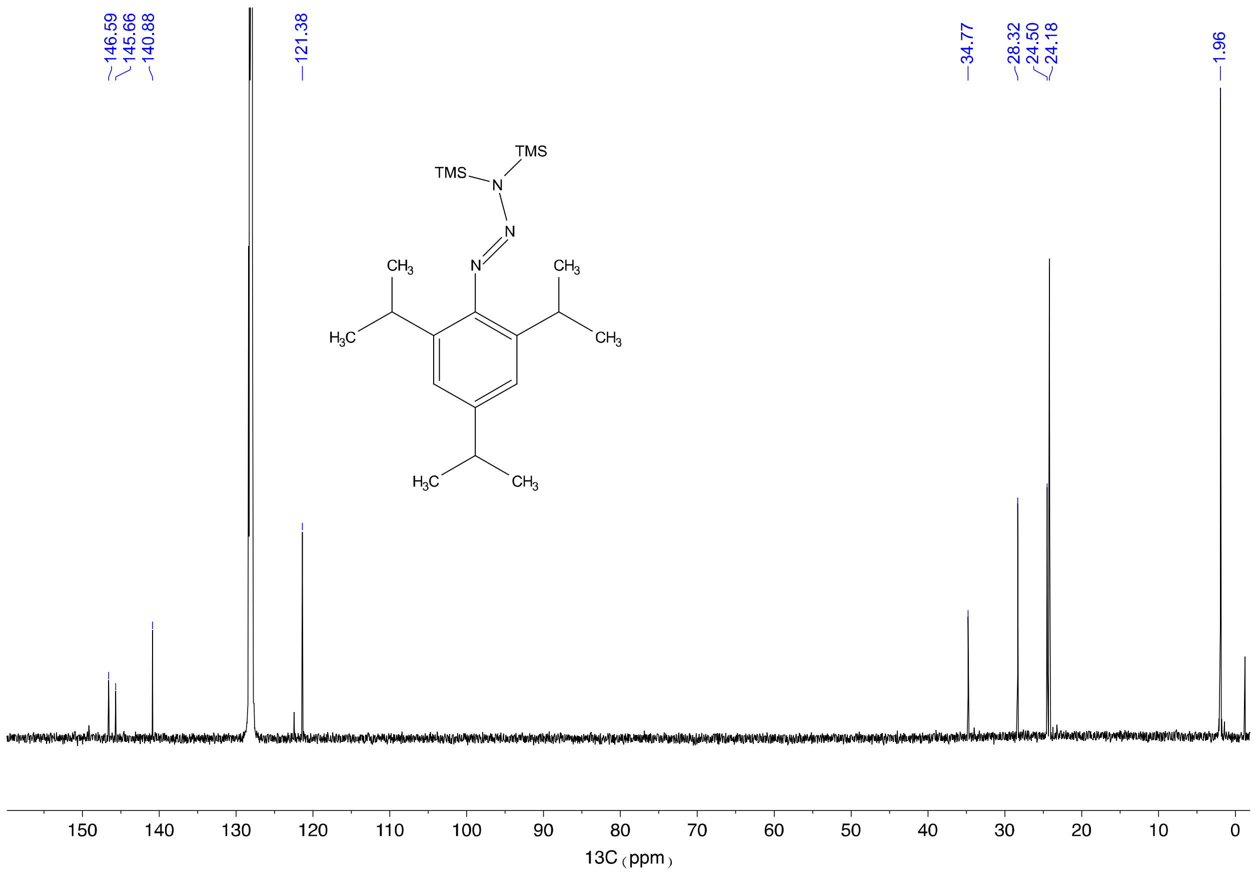

Figure S6. $^{13}C\{^{1}H\}$ NMR spectrum (176.0 MHz, C_6D_6, 295 K) of *in-situ* prepared $TripN_2N(SiMe_3)_2$ **8**.

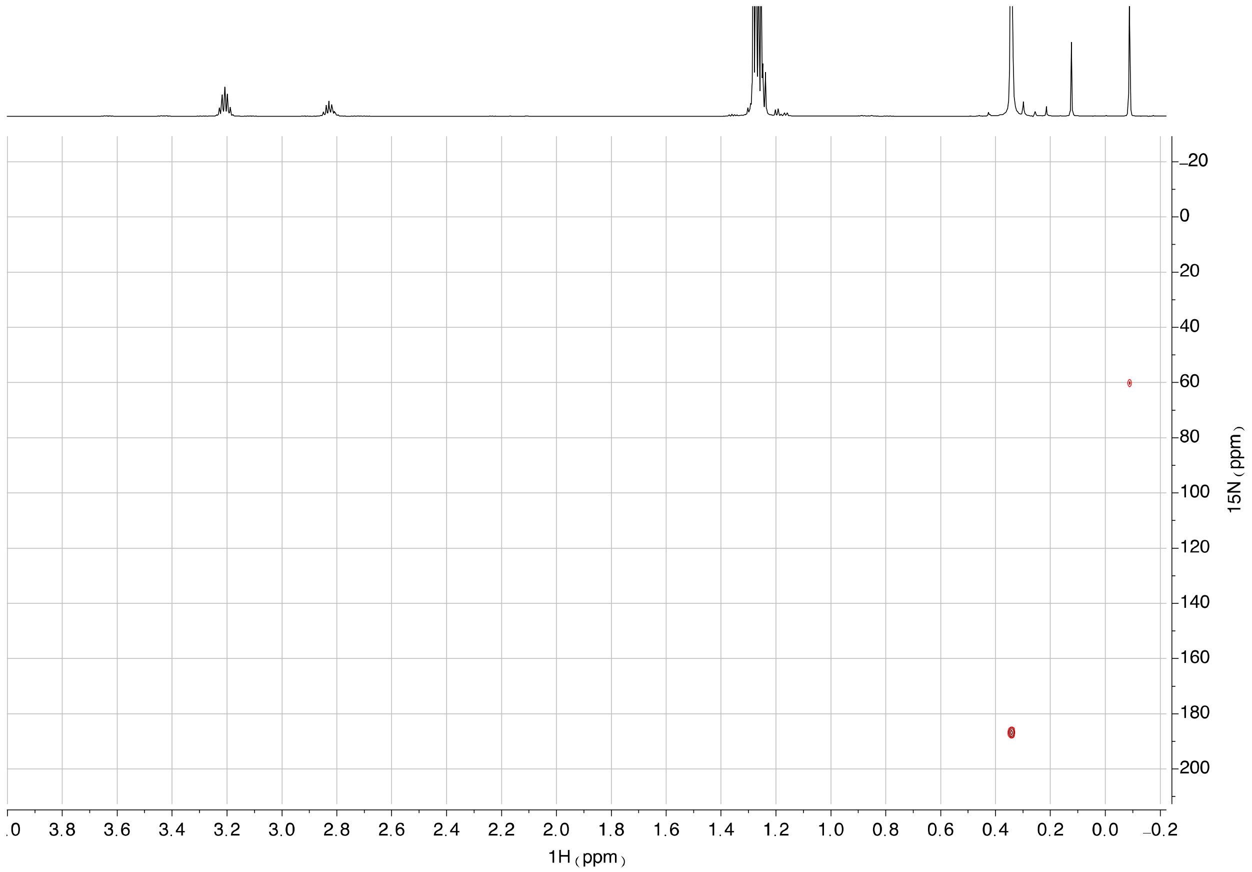

Figure S7. ^{1}H-^{15}N-gs-HMBC NMR (700.1/70.9 MHz, C_6D_6, 295 K) spectrum of *in-situ* prepared $TripN_2N(SiMe_3)_2$ **8**.

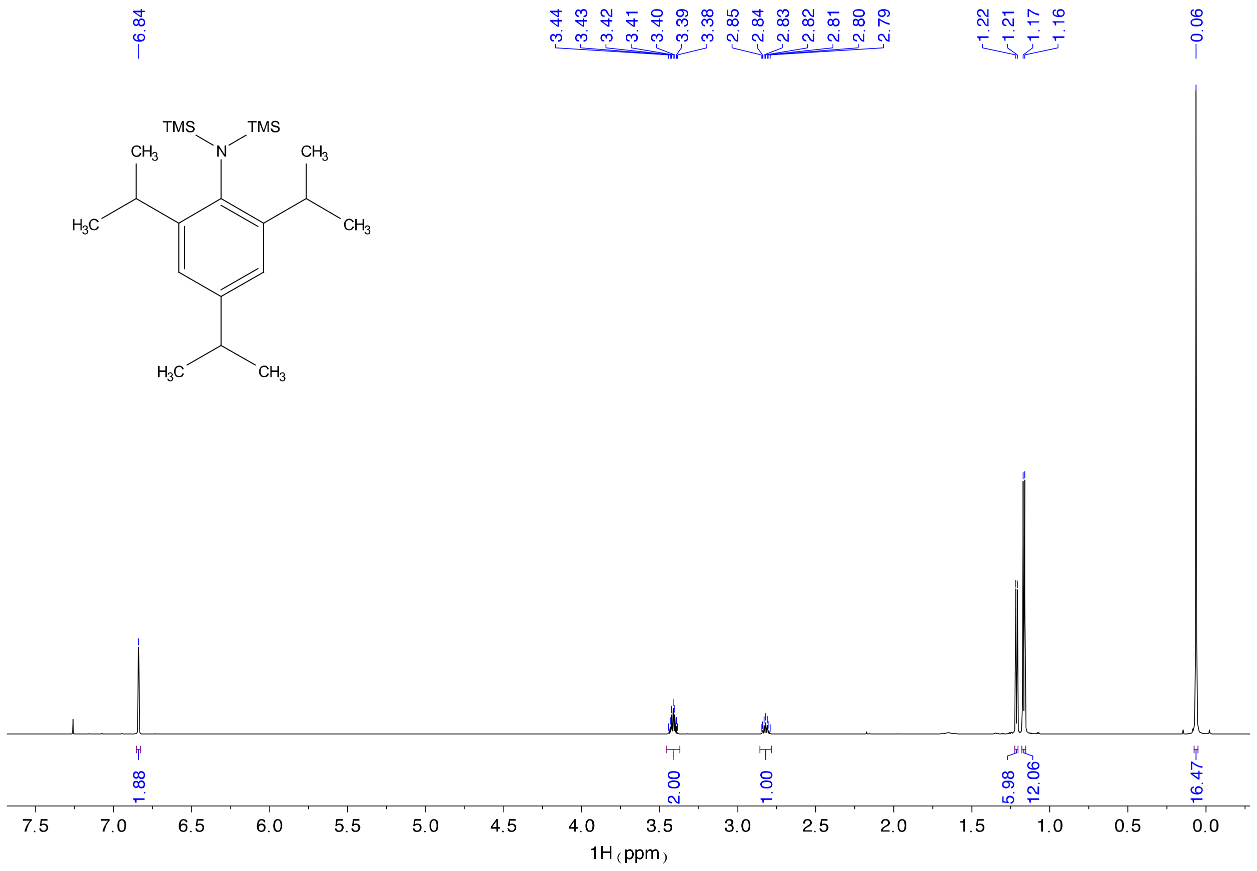

Figure S8. ^{1}H NMR spectrum (700.1 MHz, $CDCl_3$, 295 K) of $TripN(SiMe_3)_2$ **9**.

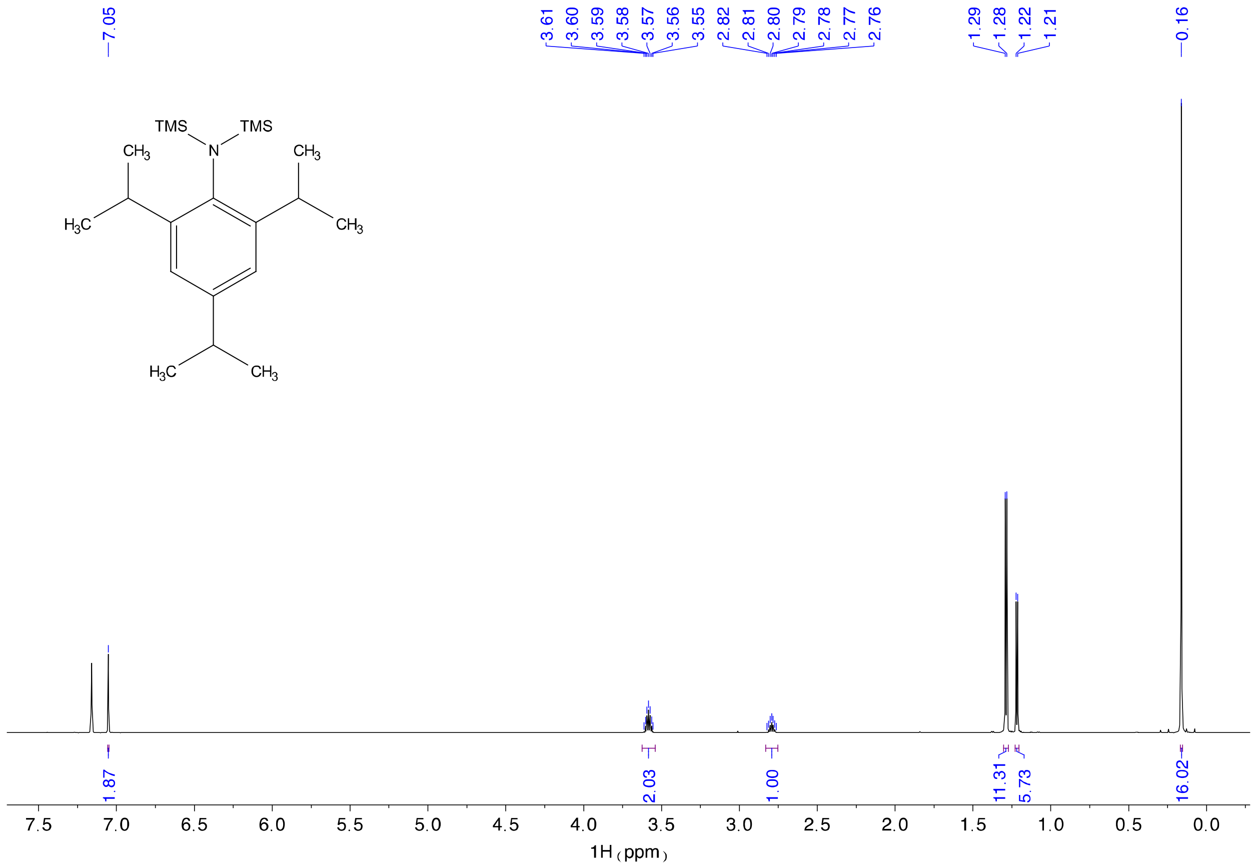

Figure S9. ^{1}H NMR spectrum (700.1 MHz, C_6D_6, 295 K) of TripN($SiMe_3$)$_2$ **9**.

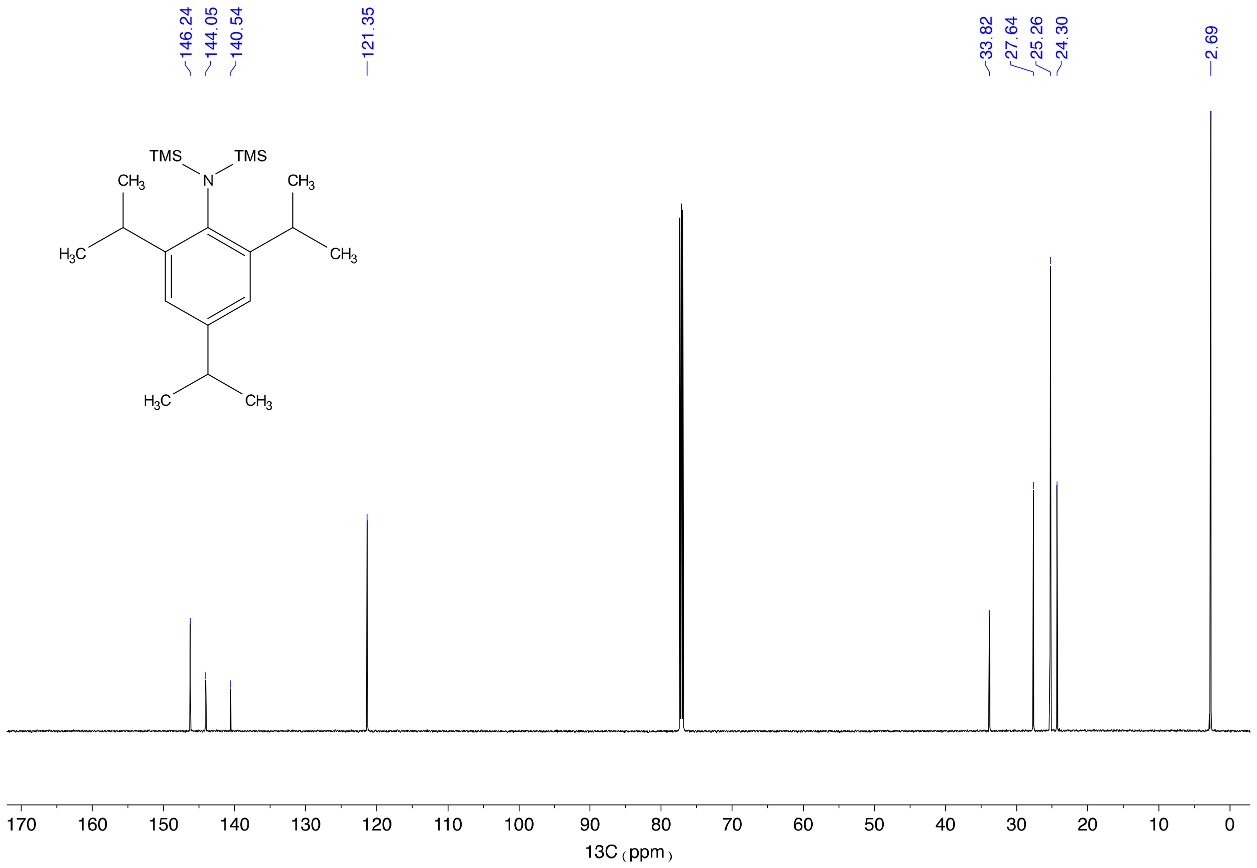

Figure S10. $^{13}C\{^1H\}$ NMR spectrum (176.0 MHz, $CDCl_3$, 295 K) of $TripN(SiMe_3)_2$ **9**.

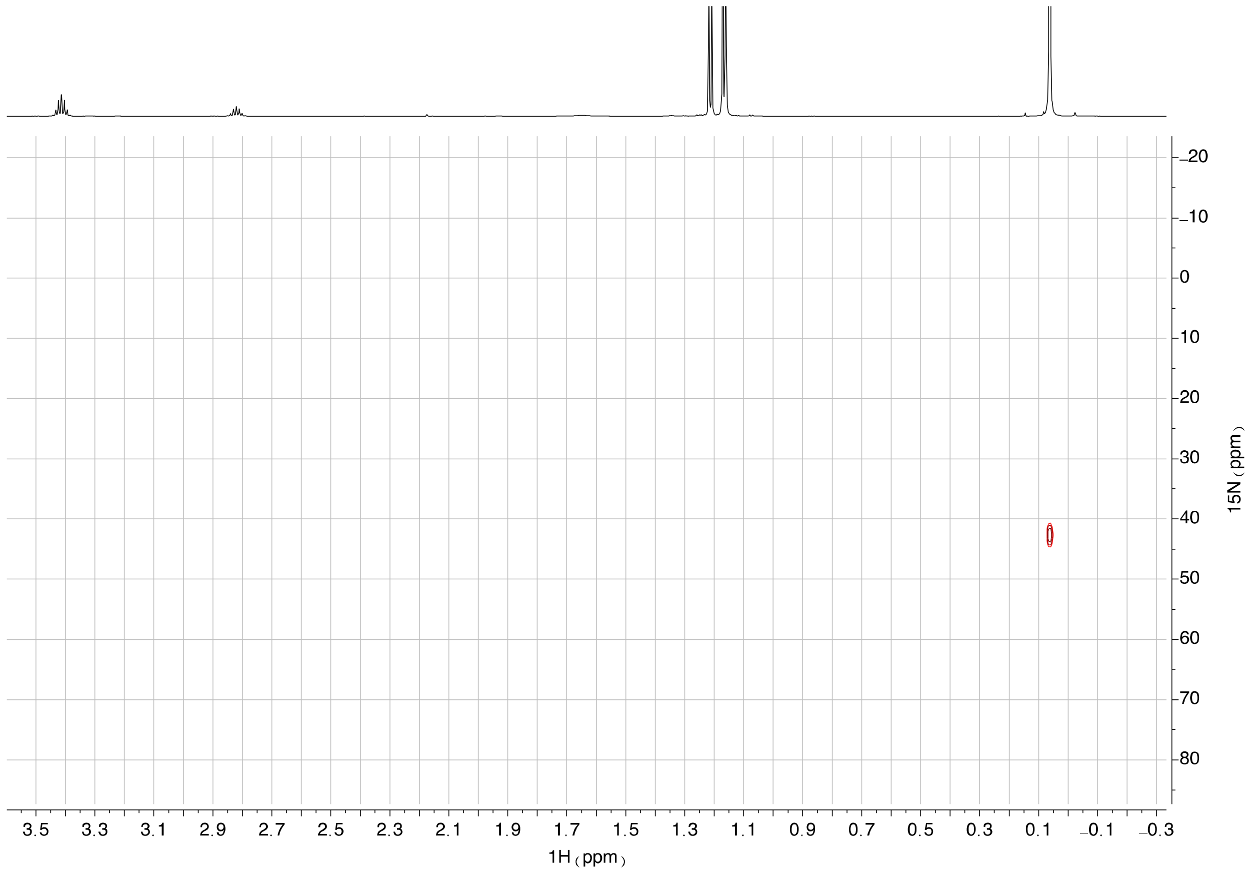

Figure S11. ^{1}H-^{15}N-gs-HMBC NMR (700.1/70.9 MHz, C_6D_6, 295 K) spectrum of TripN(SiMe$_3$)$_2$ **9**.

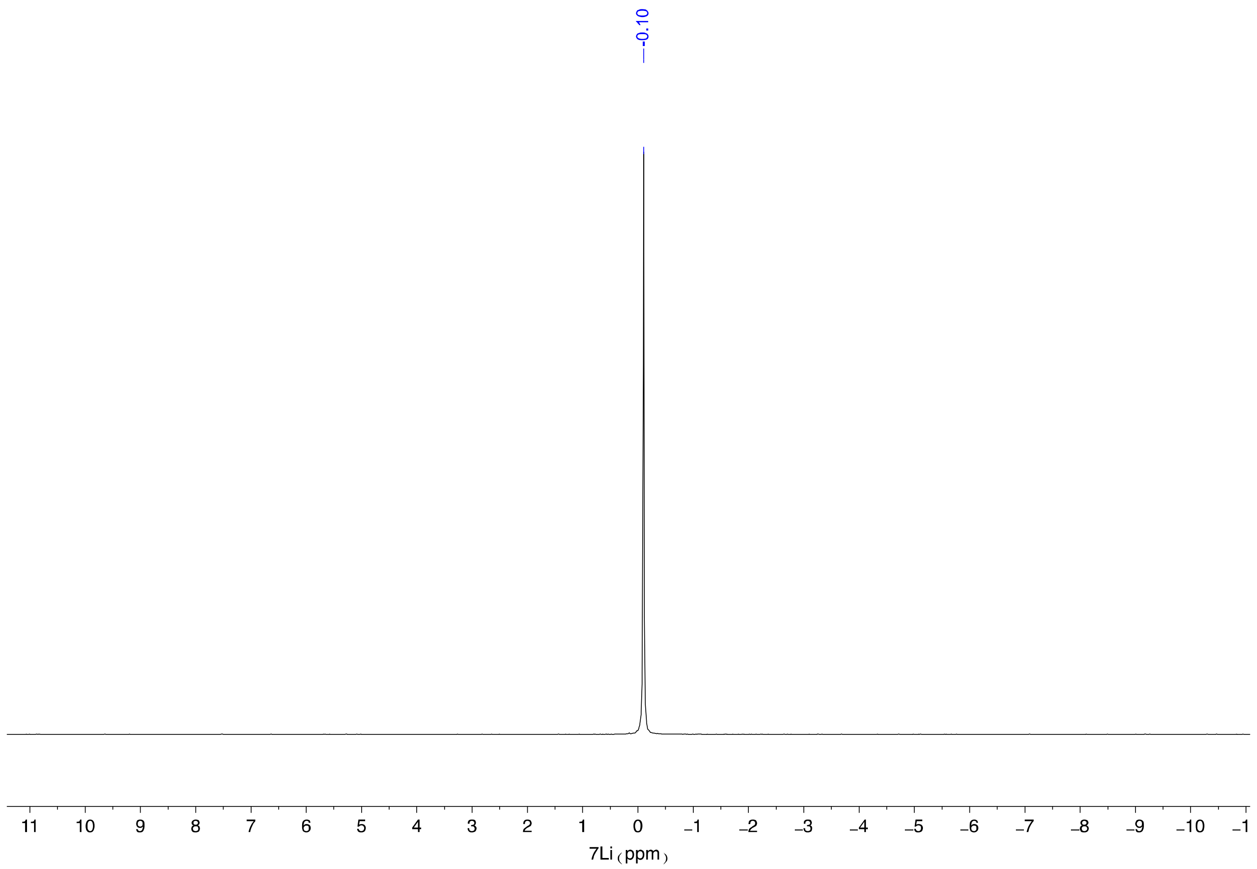

Figure S12. ^{7}Li NMR spectrum (155.5 MHz, D_2O, 294 K) of the LiN_3 by-product.

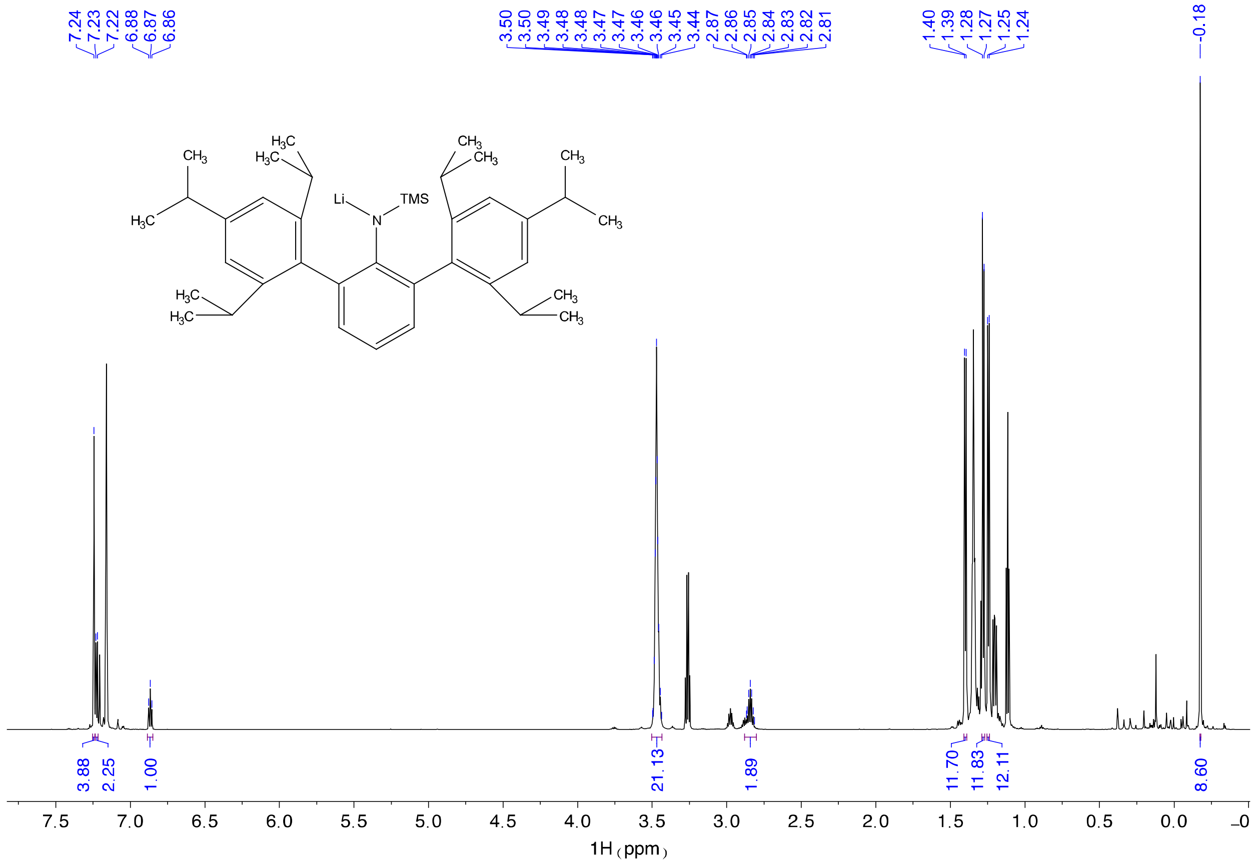

Figure S13. ^{1}H NMR spectrum (700.1 MHz, C_6D_6, 295 K) of *in-situ* prepared TerN($SiMe_3$)Li **14**. The sample also contains diethyl ether, THF and TerH **13**. The resonances marked in blue belong to **14**.

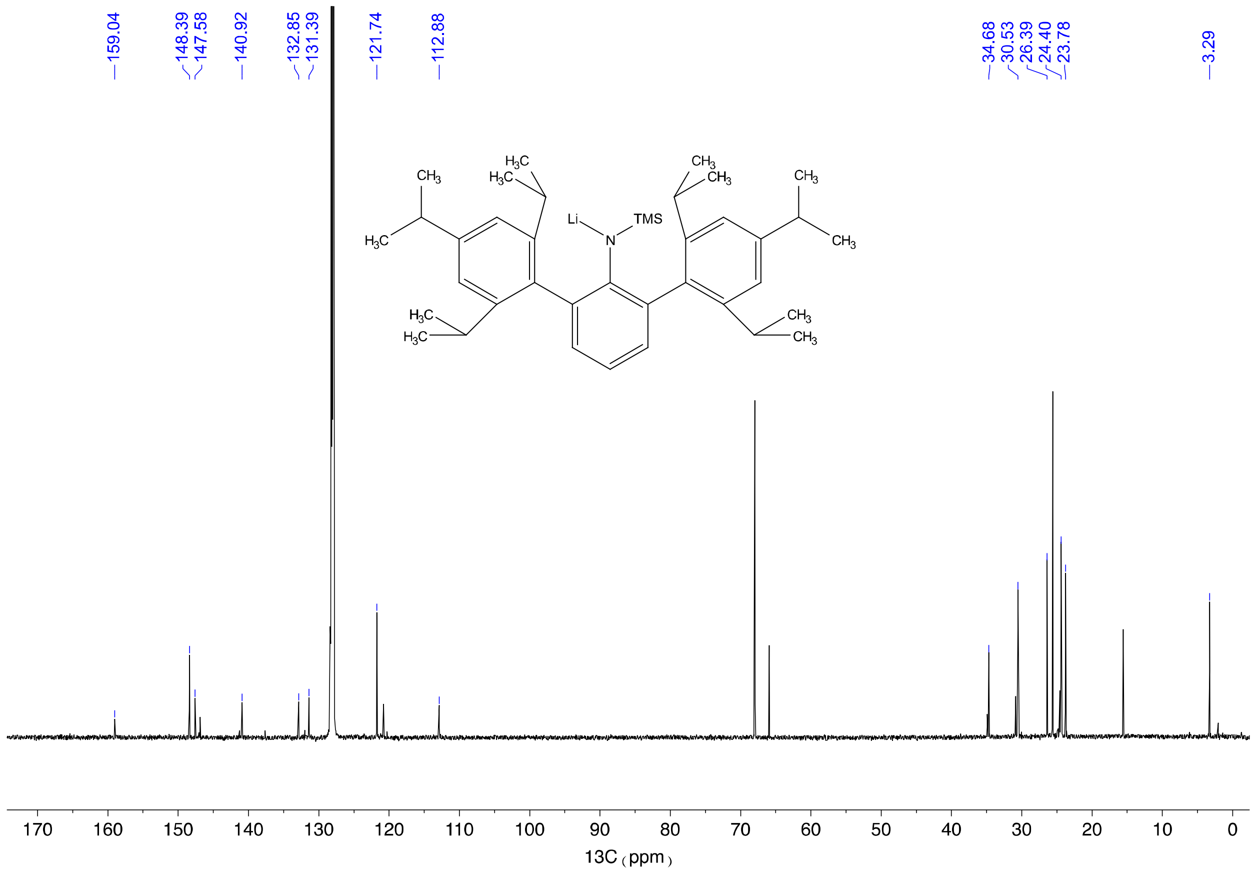

Figure S14. $^{13}C\{^1H\}$ NMR spectrum (176.0 MHz, C_6D_6, 295 K) of *in-situ* prepared TerN(SiMe$_3$)Li **14**. The sample also contains diethyl ether, THF and TerH **13**. The resonances marked in blue belong to **14**.

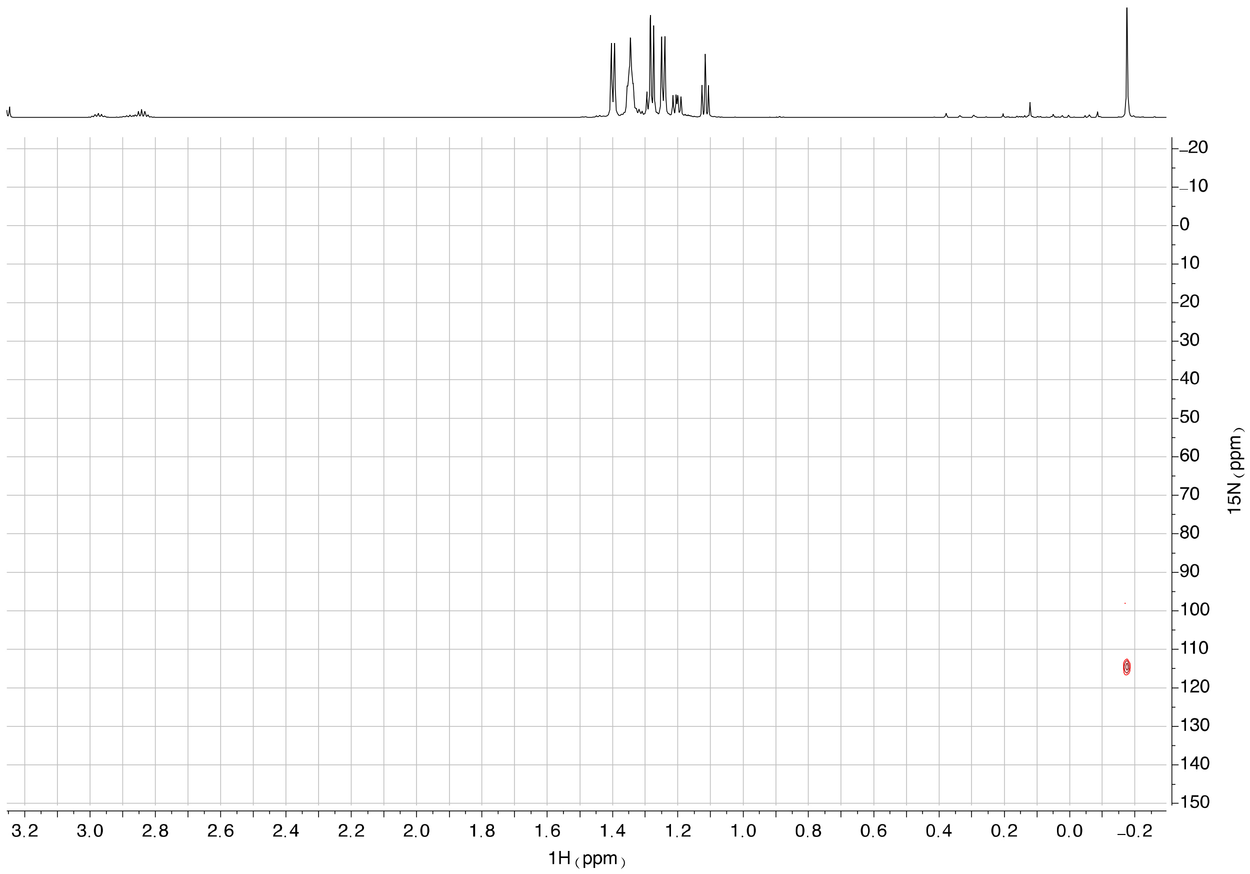

Figure S15. ^{1}H-^{15}N-gs-HMBC NMR (700.1/70.9 MHz, C_6D_6, 295 K) spectrum of *in-situ* prepared TerN($SiMe_3$)Li **14**.

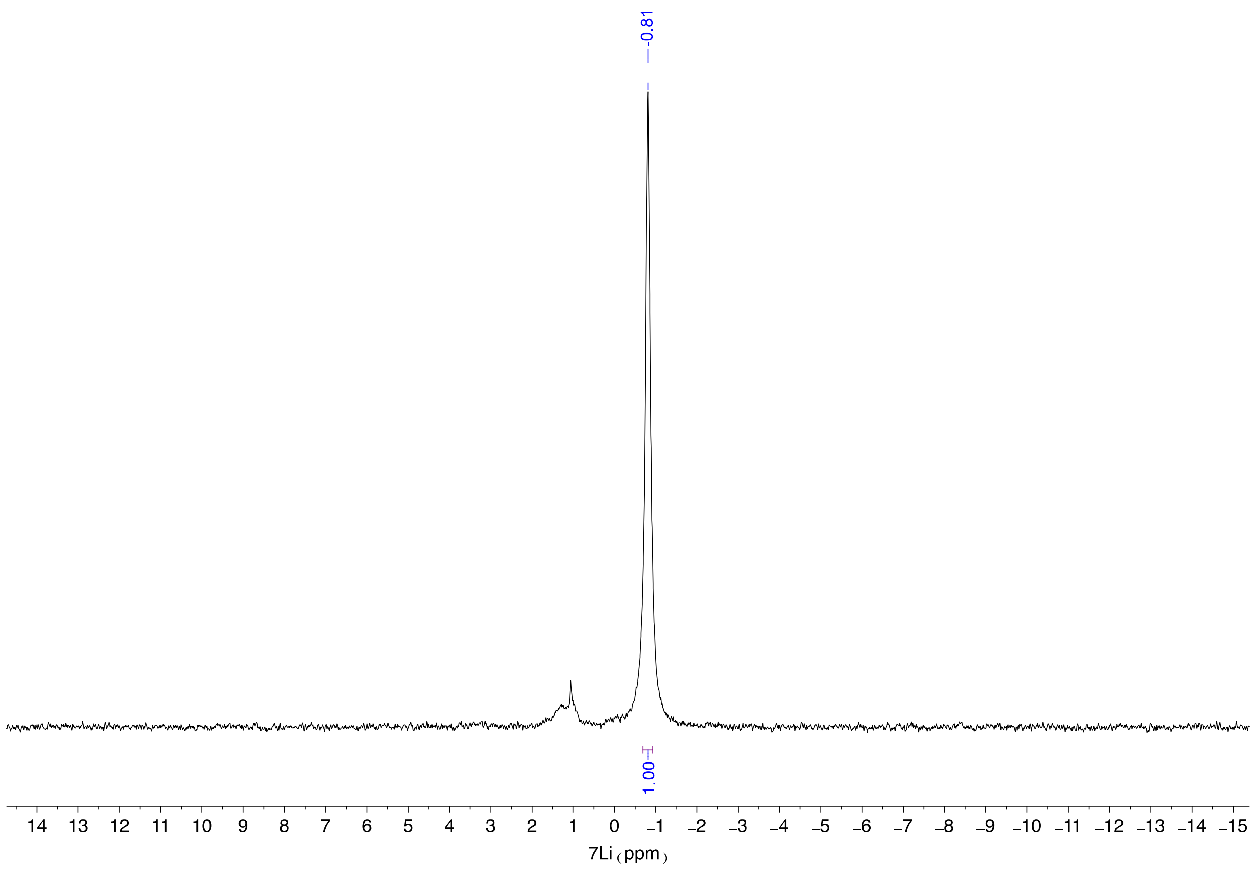

Figure S16. ^{7}Li NMR spectrum (155.5 MHz, C_6D_6, 295 K) of *in-situ* prepared TerN($SiMe_3$)Li **14**.

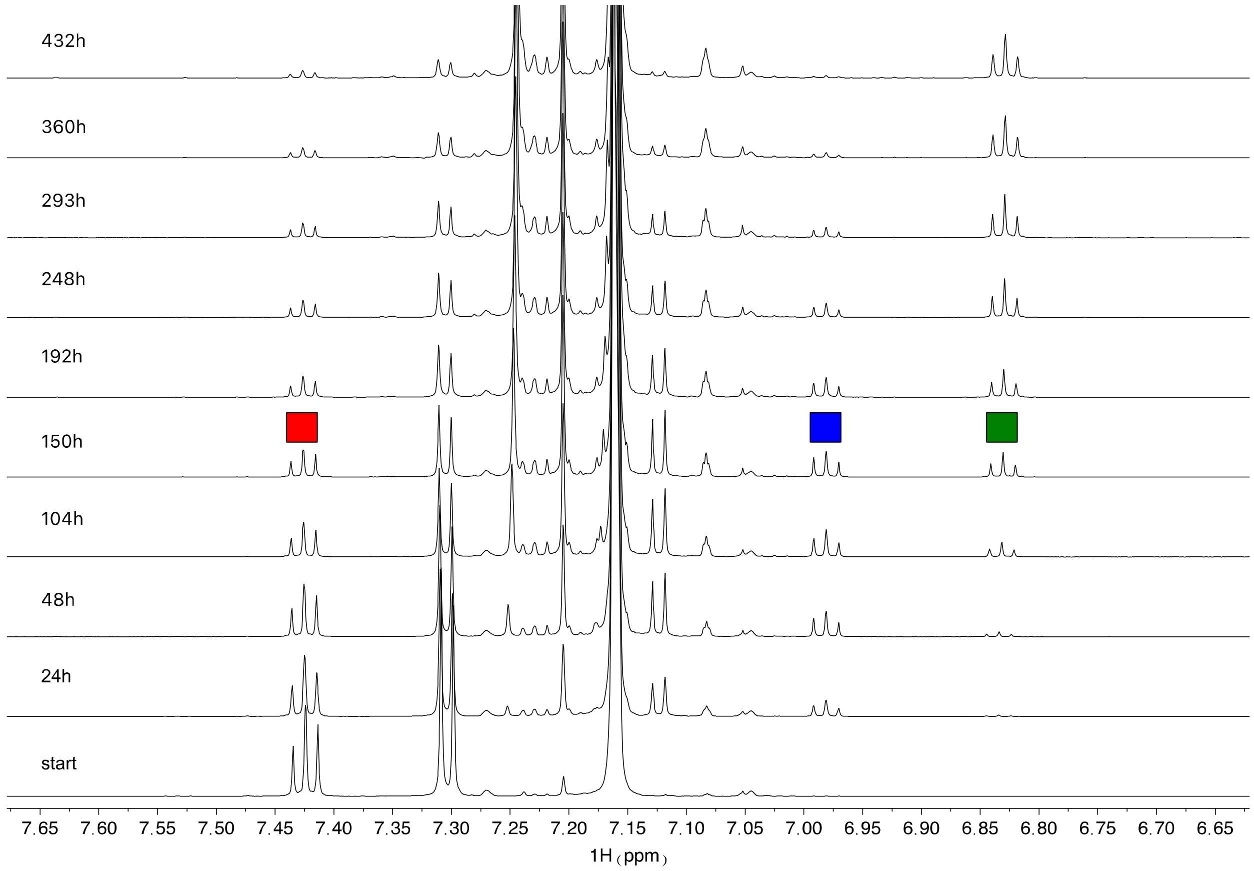

Figure S17. Stacked ^{1}H NMR spectra (700.1 MHz, C_6D_6, 295 K) for the reaction of TerLi(OEt_2) **11**(OEt_2) with Me_3SiN_3 in C_6D_6 with additional Et_2O (5 eq.) added at room temperature. The aromatic chemical shift region has been chosen for best visualisation. The red square denotes starting **11**(OEt_2), the blue square denotes the proposed intermediate $TerN_2N(SiMe_3)Li$ **15**, and the green square denotes the product $TerN(SiMe_3)Li$ **14** before workup.

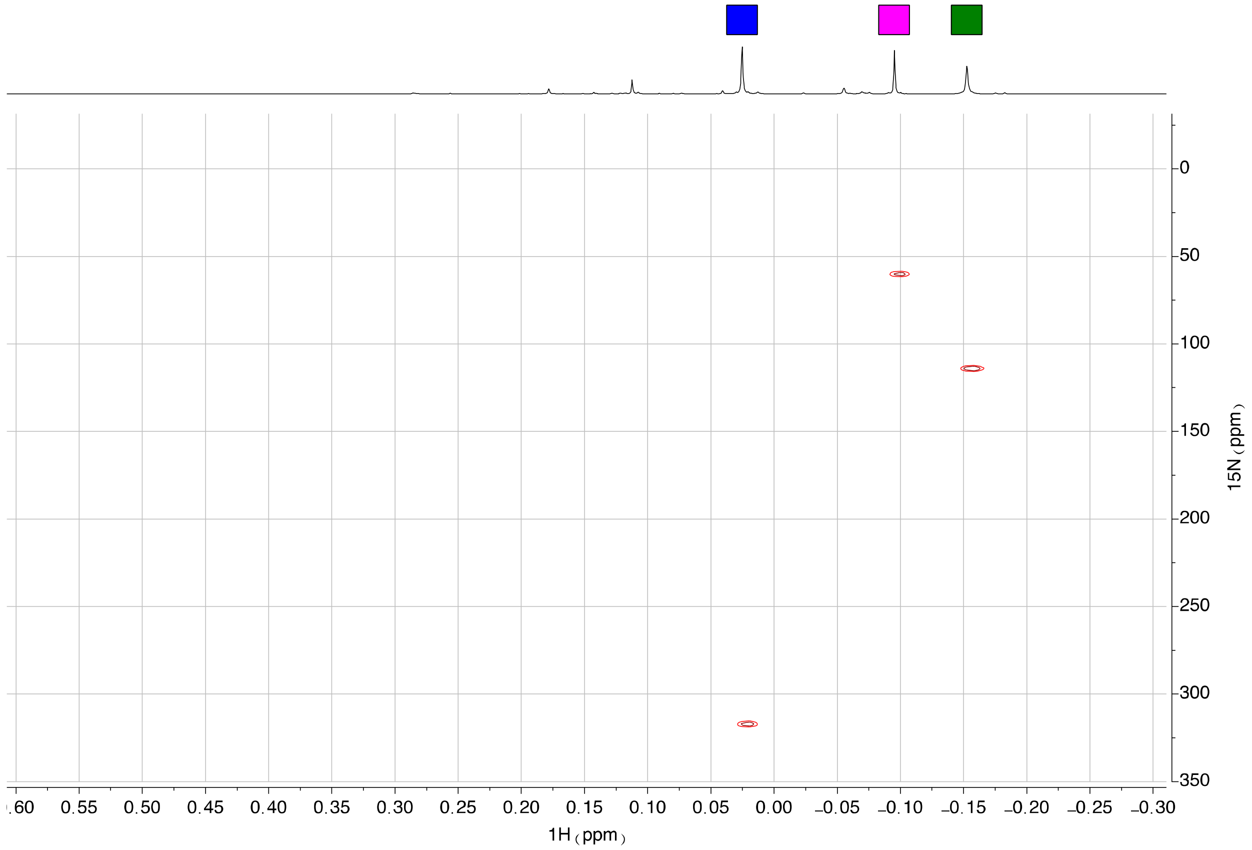

Figure S18. ^{1}H-^{15}N-gs-HMBC NMR (700.1/70.9 MHz, C_6D_6, 295 K) spectrum of the reaction between TerLi(OEt_2) **11**(OEt_2) and Me_3SiN_3 in C_6D_6 with additional Et_2O (5 eq.) added at room temperature after 150 h. The magenta square denotes Me_3SiN_3, the blue square denotes the proposed intermediate $TerN_2N(SiMe_3)Li$ **15**, and the green square denotes the product $TerN(SiMe_3)Li$ **14**.

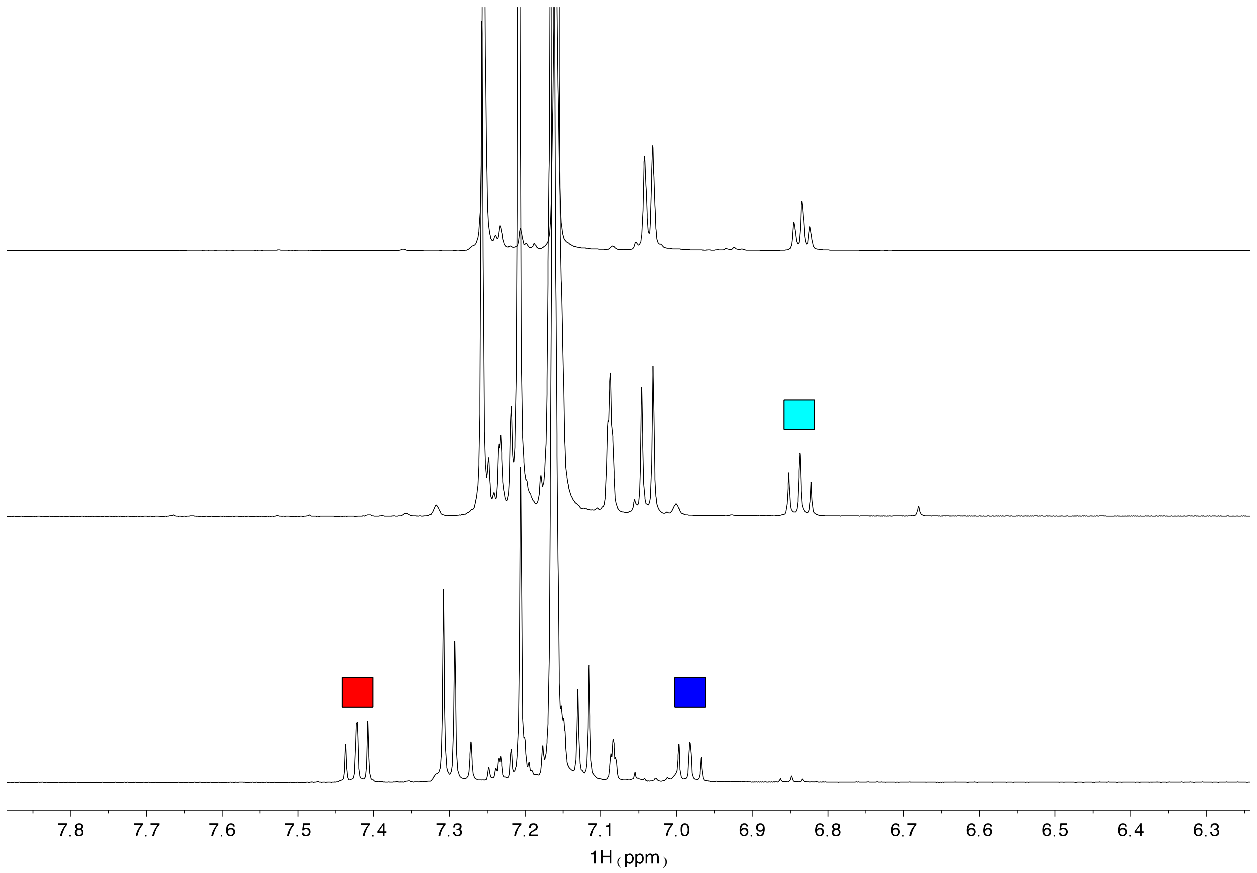

Figure S19. Stacked ^{1}H NMR spectra (various field strengths, aromatic chemical shift region only) of the workup of the proposed *in-situ* generated intermediate $TerN_2N(SiMe_3)Li$ **15** to $TerNH_2$ **17**. Bottom: The ^{1}H NMR spectrum depicts the reaction between $TerLi(OEt_2)$ **11**(OEt_2) and Me_3SiN_3 in C_6D_6 with additional Et_2O (5 eq.) after 21 h at room temperature prior to quenching. The red square denotes unreacted starting material $TerLi(OEt_2)$ **11**(OEt_2) and the blue square denotes the observed intermediate, proposed to be $TerN_2N(SiMe_3)Li$ **15**. Middle: ^{1}H NMR spectrum after the mixture of **11** and **15** was quenched with methanol (which involved the addition of *ca.* 3 mL MeOH, removal of all volatiles, drying *in vacuo* and redissolving the residue in C_6D_6). The cyan square denotes the aniline $TerNH_2$ **17**. Top: ^{1}H NMR spectrum of $TerNH_2$ **17** obtained from deprotection of $TerN(SiMe_3)H$ **16** with silica.

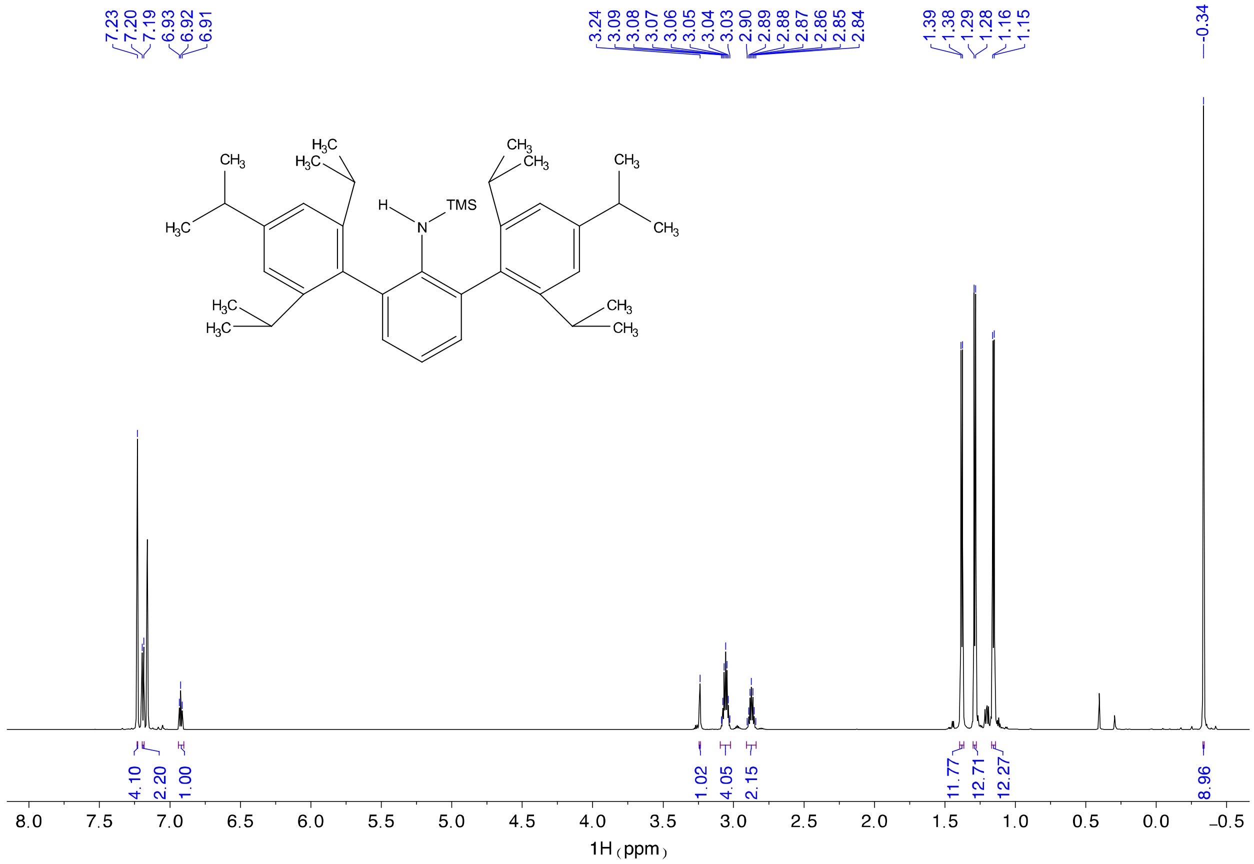

Figure S20. ^{1}H NMR spectrum (700.1 MHz, C_6D_6, 295 K) of TerN($SiMe_3$)H **16**.

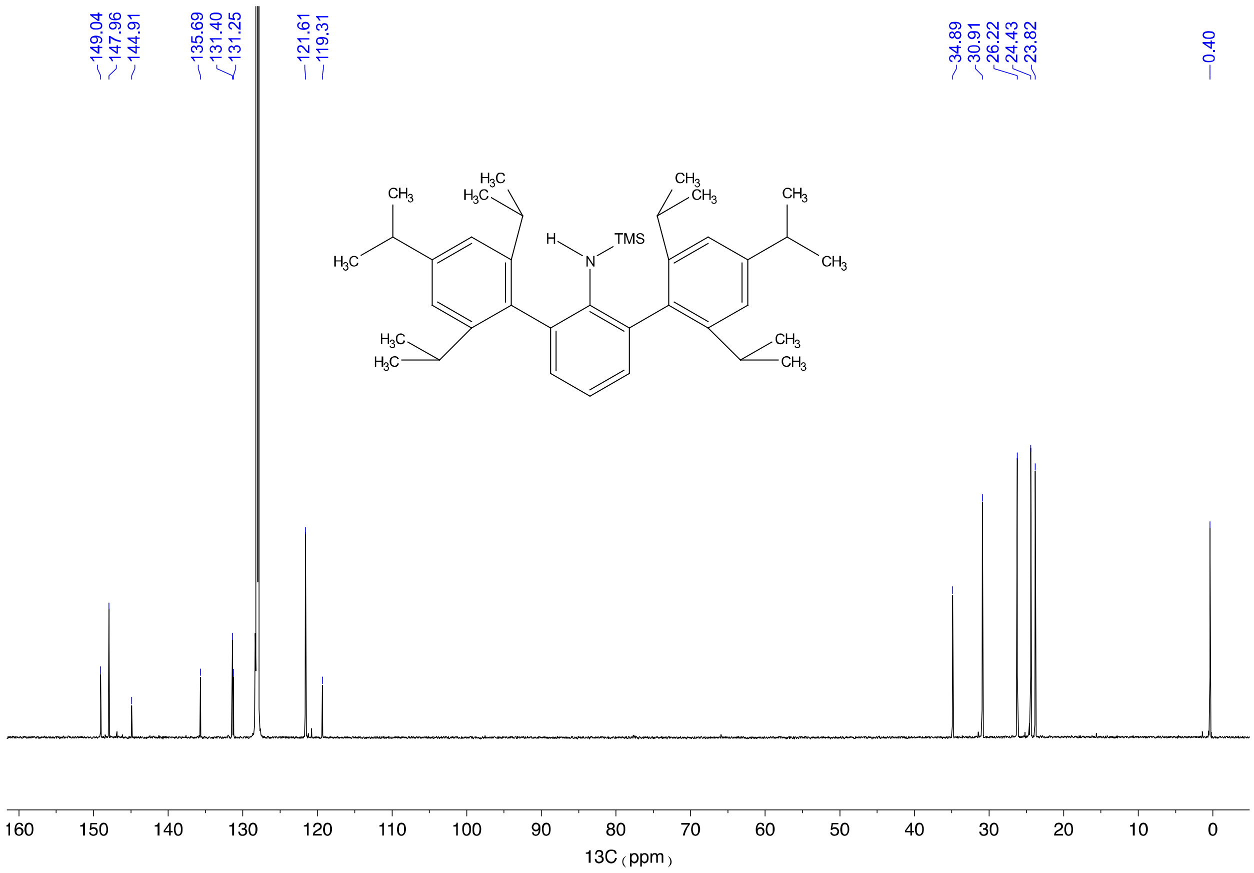

Figure S21. $^{13}C\{^{1}H\}$ NMR spectrum (176.0 MHz, C_6D_6, 295 K) of TerN($SiMe_3$)H **16**.

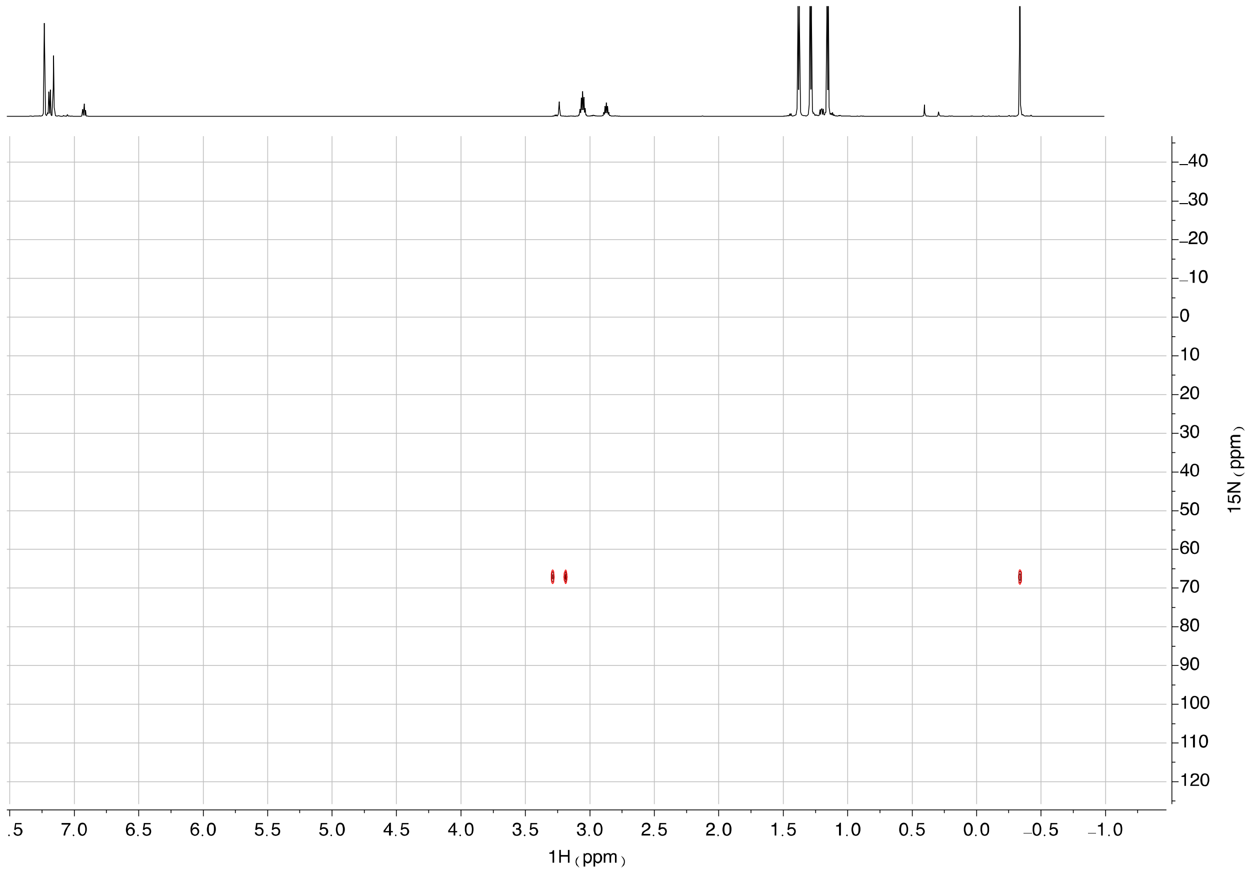

Figure S22. ^{1}H-^{15}N-gs-HMBC NMR (700.1/70.9 MHz, C_6D_6, 295 K) spectrum of TerN($SiMe_3$)H **16**.

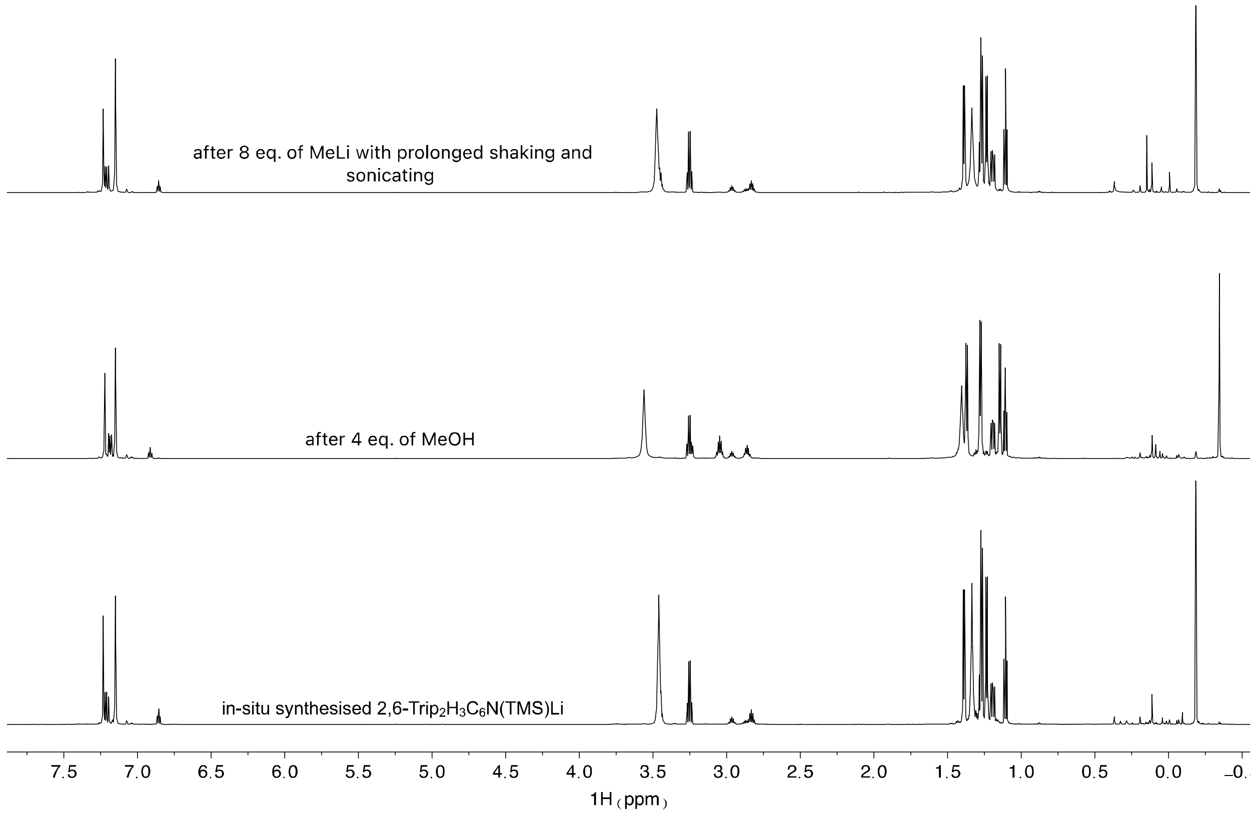

Figure S23. ^{1}H NMR spectra (700.1 MHz, C_6D_6, 295 K) showing the reaction between *in-situ* prepared TerN($SiMe_3$)Li **14** (bottom), after *in-situ* "workup" with methanol to TerN($SiMe_3$)H **16** (middle), followed by regeneration of TerN($SiMe_3$)Li **14** after reaction with an excess of solid MeLi (top).

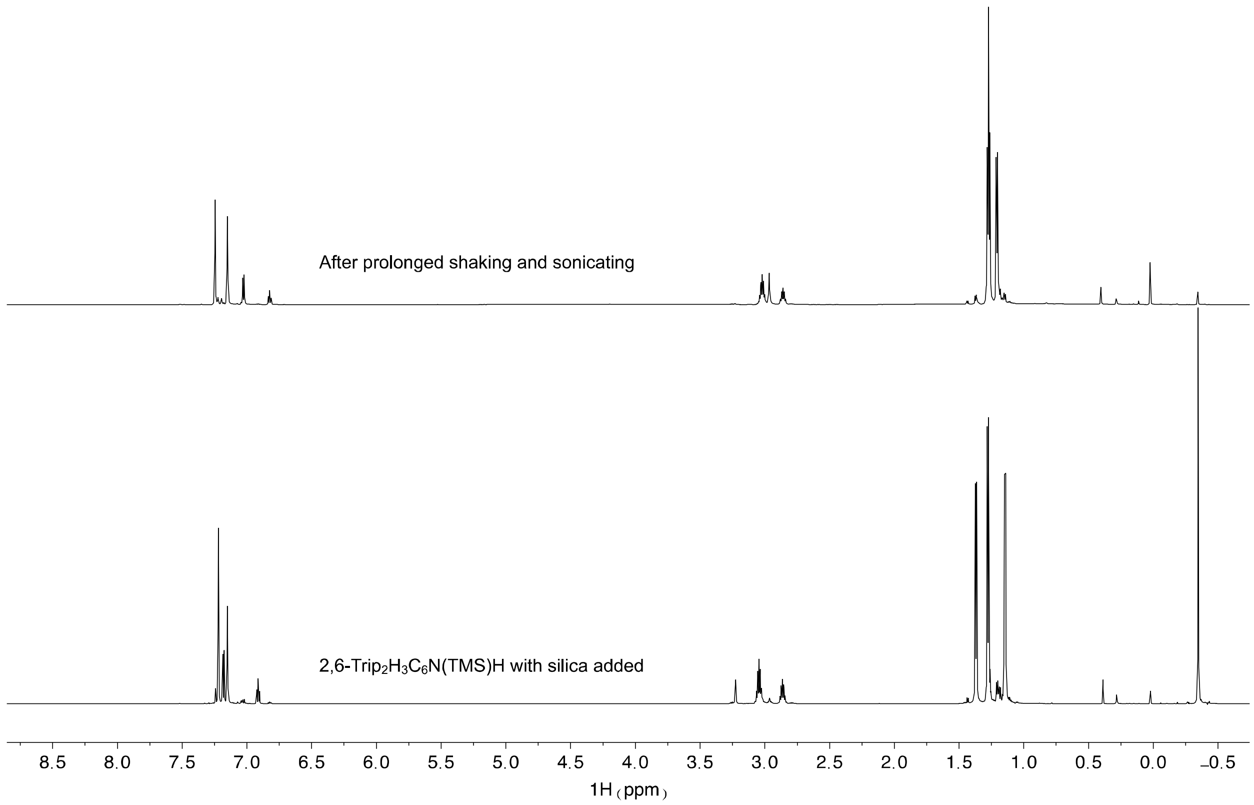

Figure S24. Deprotection of TerN(SiMe$_3$)H **16** with silica to give the corresponding aniline TerNH$_2$ **17** as monitored by ^{1}H NMR spectroscopy (700.1 MHz, C$_6$D$_6$, 295 K).

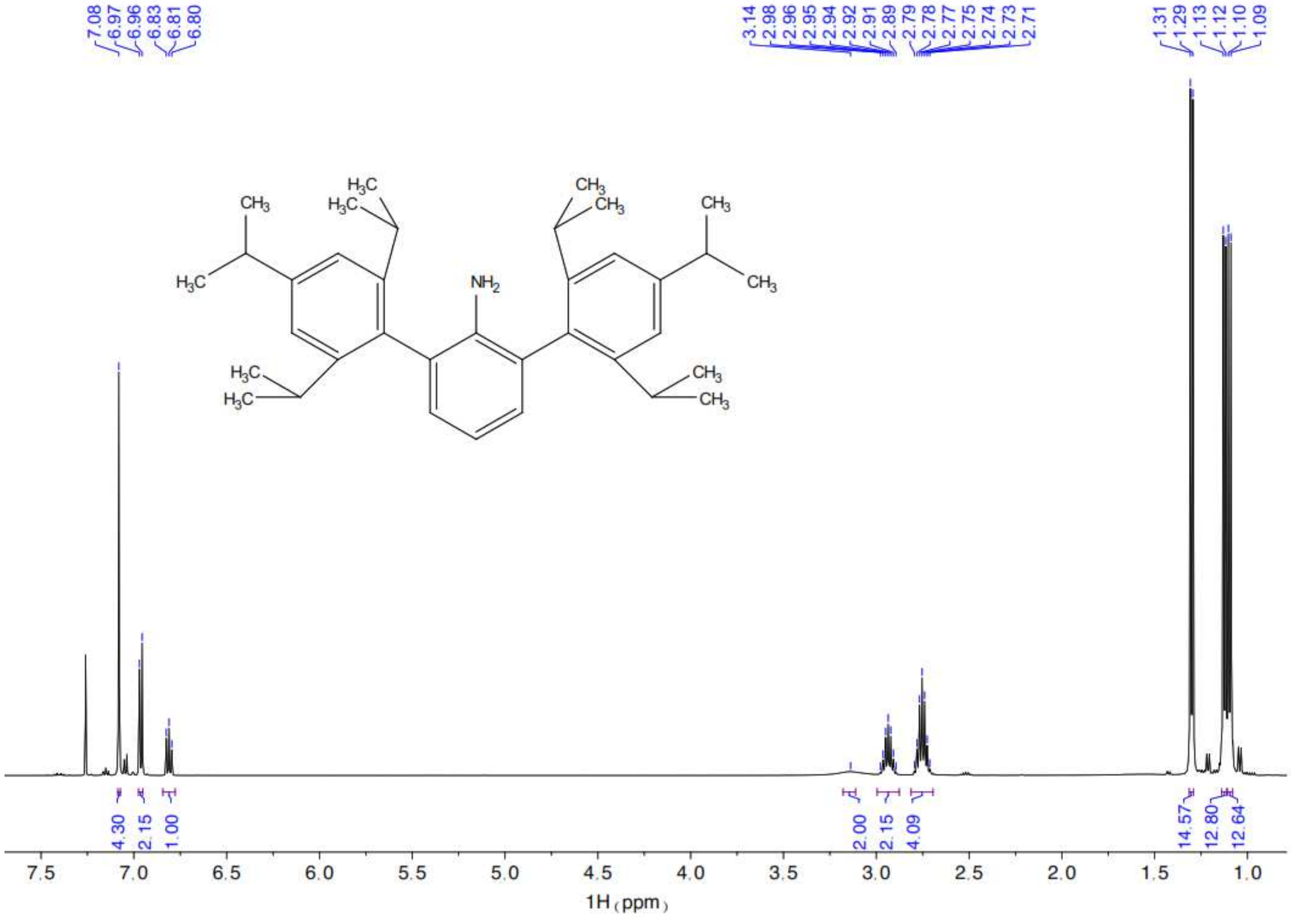

Figure S25. ^{1}H NMR spectrum (500.1 MHz, $CDCl_3$, 295 K) of $TerNH_2$ **17**.

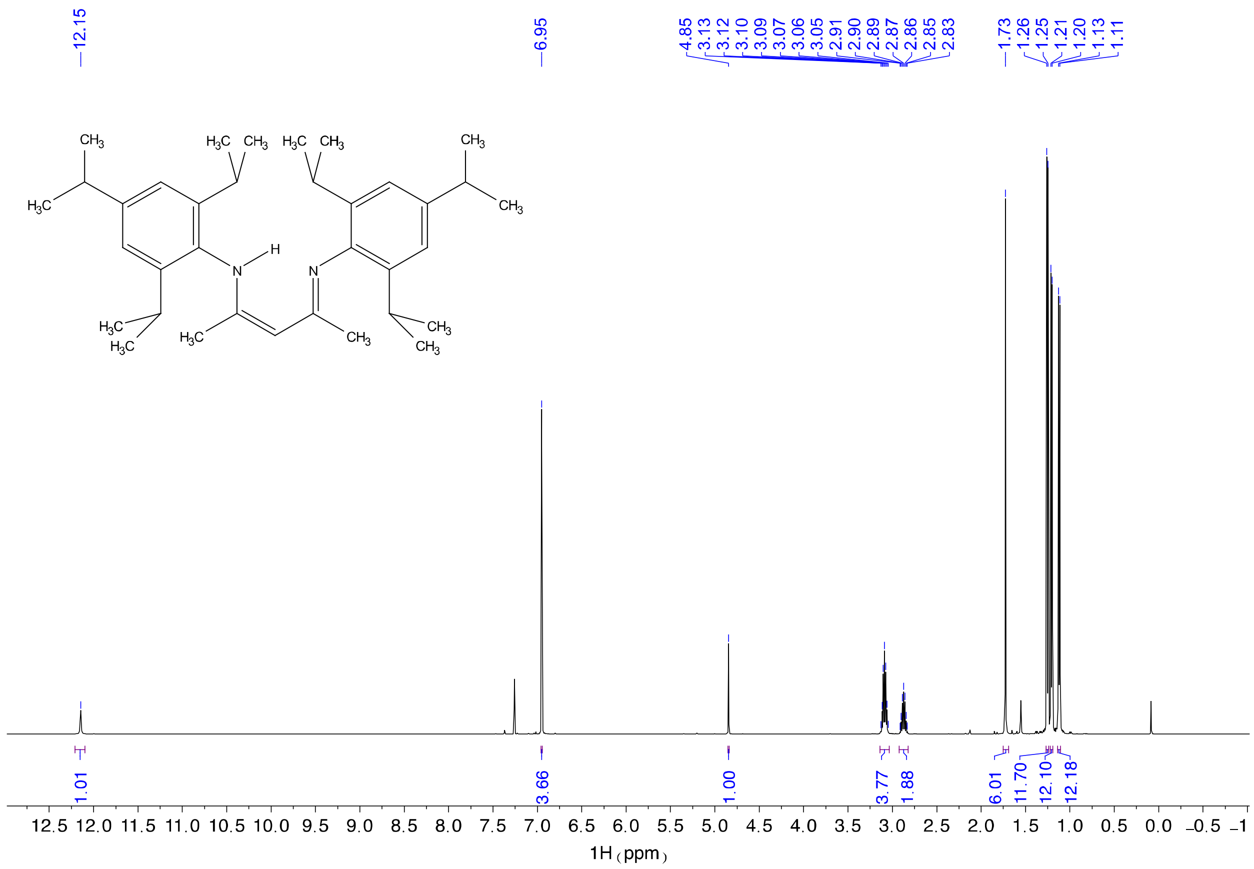

Figure S26. ^{1}H NMR spectrum (500.1 MHz, $CDCl_3$, 295 K) of ^{MeTrip}nacnacH **18**.

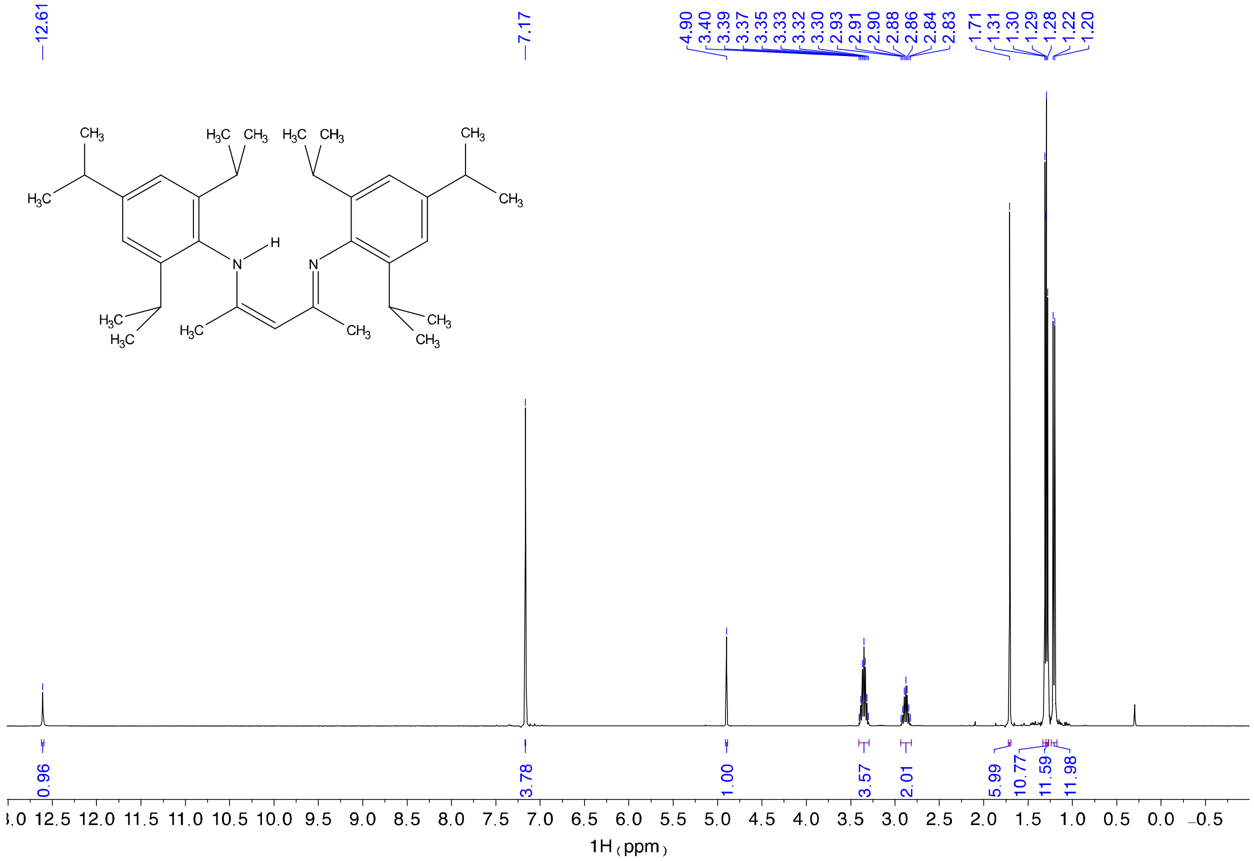

Figure S27. ^{1}H NMR spectrum (400.1 MHz, C_6D_6, 294 K) of ^{MeTrip}nacnacH **18**.

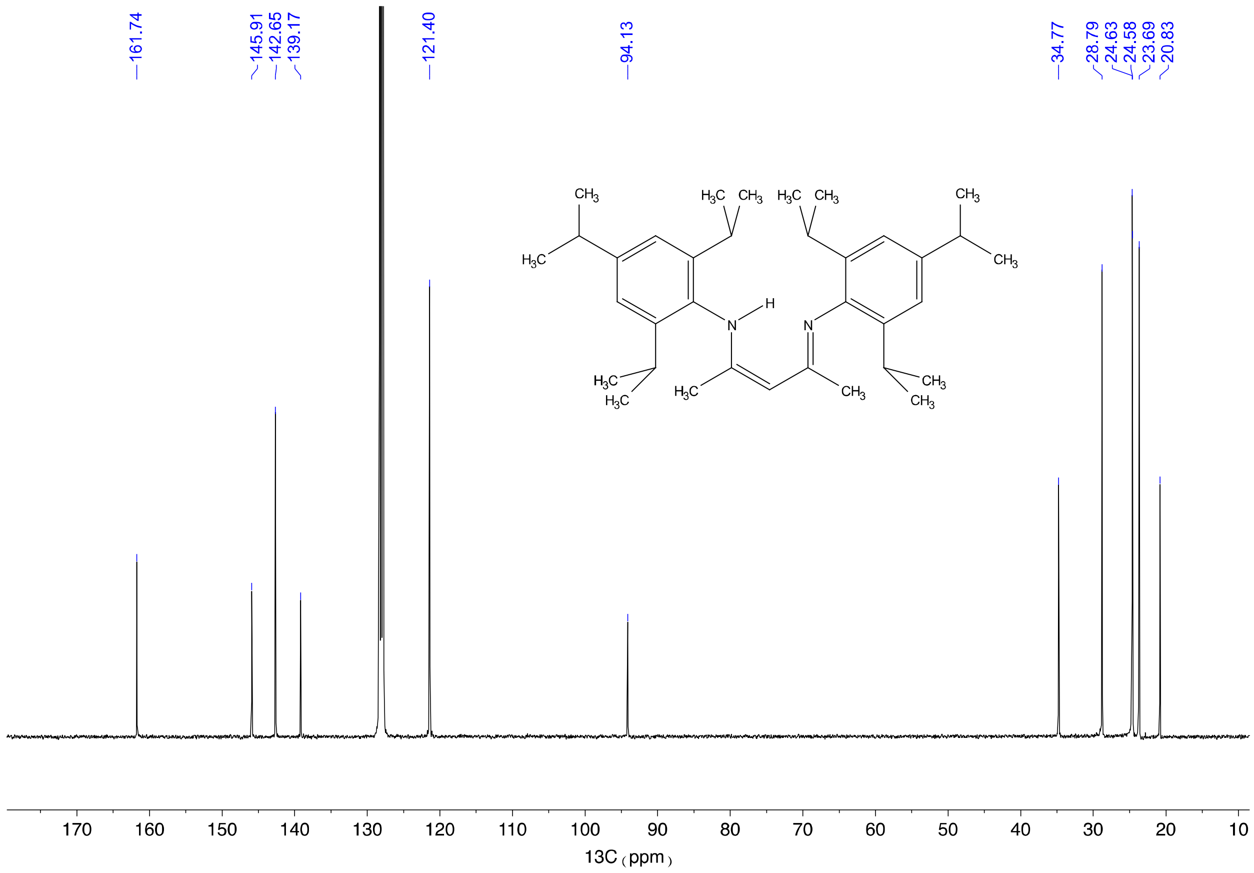

Figure S28. $^{13}C\{^{1}H\}$ NMR spectrum (100.6 MHz, C_6D_6, 295 K) of ^{MeTrip}nacnacH **18**.

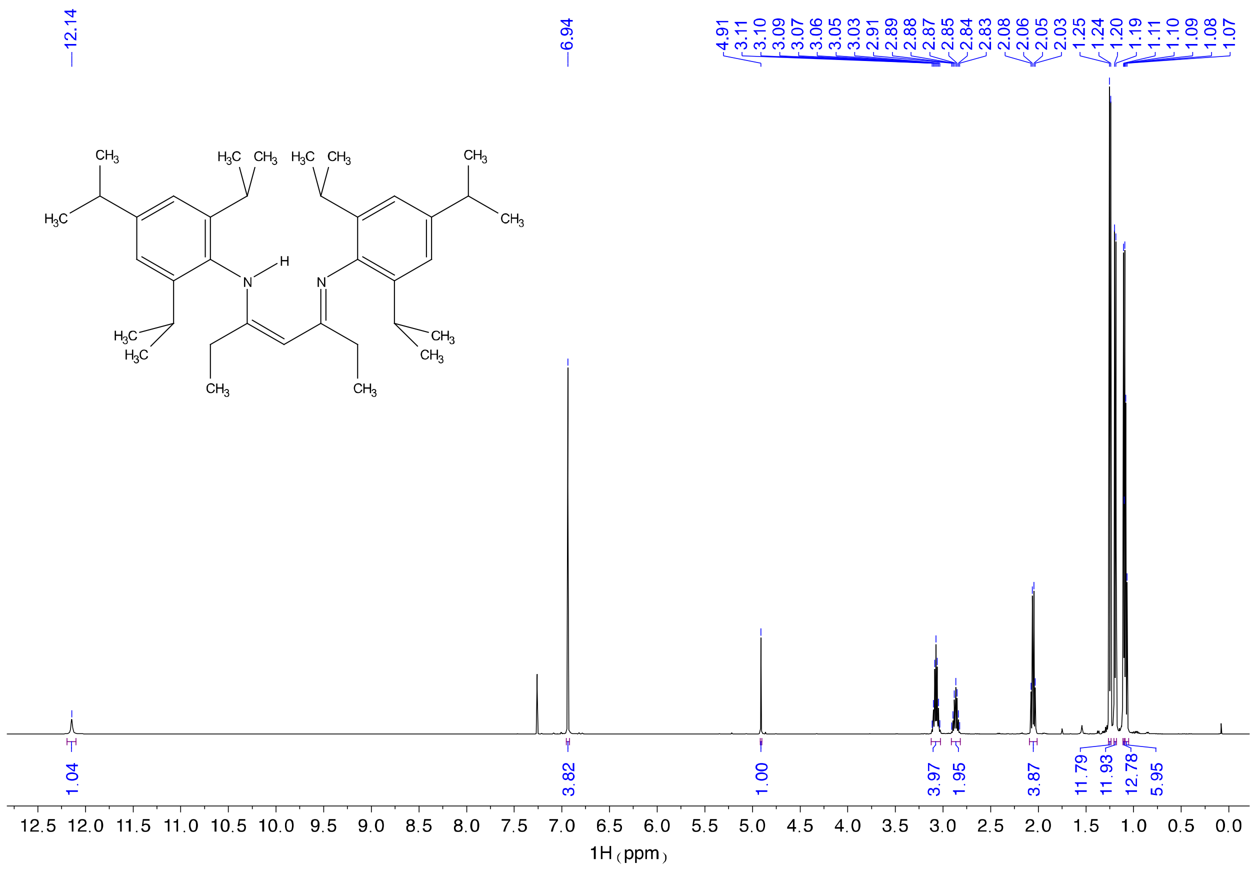

Figure S29. ^{1}H NMR spectrum (500.1 MHz, $CDCl_3$, 295 K) of ^{EtTrip}nacnacH **19**.

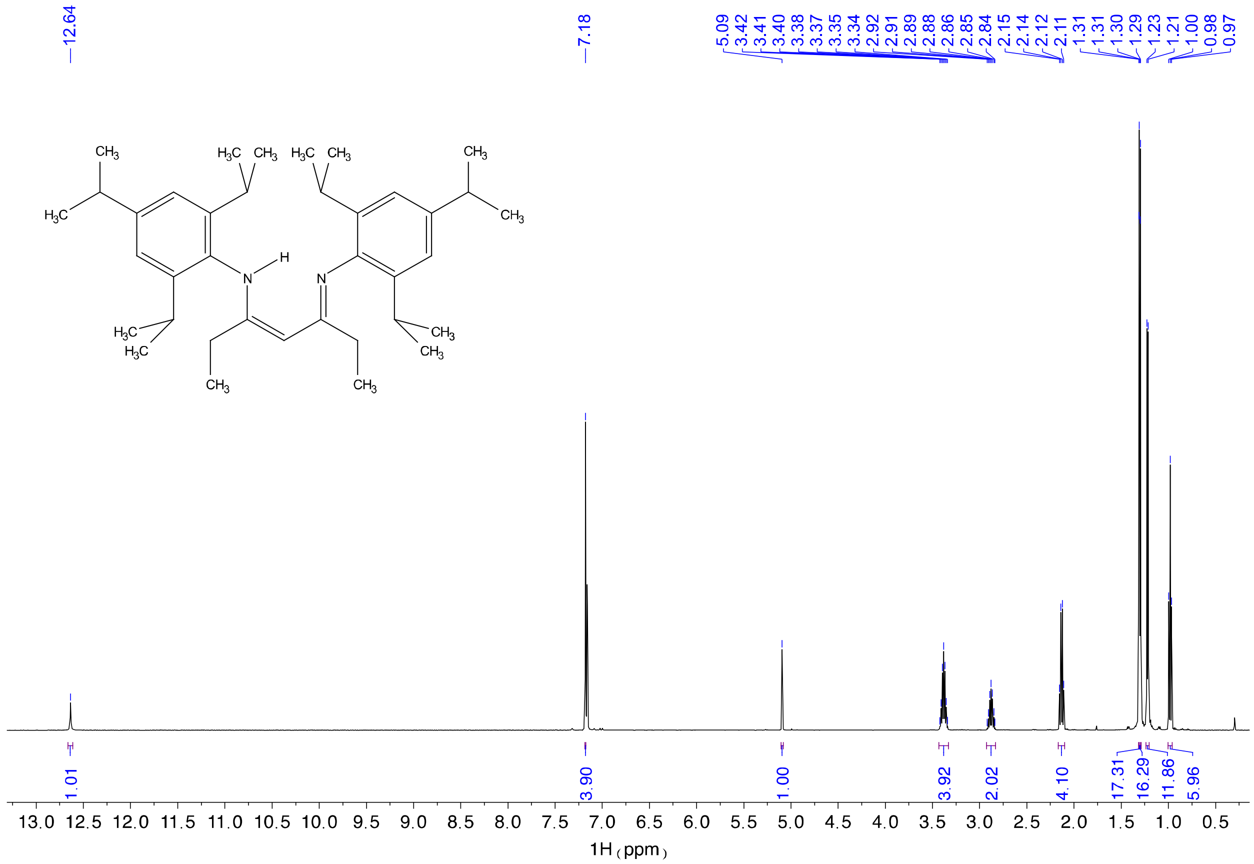

Figure S30. ^{1}H NMR spectrum (499.9 MHz, C_6D_6, 298 K) of ^{EtTrip}nacnacH **19**.

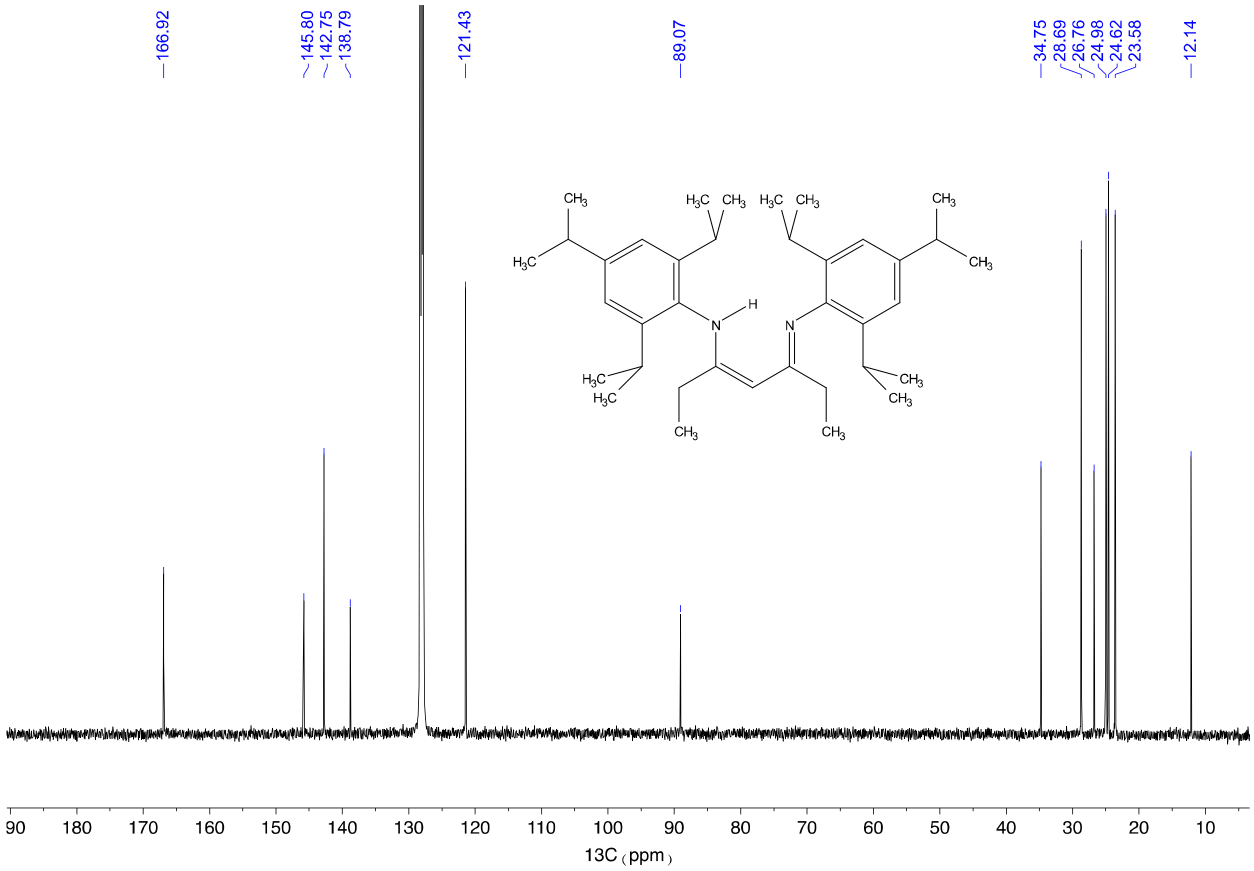

Figure S31. $^{13}C\{^{1}H\}$ NMR spectrum (125.7 MHz, C_6D_6, 298 K) of ^{EtTrip}nacnacH **19**.

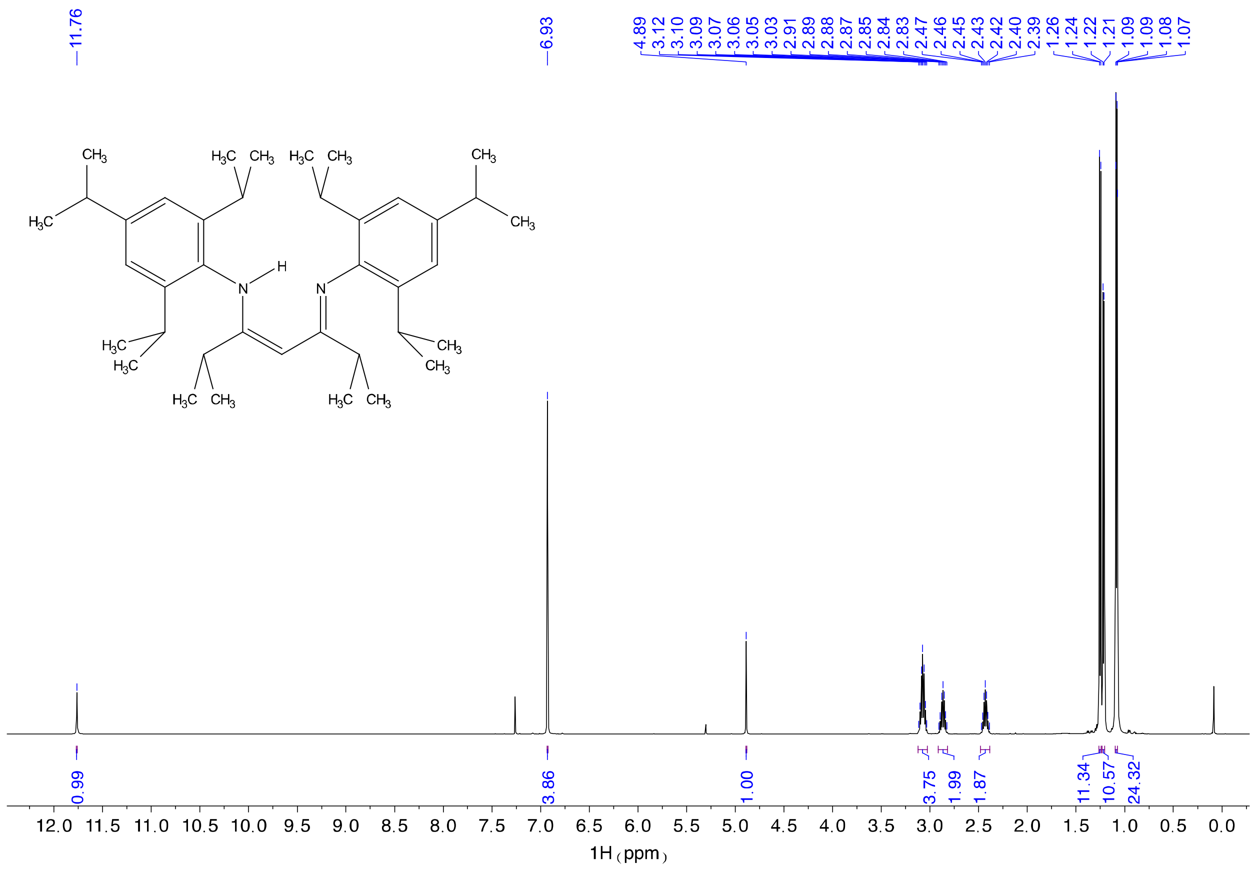

Figure S32. ^{1}H NMR spectrum (500.1 MHz, $CDCl_3$, 295 K) ^{iPrTrip}nacnacH **20**.

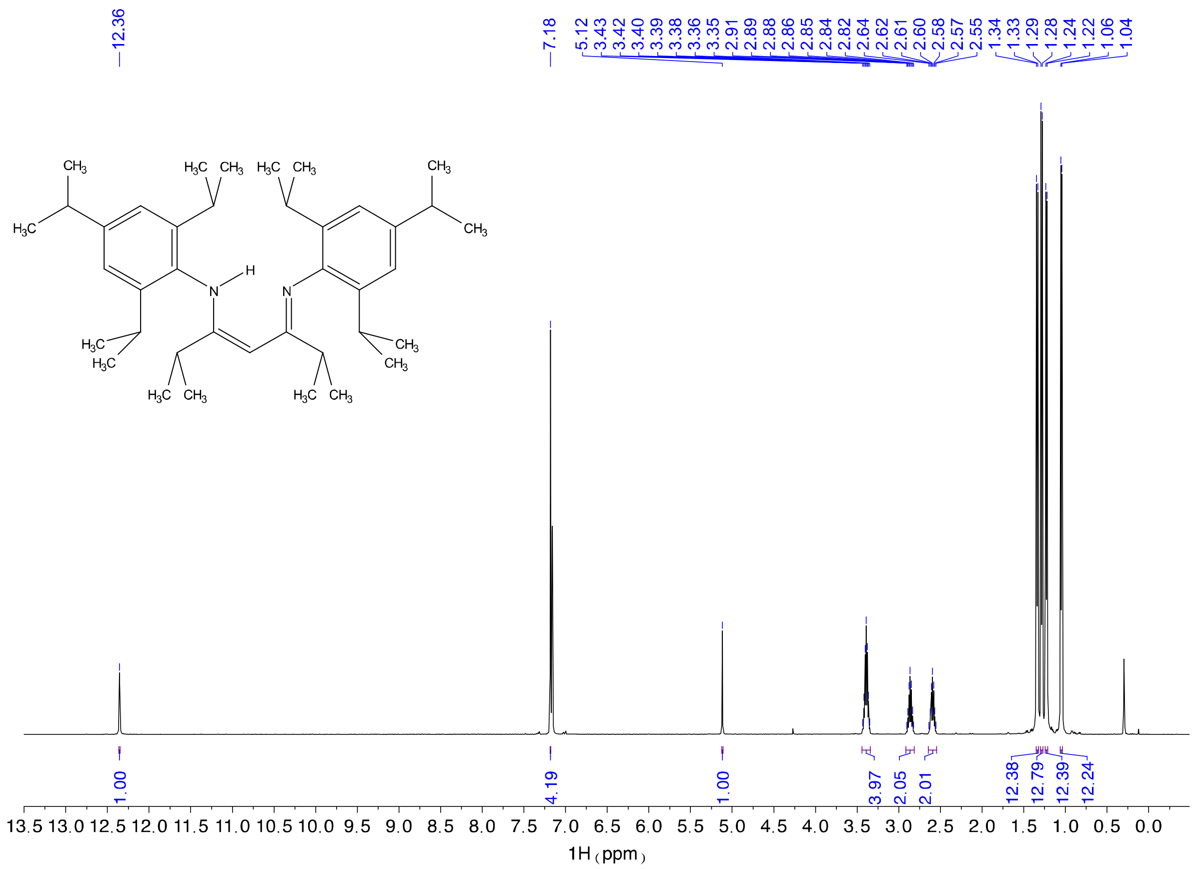

Figure S33. ^{1}H NMR spectrum (499.9 MHz, C_6D_6, 298 K) ^{iPrTrip}nacnacH **20**.

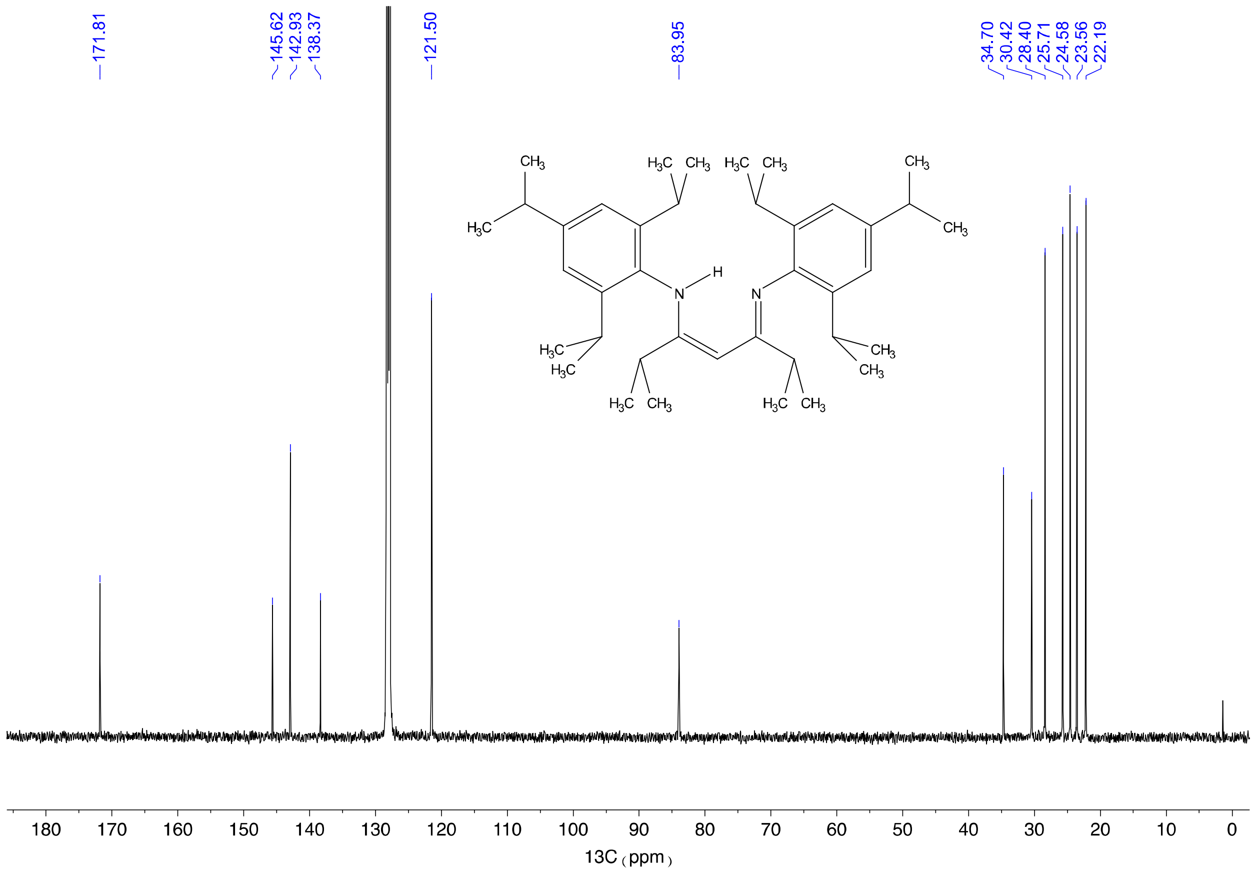

Figure S34. $^{13}C\{^{1}H\}$ NMR spectrum (125.7 MHz, C_6D_6, 298 K) ^{iPrTrip}nacnacH **20**.

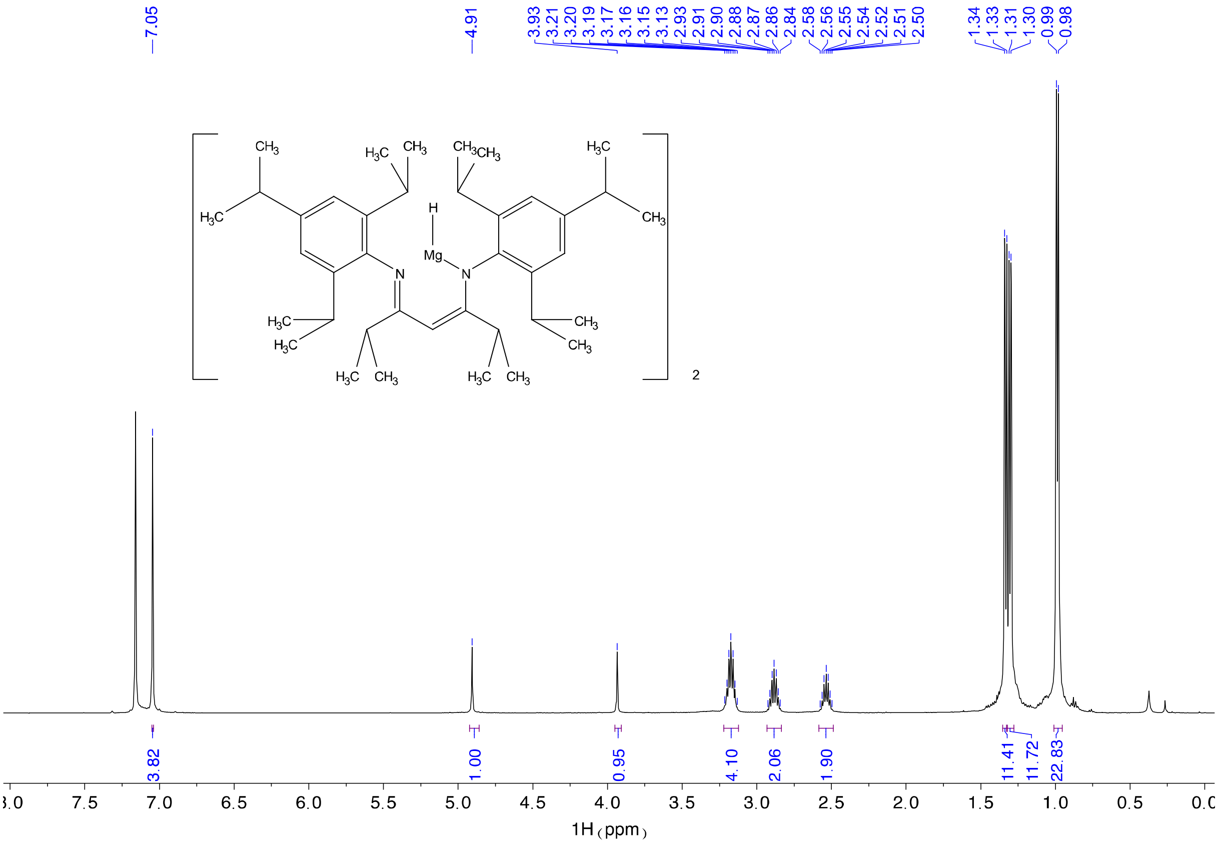

Figure S35. ^{1}H NMR spectrum (499.9 MHz, C_6D_6, 353 K) of [{(^{iPrTrip}nacnac)MgH}$_2$] **21**.

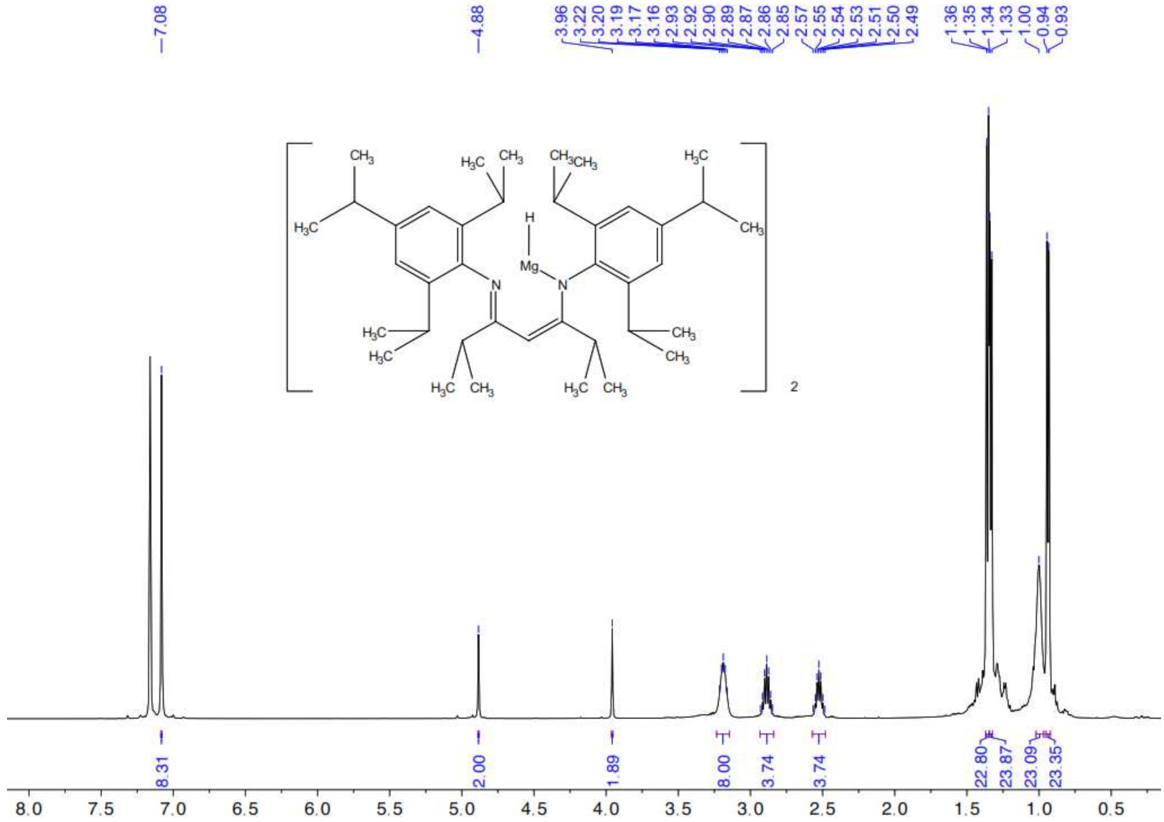

Figure S36. ^{1}H NMR spectrum (499.9 MHz, C_6D_6, 298 K) of [{(^{iPrTrip}nacnac)MgH}$_2$] **21**.

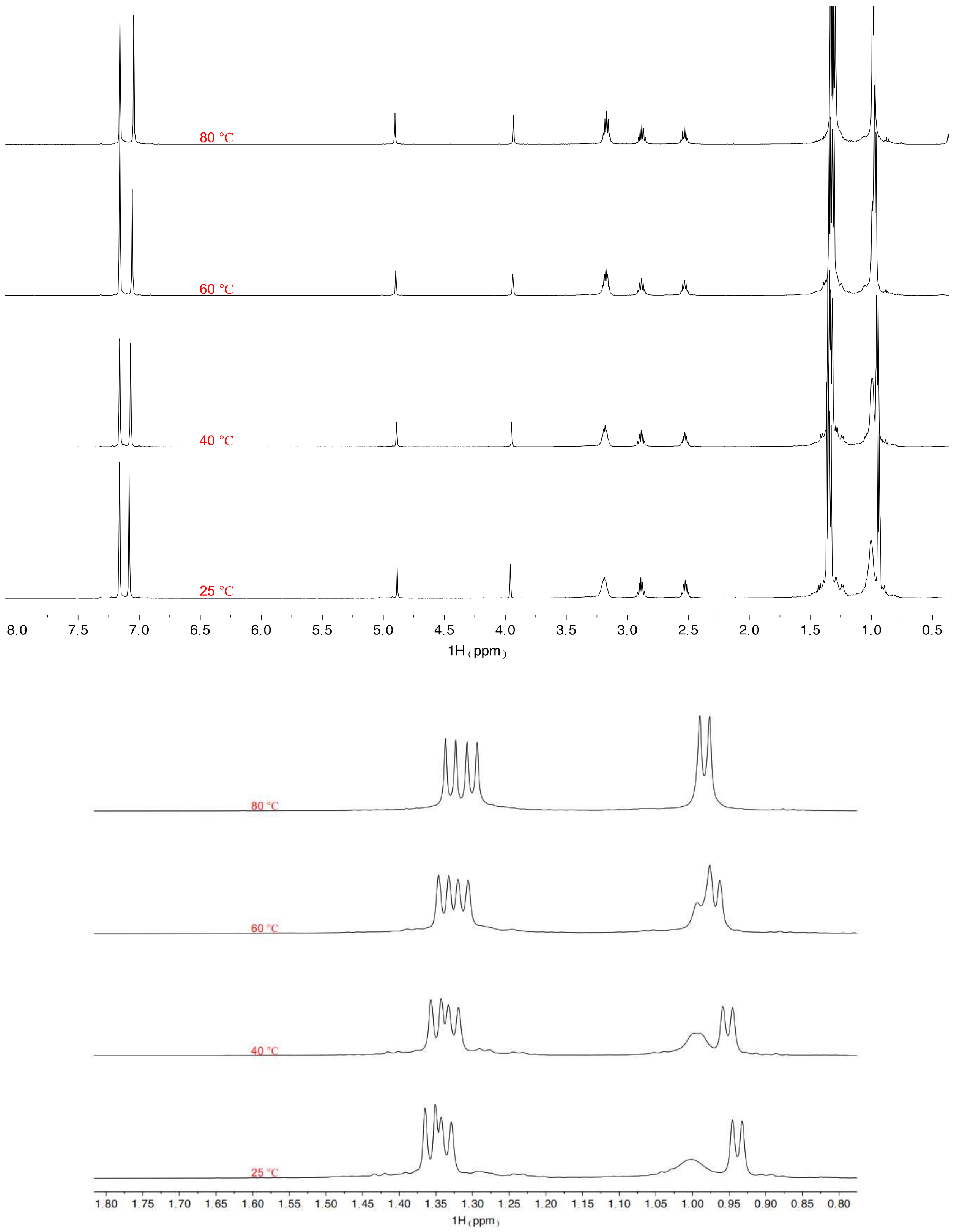

Figure S37. Stacked variable temperature ^{1}H NMR spectra (499.9 MHz, C_6D_6,) of [{(^{iPrTrip}nacnac)MgH}$_2$] **21**.

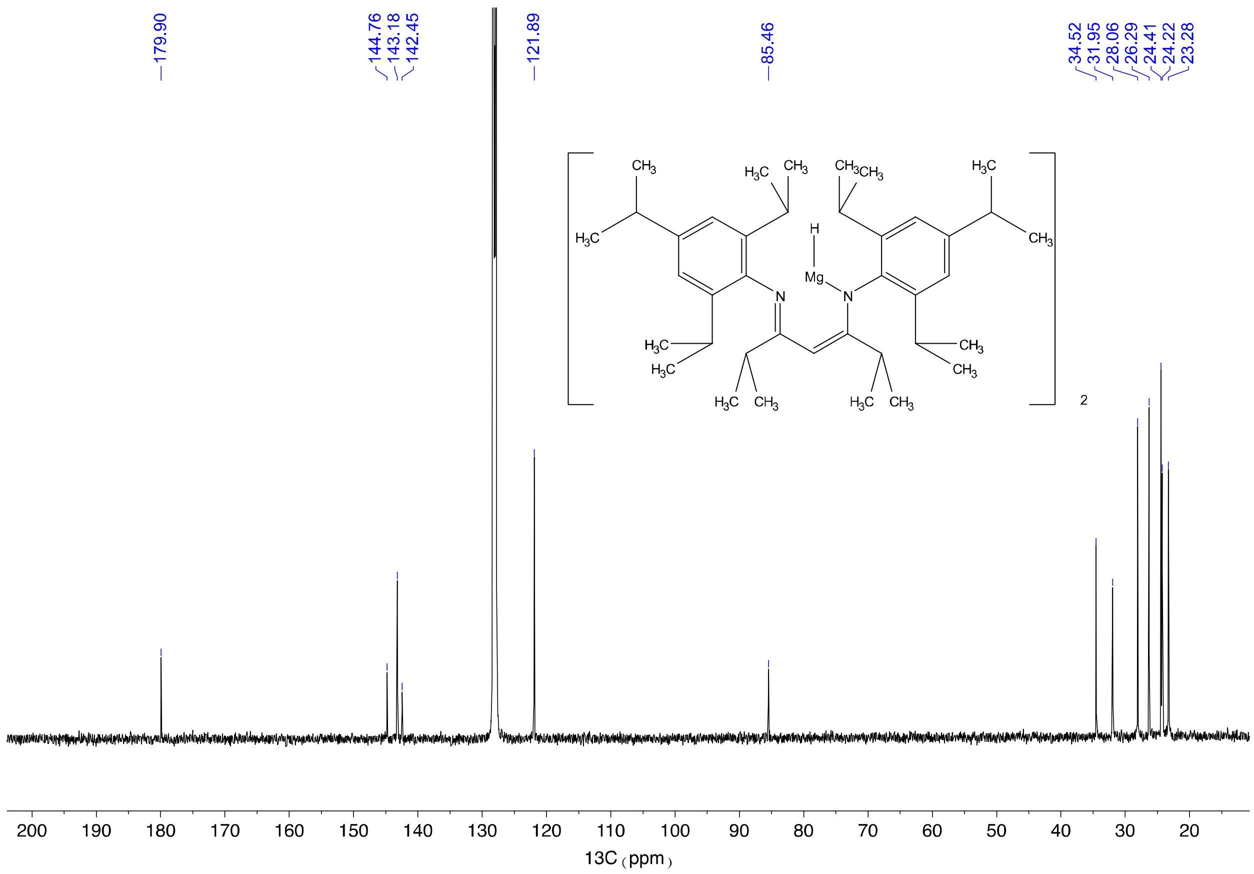

Figure S38. $^{13}C\{^1H\}$ NMR spectrum (125.7 MHz, C_6D_6, 353 K) of $[\{(^{iPrTrip}nacnac)MgH\}_2]$ **21**.

4 Buried volume information

A buried volume (V_{buried}) comparison was undertaken for the species [{(^{RDip}nacnac)MgH}$_2$] with R = Me, *i*Pr and *t*Bu and [{(^{iPrTrip}nacnac)MgH}$_2$] **21**, please see the main text for further details.

Table S2. Buried volume for [{(^{RAr}nacnac)MgH}$_2$] complexes for the four quadrants and in total.

**[{(^{RAr}nacnac)MgH}$_2$]**	V_{buried} (SW)	V_{buried} (NW)	V_{buried} (NE)	V_{buried} (SE)	**V_{buried} total**
R = Me, Ar = Dip	43.7%	44.0%	57.7%	59.0%	**51.1%**
R = *i*Pr, Ar = Dip (Mg1)	47.9%	43.5%	60.9%	64.8%	**54.3%**
R = *i*Pr, Ar = Dip (Mg2)	43.4%	47.1%	64.7%	63.8%	**54.8%**
R = *t*Bu, Ar = Dip (Mg1)	44.6%	43.2%	71.8%	60.7%	**55.1%**
R = *t*Bu, Ar = Dip (Mg2)	49.4%	47.3%	67.7%	60.9%	**56.3%**
R = *i*Pr, Ar = Trip (**21**)	47.7%	56.6%	50.0%	59.8%	**53.5%**

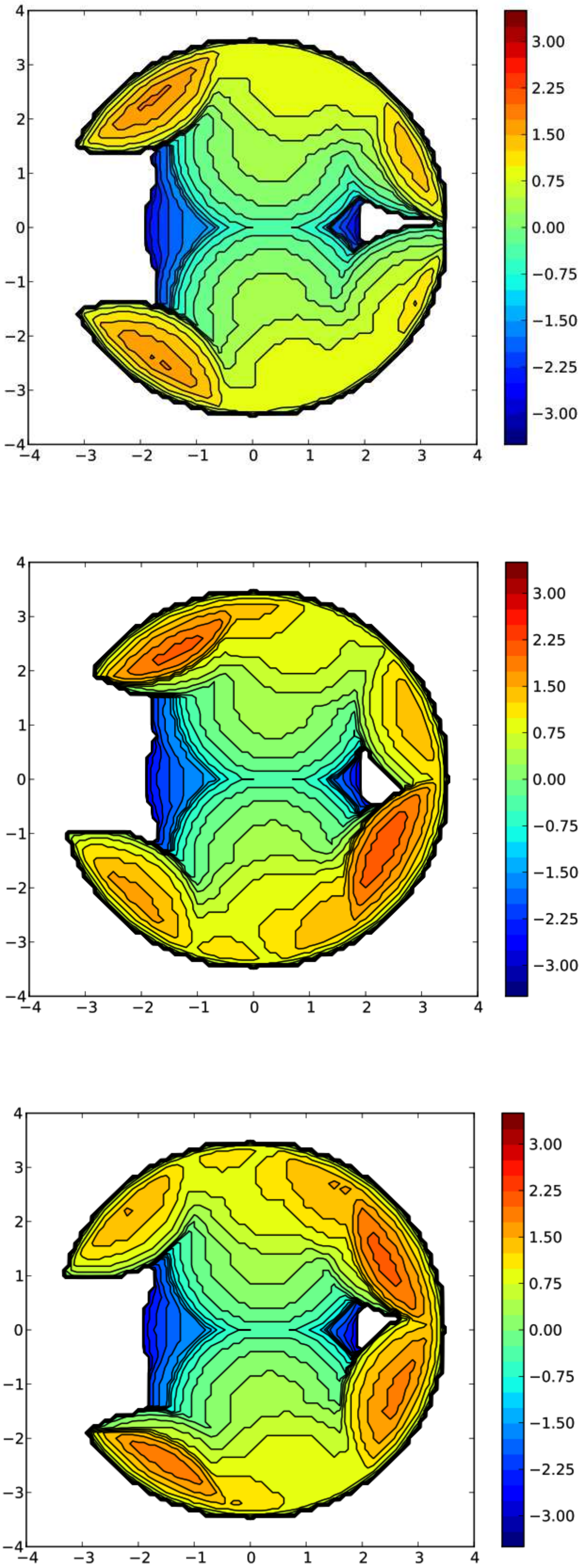

Figure S39. Steric maps (Mg at the centre, N atoms above and below the centre) for [{(^{MeDip}nacnac)MgH}$_2$] (top) and [{(^{MeDip}nacnac)MgH}$_2$] (middle for Mg1, bottom for Mg2).

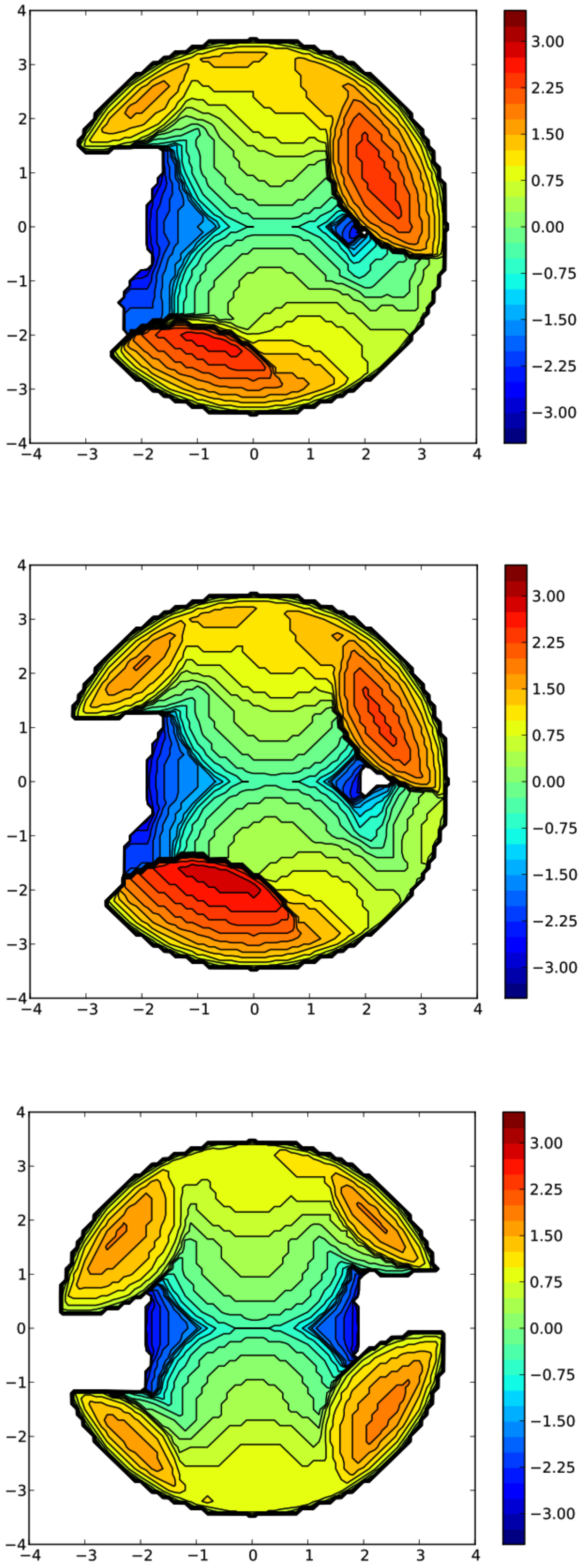

Figure S40. Steric maps (Mg at the centre, N atoms above and below the centre) for [{(^{tBuDip}nacnac)MgH}$_2$] (top for Mg1, middle for Mg2) and [{(^{iPrTrip}nacnac)MgH}$_2$] **21** (bottom).
